# Whole-genome characterization and pathogenicity of novel human-porcine reassortant rotavirus strains G9P[7] and G1P[7] in China

**DOI:** 10.1186/s13567-026-01775-1

**Published:** 2026-07-15

**Authors:** Meizhen Li, Mengli Qiao, Keshun Bao, Yuanhang Zhang, Panchi Zhang, Jing Chen, Qi Luan, Kun Li, Li Wang, Bin Zhou

**Affiliations:** 1 MOE Joint International Research Laboratory of Animal Health and Food Safety, College of Veterinary Medicine, Nanjing Agricultural University, Nanjing, China; 2 Key Laboratory of Animal Bacteriology, Ministry of Agriculture and Rural Affairs, Nanjing Agricultural University, Nanjing, China; 3 College of Veterinary Medicine, Northeast Agricultural University, Harbin, China; 4 Northeast Science Observation Station for Animal Pathogen Biology, Ministry of Agriculture and Rural Affairs, Harbin, China

**Keywords:** PoRVA, reassortment, recombination, pathogenicity, pulmonary tropism, zoonotic risk

## Abstract

The prevalence of porcine rotavirus A (PoRVA) in China has increased significantly, threatening the swine industry. Pigs serve as “mixing vessels” for rotaviruses, facilitating reassortment between human and animal strains, leading to the emergence of zoonotic variants. In this study, two PoRVA strains, XXW2023 (G9P[7]) and HD2023 (G1P[7]), were isolated from diarrheic piglets in Guangdong Province, China. Genomic analysis revealed that both strains were human-porcine reassortants, with VP1, VP3, and NSP1 genes closely related to human rotaviruses. Intragenic recombination was identified in the VP4 and VP6 genes. Pathogenicity was evaluated in 7-day-old mice and 1-day-old piglets. Both strains caused persistent diarrhea in mice and severe watery diarrhea, intestinal lesions, and death within 48 h in piglets. Systemic infection was confirmed, with viral replication detected in the lungs. Infectious virus titers, VP6 antigen, and NSP4 were detected in lung tissues, providing evidence of active replication in the respiratory tract. The strains exhibited distinct tissue tropism, with XXW2023 being enterotropic and HD2023 showing pulmonary tropism. Viral RNA and antigen levels in the lungs of HD2023-infected piglets exceeded those in their intestines, and the infectious virus titer in their lungs was significantly higher than that in the lungs of XXW2023-infected piglets. These findings demonstrate that reassortant rotavirus strains infect the respiratory tract, extending the conventional view of rotavirus as a strictly enteric pathogen. The emergence of these highly pathogenic, phenotypically divergent human-porcine reassortants underscores their zoonotic risk, highlighting the need for enhanced surveillance and reconsideration of vaccine coverage.

**Supplementary Information:**

The online version contains supplementary material available at https://doi.org/10.1186/s13567-026-01775-1.

## Introduction

Rotavirus A (RVA), a member of the *Reoviridae* family, is a leading cause of diarrheal disease in infants, young children, and juvenile animals. RVA infects more than 258 million children under five globally each year, resulting in more than 2 million hospitalizations [1]. In swine production, epidemiological surveys place RVA second only to porcine epidemic diarrhea virus (PEDV) in detection rates, underscoring its status as a significant threat to herd health [2]. RVA frequently co-infects with other viruses and bacteria, such as *Escherichia coli*, leading to high rates of diarrhea dehydration, and mortality in piglets, resulting in substantial economic losses [2, 3].

Rotaviruses have an 11-segmented double-stranded RNA genome encoding 6 structural proteins (VP1-4, VP6, and VP7) and 6 nonstructural proteins (NSP1-NSP5/6) [4]. The International Committee on Taxonomy of Viruses (ICTV) has classified rotaviruses into nine distinct species (A-D and F-J) on the basis of the antigenicity of the VP6 protein [5, 6]. RVA commonly infects humans, pigs, and other animals, with frequent recombination and interspecies transmission among RVAs [7]. RVA strains from different hosts undergo recombination during coinfection, a significant evolutionary mechanism that enhances viral diversity [8]. Cross-species transmission of RVAs is also a principal factor influencing viral evolution [8, 9]. The capsid proteins VP4 and VP7 elicit neutralizing antibodies, which are crucial for immune protection and vaccine development. A whole-genome genotyping classification system has been proposed, introducing standardized nomenclature (Gx-P[x]-Ix-Rx-Cx- Mx-Ax-Nx-Tx-Ex-Hx) corresponding to the VP7-VP4-VP6-VP1-VP2-VP3- NSP1-NSP2-NSP3-NSP4-NSP5/6 genes, refining the genomic classification of novel RVA strains [10]. Genotypic diversity in RVA strains primarily arises from genetic and antigenic drift due to point mutations, homologous recombination, and interspecies transmission. This process occurs when strains infecting heterologous hosts undergo reassortment, generating novel genotypes [11].

Pigs serve as animal hosts for zoonotic RVA, with suckling and weaned piglets vulnerable to infection that causes acute gastroenteritis [4]. Pigs are considered the primary species through which rotaviruses cross the species barrier into humans, with some strains undergoing interspecies transmission and genetic recombination with concurrent human strains [12]. In China, surveillance data indicate that porcine RVA G9 is increasing (56.55%) and has surpassed G5 (14.48%) to become the predominant genotype [13]. Experimental infections further show that porcine G9 strains, including G9P[23], cause diarrhea and intestinal lesions with villous atrophy, confirming pathogenicity [14]. Comparative studies also suggest that porcine G9 differs from commonly used human G1 vaccine strains in piglet models, including prolonged virus shedding. Phylogenetic analyses additionally imply potential zoonotic spillover. However, evidence remains limited regarding lineage-specific virulence, immune-evasion mechanisms, and interactions with prevalent P types, including P[23].

Meanwhile, the porcine RVA G1 genotype (8.97%), an emerging genotype in swine, has shown an increasing detection rate [13]. Although G1 is one of the predominant genotypes in human rotavirus infections, it has historically been uncommon in pig populations [3]. Recent studies have reported novel porcine reassortant G1P[7] strains in China, highlighting the need for close monitoring of G1 emergence in swine [15]. However, compared with genotype G9, data on the pathogenicity of porcine G1 strains remain extremely limited. Notably, there is a complete lack of experimental evidence defining the infectious dose, clinical disease severity, and tissue tropism of G1 strains in piglets, as well as a lack of head-to-head comparisons with other prevalent porcine genotypes, such as G9 and G5. This knowledge gap hampers accurate assessment of the threat posed by G1 to swine health and constrains the development of effective vaccines targeting this emerging genotype. Moreover, newly emerging strains may compromise heterotypic immunity and thereby reduce the protective efficacy of existing rotavirus vaccines.

Animal infection models are essential for elucidating rotavirus pathogenic mechanisms and for evaluating vaccine protective efficacy. In recent years, for newly emerging porcine rotavirus (PoRV) strains that continue to circulate, such as G9P[23] and G11P[7], investigators have similarly employed neonatal piglet infection models to evaluate their high pathogenicity, highlighting the critical role of animal models in understanding the risks posed by currently circulating strains [16]. Accordingly, to clarify PoRV genetic evolution and molecular diversity, we isolated two novel strains, XXW2023 (G9P[7]) and HD2023 (G1P[7]). Using whole-genome analyses, we inferred their evolutionary origins, recombination patterns, and relationships to reference strains, and characterized key neutralizing epitopes. We established infection models in suckling mice and neonatal piglets to directly compare genotype-specific differences in clinical pathogenicity, systemic infection patterns, and distinct pulmonary versus intestinal tropism. Our findings clarify core genetic features and pathogenic mechanisms of circulating PoRVA, informing interpretations of epidemiological dynamics and supporting improved prevention and control strategies. Additionally, the isolated strains serve as candidate challenge viruses and a foundation for developing vaccines targeting prevalent PoRVA variants.

## Materials and methods

### Sample preparation, cells, and antibodies

In 2023, anal swab samples were collected from diarrheic piglets at a commercial pig farm in Guangdong Province, China. Total RNA was extracted from sample suspensions using the Viral RNA Extraction Kit (Vazyme, Nanjing, China) and reverse-transcribed into cDNA, which was stored at −20 °C. The sample was positive for PoRVA as determined by probe-based quantitative reverse transcription polymerase chain reaction (RT-qPCR) targeting the NSP3 gene. Further probe-based RT-qPCR assays for porcine epidemic diarrhea virus (PEDV), transmissible gastroenteritis virus (TGEV), and porcine deltacoronavirus (PDCoV) were all negative, and primer sequences are provided in Table 1. RT-qPCR was performed in a 20 µL reaction mixture containing 10 µL of 2 × AceQ qPCR Probe Master Mix (Vazyme, Nanjing, China), 0.4 µL each of 10 µM forward and reverse primers, 0.2 µL of 10 µM TaqMan probe, 2 µL of RNA template, and 7 µL of nuclease-free water, and amplification was carried out on a QuantGene 9600 real-time PCR system (Bioer Technology, China) with pre-denaturation at 95 °C for 5 min, followed by 40 cycles of 95 °C for 10 s and 60 °C for 30 s.

**Table 1:** **Primer and probe sequences in this study**

Primer	Primer sequences (5′ → 3′)	Product (bp)
RVA-NSP3-F	ACCATCTACACATGACCCTCTATGAG	83
RVA-NSP3-R	ACATAACGCCCCTATAGCCATTTAG	
RV- NSP3-Probe	FAM-ACAATAGTTAAAAGCTAACACTG-BHQ1	
RVA-VP6-F	GGCTTTWAAACGAAGTCTTC	1356
RVA-VP6-R	GGTCACATCCTCTCACTA	
RVA-VP7-F	GGCTTTAAAAGAGAGAATTTCCGTCTGG	395
RVA-VP7-R	ACTGATCCTGTTGGCCATCCTTT	
PEDV-F	GACGCGCTTCTCACTACTTC	134
PEDV-R	TGTACGCCAGTAGCAACCTT	
PEDV-Probe	FAM-TGCAGACCTGTCGGCCCATCA-BHQ1	
TGEV-F	ACATAGTGGGTGTACCGTCTG	140
TGEV-R	GCCACTAAGTAGCGTCCTGT	
TGEV-Probe	CY5-AGCACTGACAAATCGTGCACACCA-BHQ2	
PDCoV-F	CAGTTTCGTGGCAATGGAGT	79
PDCoV-R	TGGTGTAACGCAGCCAGTAG	
PDCoV-Probe	HEX-CCGCTTAACTCCGCCATCAAACCCG-BHQ1	

For detection of the VP6 gene from infectious virus at passage 7, RT-PCR was performed using an Eppendorf Mastercycler® nexus X2 thermal cycler. The 20 µL reaction mixture consisted of 10 µL of 2 × Rapid Taq Master Mix (Vazyme, Nanjing, China), 1 µL each of 10 µM forward and reverse primers, 2 µL of cDNA template, and 6 µL of nuclease-free water. The amplification protocol consisted of an initial denaturation at 95 °C for 3 min, followed by 35 cycles of denaturation at 95 °C for 15 s, annealing at 55 °C for 15 s, and extension at 72 °C for 30 s, with a final extension at 72 °C for 5 min.

Samples were centrifuged at 10 000 rpm for 10 min at 4 °C. The supernatant was filtered through a 0.22 μm filter (Merck Millipore, Darmstadt, Germany), supplemented with 1% penicillin–streptomycin solution (10 000 U/mL penicillin, 10 000 μg/mL streptomycin; SAITONG, Beijing, China), and stored at −80 °C for virus isolation.

MA104 cells maintained in our laboratory were cultured in medium supplemented with 10% inactivated fetal bovine serum (Gibco™, New York, NY, USA) and 1% penicillin–streptomycin solution (SAITONG, Beijing, China). The mouse monoclonal antibody against PoRVA VP6 protein was kindly provided by Dr Bin Li from Jiangsu Academy of Agricultural Sciences and used at a dilution of 1:2000 for immunofluorescence assay (IFA) and 1:200 for immunohistochemistry (IHC); the antibody against NSP4 was kindly provided by Dr Xuehan Zhang from the same academy and used at a dilution of 1:1000 for IHC.

### Animals and husbandry conditions

A total of 12 7-day-old specific pathogen-free (SPF) BALB/c suckling mice (6 females and 6 males) were procured from Jiangsu Qinglongshan Biotechnology Co., Ltd. To ensure adequate nursing, each group of suckling mice was assigned one SPF-grade lactating female mouse. All animals were transferred to a barrier-maintained facility at the Animal Experiment Center of Nanjing Agricultural University 1 day prior to viral challenge. The mice were housed in a temperature-controlled environment (26–28 °C), and the nursing dams were provided with a standard laboratory rodent diet. To minimize the risk of cross-contamination, all personnel were required to don sterile personal protective equipment (PPE) prior to entering the animal housing area, and strict physical separation between experimental groups was maintained throughout the study. All procedures involving mice were performed in compliance with the institutional animal welfare guidelines of Nanjing Agricultural University and national ethical regulations, with prior approval from the Animal Experiment Ethics Committee (approval number: No20241021209).

A total of 15 healthy 1-day-old conventional (non-gnotobiotic) Duroc × Landrace × Large White piglets (8 females and 7 males) were sourced from the Acheng Test Base of Northeast Agricultural University (Harbin, China). To prevent intergroup transmission and protect the piglets from unrelated infections, they were maintained in sterile, temperature-regulated isolators set at 30–34 °C and were fed a commercial milk replacer formulated to simulate maternal nutrition. Throughout the experimental period, strict biosecurity protocols were enforced, including the use of sterile protective clothing and physical isolation of groups. All experimental procedures involving piglets adhered to the ethical guidelines of Northeast Agricultural University and complied with national regulations for animal research. Ethical approval was granted by the Animal Experiment Ethics Committee of Northeast Agricultural University (approval number: NEAUEC202503104).

### Virus isolation

The rotavirus isolation method described by Almeida et al. was applied with minor modifications [17]. Cells were washed three times with sterile phosphate-buffered saline (PBS, pH 7.4) to remove excess fetal bovine serum (FBS). The activated inoculum, containing 10 µg/mL trypsin, was inoculated into MA104 cells. After incubation at 37 °C for 2 h, the inoculum was discarded, and maintenance medium (Dulbecco’s Modified Eagle Medium [DMEM] with 0.5 µg/mL trypsin) was added. The inoculated cells were placed in a 37 °C incubator with 5% CO_2_. Cell changes were observed daily, with a 72 h passage cycle. The cell culture was collected, freeze-thawed three times, and then centrifuged at 12000 rpm for 5 min at 4 °C. The supernatant was subsequently inoculated into the cells. The virus was serially passaged in MA104 cells for 5–6 passages (72 h per passage) until a stable cytopathic effect (CPE) was observed. The resulting culture supernatant was then subjected to plaque assay and plaque purification. Viral titers were then determined by plaque assay. Briefly, a tenfold serial dilution of the viral suspension was prepared in DMEM containing 10 µg/mL trypsin (final concentration). Dilutions ranging from 10^2^ to 10^7^ were inoculated onto confluent MA104 cell monolayers in 6-well plates. After 2 h of adsorption at 37 °C with 5% CO_2_, the inoculum was removed, and the cells were overlaid with 2 mL of an overlay medium consisting of DMEM with 0.5 µg/mL trypsin (final concentration) and 2% low-melting-point agarose. Plates were incubated at 37 °C with 5% CO_2_ for 3–5 days. Upon plaque development, the monolayers were stained with 0.1% neutral red, and a single well-isolated plaque was picked. Each plaque pick was subjected to three freeze–thaw cycles, and the clarified supernatant was used to initiate the next round of plaque assay. Three consecutive rounds of plaque purification were performed. To confirm the clonal nature of the purified virus, viral RNA was extracted from the final stock (obtained after the third round of plaque purification) using a Viral RNA Extraction Kit (Vazyme, Nanjing, China). A fragment of the VP7 gene was amplified by RT-PCR using specific primers listed in Table 1 under the same amplification conditions as described in the Sample preparation, cells, and antibodies section. The PCR product was purified and verified by Sanger sequencing (Tsingke Biotechnology Co., Ltd.). The virus stock obtained after the third round of plaque purification and sequence confirmation was used as the seed virus to establish subsequent passage cultures for further experiments.

### IFA

MA104 cells were seeded in 12-well culture plates. After the cells formed a confluent monolayer, they were inoculated with PoRVA isolates. At 24 h post-infection (hpi), the culture medium was discarded. The cells were then fixed with 4% paraformaldehyde solution at 4 °C for 30 min, followed by permeabilization with 0.5% Triton X-100 at room temperature for 10 min. Cells were incubated overnight at 4 °C with the PoRVA-VP6 monoclonal primary antibody, followed by a 45 min incubation at 37 °C with an Alexa Fluor® 488-conjugated goat anti-mouse secondary antibody. Nuclei were stained with 4′,6-diamidino-2-phenylindole (DAPI) at room temperature for 5 min. Three washes with PBS buffer were performed after each step. The cells were finally observed under an inverted fluorescence microscope (Axiovert A1, ZEISS, Germany).

### Transmission electron microscopy (TEM)

MA104 cells were infected at a multiplicity of infection (MOI) of 0.01. Cells were harvested when the CPE exceeded 80%, and then centrifuged at 12000 rpm for 30 min at 4 °C to remove cellular debris. The virus suspension was adjusted to final concentrations of 10% PEG6000 and 0.5 M NaCl and incubated overnight at 4 °C on a rocking platform. After centrifugation at 40000 × *g* for 2 h at 4 °C, the supernatant was discarded, and the pellet was resuspended in PBS. The resulting virus suspension was purified by centrifugation through a stepwise sucrose gradient (10%, 30%, 40%, 50%, and 60% sucrose) at 40000 × *g* for 6 h at 4 °C, and the visible band at the interface between the 40% and 50% sucrose layers was collected. The collected sucrose fraction containing PoRVA was diluted with PBS and centrifuged at 40000 × *g* for 2 h at 4 °C. The final pellet was resuspended in a small volume of PBS, yielding purified PoRVA. Virion morphology was assessed by negative-stain transmission electron microscopy (H7700, Hitachi, Japan).

### Virus growth kinetics

Using a MOI of 0.01, P10 viruses of both strains were inoculated into MA104 cells that had been pre-seeded in 6-well plates. Supernatants were collected at 6, 12, 18, 24, 30, 36, 42, 48, and 54 hpi. Viral titers at each timepoint were determined using the 50% tissue culture infectious dose (TCID_50_) assay, calculated by the Reed–Muench method [18]. For serial dilutions, 100 µL of supernatant from each timepoint was mixed with 900 µL DMEM to prepare tenfold dilutions (10^–1^–10^–10^). Aliquots (100 µL) of each dilution were added to 96-well plates pre-seeded with confluent MA104 monolayers. Plates were incubated at 37 °C with 5% CO_2_ for 4 days. The CPE was assessed microscopically to determine the titers. Finally, growth curves for both viruses were generated in GraphPad Prism 10.0 from titers at the indicated timepoints.

### Whole-genome sequencing and genetic evolutionary analysis

Viral RNA was extracted from purified XXW2023 (G9P[7]) and HD2023 (G1P[7]) isolates using the FastPure Cell/Tissue Total RNA Isolation Kit V2 (Vazyme, Nanjing, China) and then used to prepare libraries for next-generation sequencing (NGS). Library preparation and Illumina sequencing were conducted by Shanghai Tuanpu Biotechnology Co., Ltd. (Shanghai, China). Libraries were prepared with the NEBNext Ultra II RNA Library Prep Kit (NEB), followed by adapter ligation and target enrichment using 10 PCR cycles. Libraries were pooled equimolarly, denatured, and diluted to the manufacturer-recommended loading concentration. Sequencing on an Illumina NovaSeq 6000 (Illumina, San Diego, CA, USA) generated 150 bp paired-end reads. Bioinformatic processing was performed according to published protocols [16]. Genotype assignment was performed using the online platform of the Bacterial and Viral Bioinformatics Resource Center (BV-BRC) [19]. To infer the evolutionary origins of XXW2023 (G9P[7]) and HD2023 (G1P[7]), the PoRVA gene segments obtained in this study were analyzed together with GenBank reference sequences. Phylogenetic trees were inferred in MEGA X using the neighbor-joining (NJ) method with 1000 bootstrap replicates and default settings. To enhance presentation, circular tree layouts were formatted and visualized in tvBOT v2.6.1 [20]. Pairwise nucleotide identity was assessed in SDT v1.3 to compare our strains with representative domestic and international isolates.

### Recombination and antigenic epitope analysis

Putative recombination across the viral genome was screened in RDP v4.101 under default parameters. Candidate events were verified, and breakpoint profiles were examined in SimPlot v3.5.1 using the inferred major and minor parental references. To assess antigenic consequences, amino acid substitutions within defined neutralizing epitopes were mapped in Jalview for VP4 (VP5*, VP8*; 8-1 to 8-4) and VP7 (7-1 and 7-2), given that VP4 and VP7 are the principal neutralization antigens.

### Pathogenicity assessment of isolated PoRVA strains XXW2023 and HD2023 in suckling mice

To assess the pathogenicity of PoRVA strains in suckling BALB/c mice, 12 7-day-old specific pathogen-free (SPF) mice were randomly assigned to 3 groups (*n* = 4 per group). Two groups were orally inoculated with 100 μL of the tenth-passage PoRVA isolates, XXW2023 (G9P[7]) or HD2023 (G1P[7]),at a titer of 10^7.25^ TCID_50_/mL, whereas the control group received an equivalent volume of DMEM. Clinical signs, including diarrhea and viral shedding, were systematically monitored and recorded at 12-h intervals. Fecal consistency was evaluated using a standardized four-grade scoring system: 0, normal feces; 1, soft feces; 2, soft, yellowish feces; and 3, watery, yellowish diarrhea. Fecal specimens were collected from each group and immediately stored at −80 °C for subsequent analysis of viral shedding. At the designated experimental endpoint, mice were randomly euthanized, and intestinal tissues were harvested for viral load quantification via RT-qPCR and for histopathological evaluation by hematoxylin and eosin (HE) staining.

### Pathogenicity assessment of isolated PoRVA strains XXW2023 and HD2023 in piglets

For pathogenicity evaluation in piglets, 15 1-day-old healthy Duroc × Landrace × Large White piglets with no recent history of diarrhea were obtained from the Acheng Test Base of Northeast Agricultural University (Harbin, China). Prior to inoculation, all piglets were confirmed negative for major enteric pathogens, including PoRV, PEDV, TGEV, and PDCoV by RT-qPCR screening. The animals were randomly allocated into two infection groups (*n* = 6 per group) and one control group (*n* = 3). Each infection group was orally administered 4 mL of a tenth-passage PoRVA isolate (10^7.25^ TCID_50_/mL), whereas the control group received an equal volume of DMEM. Piglets were maintained on a commercial milk replacer, administered every 4 h, and observed at 12-h intervals for clinical manifestations, including diarrhea and viral shedding. Fecal consistency was assessed using established criteria [16]: 0, solid feces; 1, paste-like feces; 2, semi-liquid feces; and 3, watery diarrhea. Fecal samples were collected periodically and stored at −80 °C for subsequent quantification of viral shedding.

At the peak of viral shedding, two piglets from each infected and control group were randomly euthanized for pathological evaluation. To avoid cross-contamination, biological samples were collected in a defined order: blood, lung, mesenteric lymph node (MLN), stomach, and small intestine. The harvested tissues were processed for RT-qPCR, HE staining, and immunohistochemical (IHC) analyses to assess viral load, histopathological changes, and tissue-specific antigen distribution.

To further confirm the presence of infectious virus in the lungs, virus titration was performed. Lesioned lung tissues were homogenized in sterile DMEM (10% w/v), subjected to three freeze–thaw cycles, and centrifuged at 10000 rpm for 10 min at 4 °C. The supernatant was filtered through a 0.22 μm membrane and inoculated onto confluent cell monolayers (as described in Virus isolation). After incubation at 37 °C with 5% CO_2_, cultures were blind-passaged three times. Viral titers were subsequently determined by TCID_50_ assay following the protocol in Virus growth kinetics and reported as log_10_ TCID_50_/mL.

### Real-time quantitative RT-qPCR

Fresh fecal swabs from suckling mice and piglets were collected every 12 h and resuspended in PBS (200 µL for mice; 1 mL for piglets) for viral RNA extraction. Tissue and organ samples were obtained from five to six sites per specimen, minced, homogenized, and processed using the FastPure Cell/Tissue Total RNA Isolation Kit V2 (Vazyme, Nanjing, China) to isolate RNA from fecal swabs, blood, and tissue homogenates. The extracted RNA was reverse-transcribed into cDNA and subsequently subjected to TaqMan RT-qPCR analysis. Reactions were performed on a real-time PCR system (QuantGene 9600, China) following the manufacturer’s recommended cycling conditions using the TaqMan® Real-Time Fluorescent Quantitative PCR Pre-mix (Vazyme, Nanjing, China). For absolute quantification, a sequence-verified recombinant plasmid containing the PoRVA NSP3 gene was used to generate a standard curve; plasmid concentration was converted to copy number and subjected to tenfold serial dilutions (10^1^–10^8^ copies/µL), which were used as RT-qPCR templates. Ct values (y) were regressed against log10 copy number (x) to obtain the equation *y* =  −4.12x + 43.93 (standard error for slope/intercept, 0.02), with *R* =  −0.998 (*R*^2^ > 0.996) and an amplification efficiency of 92.85%, and viral RNA loads in all samples were calculated accordingly. Each sample was tested in technical triplicate.

### Histology and immunohistochemistry

For histopathological assessment of post-infection tissue changes, tissues were fixed in 10% formalin and then transferred to 70% ethanol for further processing. HE staining was performed according to the manufacturer’s protocol (FreeThinking Biotechnology, Nanjing, China). For IHC, fixed sections were incubated with a VP6 monoclonal antibody (1:200) to detect PoRVA antigen. Horseradish peroxidase (HRP)-conjugated goat anti-mouse immunoglobulin (Ig)G (Beyotime, China) served as the secondary antibody. Images were acquired with a digital slide scanner (Ocus 40).

### Statistical analysis

Data were derived from three independent experiments. The statistical analysis plan was not preregistered. All data are presented as mean ± standard deviation (SD). Statistical analyses were performed using Prism 10 (GraphPad Software, Inc., La Jolla, CA, USA). For experiments involving two independent variables (e.g., strain and time), data were analyzed using two-way analysis of variance (ANOVA) followed by Sidak’s multiple comparisons test to assess differences between strains at each individual timepoint (simple effects within rows). For comparisons between two groups at a single timepoint, an unpaired Student’s *t*-test was used. All statistical tests were two-tailed. Statistical significance was indicated by asterisks in the figures (*p < 0.05; **p < 0.01; ***p < 0.001; ****p < 0.0001).

## Results

### Virus isolation and identification

**Figure 1:**
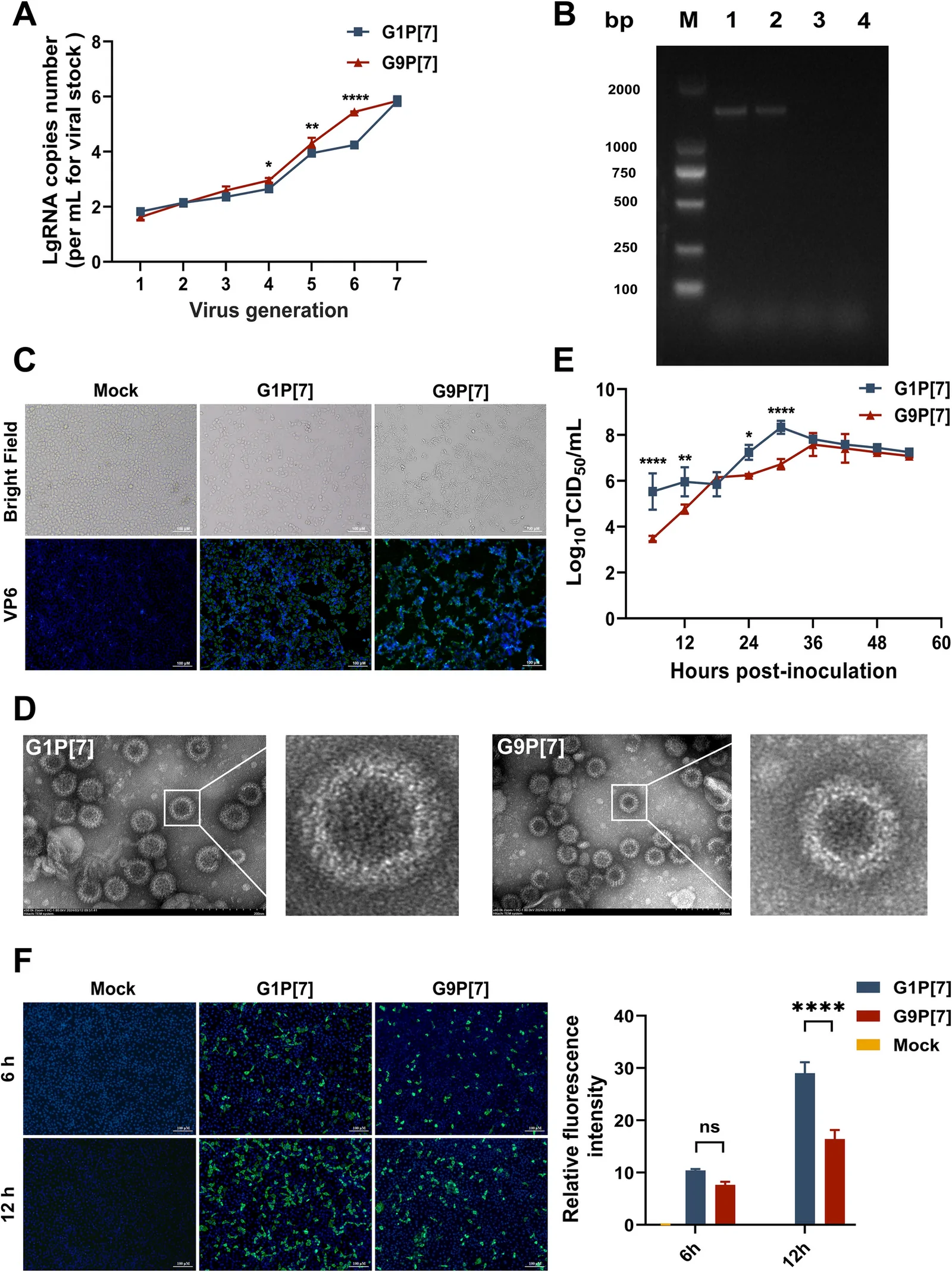
**Identification of PoRVA Isolates XXW2023 and HD2023.** **A** The viral load of PoRVA in the culture supernatants from passages 1 to 7 (P1–P7) was quantified by RT-qPCR. Statistical significance was assessed (*p < 0.05; **p < 0.01; ****p < 0.0001). **B** PCR amplicons from culture supernatants at virus passage 7 (P7) targeting the VP6 gene, with a fragment size of 1356 bp. (M) 2000 bp DNA ladder, (1) Strain XXW2023; (2) Strain HD2023; (3) DMEM containing trypsin (negative control); (4) blank control. **C** CPEs in MA104 cells infected with the seventh-passage (P7) XXW2023 (G9P[7]) or HD2023 (G1P[7]) strains were examined by light microscopy. Viral antigens were detected by indirect IFA. Scale bars =100 μm. **D** Purified PoRVA virions from the culture supernatants of XXW2023 (G9P[7]) and HD2023 (G1P[7]), visualized by transmission electron microscopy (TEM). Scale bars = 200 μm. **E** Growth kinetics of the PoRVA isolates XXW2023 (G9P[7]) and HD2023 (G1P[7]) in MA104 cells (MOI = 0.01). Statistical significance: *p < 0.05; **p < 0.01; ****p < 0.0001. **F** Growth kinetics of PoRVA isolates XXW2023 (G9P[7]) and HD2023 (G1P[7]) in MA104 cells (MOI = 0.01). IFA staining for RVA VP6 (green) with DAPI nuclear counterstain (blue) was performed at 6 and 12 hpi. Scale bars = 100 μm. Fluorescence intensity was quantified. Statistical significance: ns, *p* > 0.05; ****p < 0.0001.

To evaluate replication stability in MA104 cells, the isolates were serially passaged. Viral RNA in culture supernatants collected from passages 1–7 was quantified by NSP3-based RT-qPCR, which showed sustained replication and a progressive increase in viral copy number (Figure 1A). Infectious virus was corroborated at passage 7 by RT-PCR amplification of the VP6 gene (Figure 1B). These analyses confirm stable in vitro propagation of both strains, with viral RNA levels in culture supernatants progressively increasing across successive passages. During passage, typical CPE was evident by 24 h post-infection, including cell rounding, fragmentation, vacuolization, and abundant debris, whereas mock-infected controls showed no cytopathic changes. Indirect immunofluorescence with an anti-VP6 monoclonal antibody confirmed replication of PoRVA strains XXW2023 (G9P[7]) and HD2023 (G1P[7]) in MA104 cells at 12 hpi (Figure 1C). To further assess the virus’s clonal properties, a clonal isolation process was performed. Following three consecutive rounds of plaque purification, a clonal virus isolate was obtained. The clonality was supported by Sanger sequencing of the VP7 gene, which revealed a single, unambiguous nucleotide sequence without evidence of mixed populations. This purified clonal isolate was used for all subsequent passages and experiments.

Transmission electron microscopy (TEM) of purified PoRVA isolates XXW2023 (G9P[7]) and HD2023 (G1P[7]) showed typical virion morphology. Negative-stain TEM revealed wheel-like particles 70–100 nm in diameter with prominent surface spikes typical of rotaviruses (Figure 1D). Virus replication kinetics demonstrated efficient in vitro replication of XXW2023 (G9P[7]) and HD2023 (G1P[7]). Viral titers reached their peak at 36 hpi for XXW2023 and 30 hpi for HD2023, followed by a gradual decline, with maximal titers of 10^7.5^ and 10^8.3^ TCID_50_/mL, respectively (Figure 1E). In parallel, indirect IFA detected viral antigen in MA104 cells at 6 and 12 hpi. Relative to XXW2023, HD2023 produced stronger IFA signals at 12 hpi and a steeper temporal increase (*p* < 0.0001) (Figure 1F).

### Whole-genome typing of pathogenic strains

Whole-genome typing revealed that the VP7-VP4-VP6-VP1-VP2-VP3-NSP1-NSP2-NSP3-NSP4-NSP5 genotype constellation of XXW2023 was G9-P[7]-I5-R1-C1-M1-A8-N1-T1-E1-H1 (Table 2). By the same analysis, the genotype constellation of HD2023 (G1P[7]) was G1-P[7]-I5-R1-C1-M1-A8-N1-T1-E1-H1 (Table 3). All 22 gene segment sequences were deposited in GenBank under accession numbers PQ384413–PQ384423 and PQ384402–PQ384412.

**Table 2:** **Analysis results of each fragment of porcine rotavirus XXW2023 (G9P[7]) strain**

Gene	Genotype	Closest strain	Ident%	Accession
VP7	G9	RVA/Pig-wt/USA/Nebraska33/2010/G9P[X]	99.69	MN862194.1
VP4	P[7]	RVA/Porcine/CHN/HUBEI/2022	99.79	PQ452936.1
VP6	I5	Porcine rotavirus isolate GD-1RV	99.92	OR094869.1
VP1	R1	RVA/Pig-tc/CHN/GDZHF/2023/G9P[7]	99.97	PP235798.1
VP2	C1	RVA/Porcine/CHN/rJXAY01/G5P[23]I12	97.08	PP975111.1
VP3	M1	RVA/Human-wt/CHN/E931/2008/G4P[6]	96.57	KF726038.1
NSP1	A8	RVA/Human-tc/VNM/NT0042/2007/G4P[6]	97.00	LC095894.1
NSP2	N1	RVA/Pig-tc/CHN/GDZHF/2023/G9P[7]	99.90	PP235803.1
NSP3	T1	RVA/Porcine-tc/KOR/174-1/2006/G8P[7]	100	MF940568.1
NSP4	E1	Porcine rotavirus strain NJ2012	100	MT874992.1
NSP5	H1	RVA/Bovine-tc/KOR/KJ11/2006/G8P[7]	99.84	MF940633.1

**Table 3:** **Analysis results of each fragment of porcine rotavirus HD2023 (G1P[7]) strain**

Gene	Genotype	Closest strain	Ident%	Accession
VP7	G1	RVA/DB/DPD/2210243	99.29	OR948019.1
VP4	P[7]	RVA/CHN/CY/GYSX/2022/G9P7I5	99.51	OQ799679.1
VP6	I5	Porcine rotavirus isolate HB-1RV	92.80	OR094874.1
VP1	R1	RVA/Human-wt/CHN/E931/2008/G4P[6]	95.35	KF726036.1
VP2	C1	RVA/Porcine/CHN/rJXAY01/G5P[23]I12	97.08	PP975111.1
VP3	M1	RVA/Human-wt/CHN/E931/2008/G4P[6]	96.57	KF726038.1
NSP1	A8	RVA/Pig-wt/CHN/923E/2021/G9P[23]	95.21	PQ141601.1
NSP2	N1	Rotavirus A strain FX17	98.53	OM362100.1
NSP3	T1	Porcine rotavirus strain YN	96.92	KJ466988.1
NSP4	E1	Porcine rotavirus A isolate LLP48	97.16	KJ126820.1
NSP5	H1	RVA/Pig-wt/BGD/H14020027/G4P[49]	98.15	MK227397.1

Comparative sequence analysis against genotype-matched RVA references identified multiple genome segments in the two PoRVA isolates, most similar to porcine strains. Specifically, the porcine-like segments were VP7, VP4, VP6, VP1, VP2, NSP2, NSP3, and NSP4 in XXW2023 (G9P[7]), and VP7, VP4, VP6, NSP1, NSP2, NSP3, NSP4, and NSP5 in HD2023 (G1P[7]). By contrast, XXW2023 VP3 and NSP1 and HD2023 VP1 and VP3 displayed greater nucleotide sequence similarity to human rotavirus references (Tables 2, 3). Additionally, NSP5 of XXW2023 showed the highest similarity to bovine rotavirus sequences. Whole-genome analysis indicated interspecies reassortment among human, porcine, and bovine strains for XXW2023 (G9P[7]) and between human and porcine strains for HD2023 (G1P[7]).

As presented in Table 4, XXW2023 (G9P[7]) and HD2023 (G1P[7]) are two novel reassortant strains, both possessing a conserved porcine backbone (with the core genes R1-C1-M1 and the nonstructural protein genes N1-T1-E1-H1, which are identical to those of the porcine OSU and human Wa strains) and sharing the P[7]-I5-A8 genotype combination. However, the VP7 gene origins differ: XXW2023 has a G9 genotype distinct from both OSU (G5) and Wa (G1), while HD2023 has a G1 genotype identical to Wa. Specifically, XXW2023’s VP4 (P[7]), VP6 (I5), and NSP1 (A8) are porcine-like, with VP6 (I5) showing similarity to OSU and NSP1 (A8) reflecting typical porcine features. In contrast, HD2023 displays a typical “mixed” reassortant pattern, with VP7 (G1) matching Wa, but VP4 (P[7]), VP6 (I5), and NSP1 (A8) showing porcine traits. These findings clearly indicate that XXW2023 and HD2023 are novel reassortant strains formed through the acquisition of distinct VP7 genes from different sources, while maintaining a porcine backbone.

**Table 4:** **Genotype constellation of porcine, human, and study rotavirus strains**

Strain name	Host	Genotypes										
		VP7	VP4	VP6	VP1	VP2	VP3	NSP1	NSP2	NSP3	NSP4	NSP5
OSU	Pig	G5	P[7]	I5	R1	C1	M1	A1	N1	T1	E1	H1
Wa	Human	G1	P[8]	I1	R1	C1	M1	A1	N1	T1	E1	H1
XXW2023	Pig	G9	P[7]	I5	R1	C1	M1	A8	N1	T1	E1	H1
HD2023	Pig	G1	P[7]	I5	R1	C1	M1	A8	N1	T1	E1	H1

### Whole-genome phylogeny and nucleotide identity define divergent VP4/VP7 lineages in XXW2023 and HD2023

**Figure 2:**
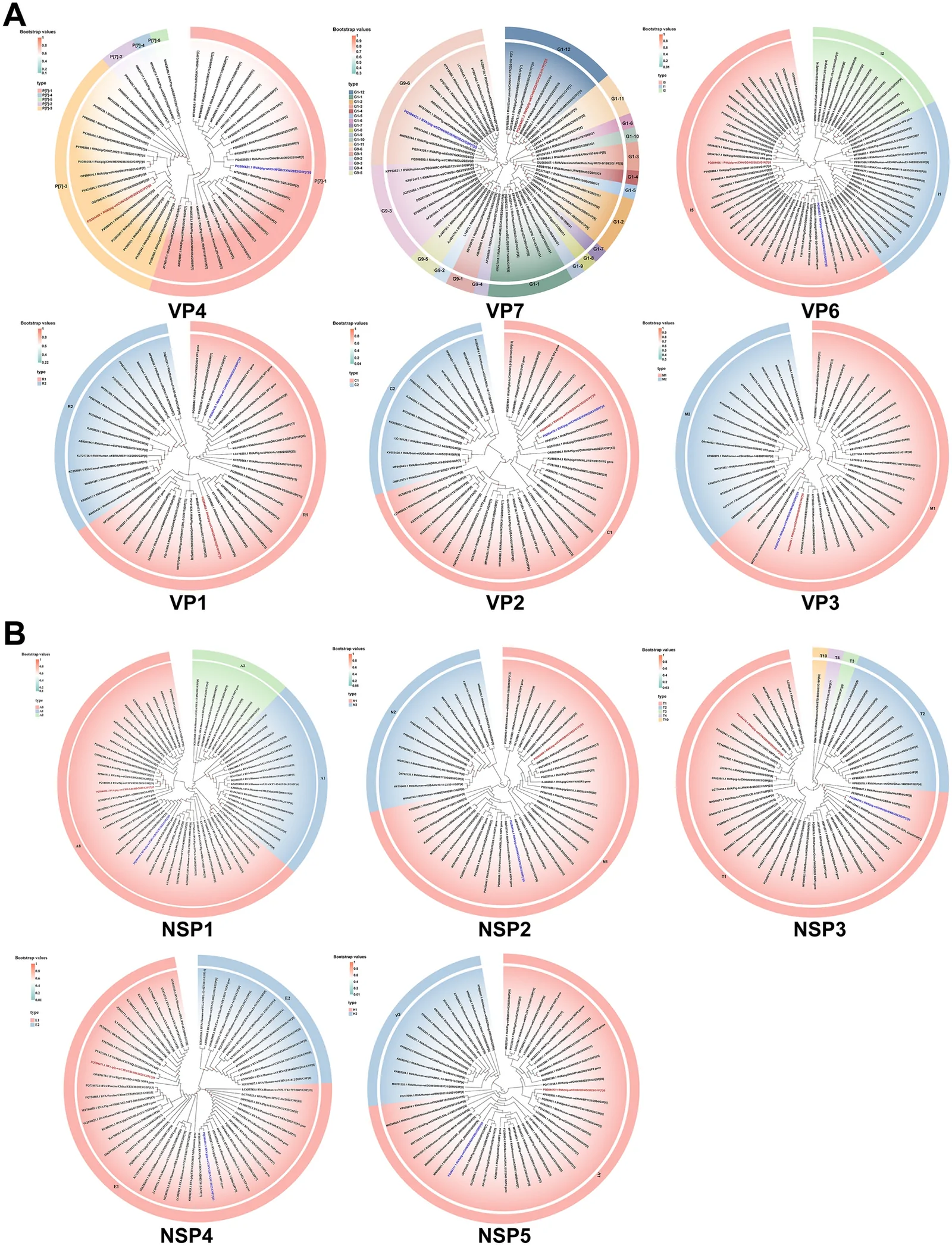
**Phylogenetic analysis of 22 genome segments from representative strains, including XXW2023 (G9P[7]) and HD2023 (G1P[7**]). Neighbor-joining (NJ) trees were inferred in MEGA X (only values above 70% are provided). **A** Phylogenetic trees of the VP4, VP7, VP6, VP1, VP2, and VP3 genes from the PoRVA isolates XXW2023 (G9P[7]) and HD2023 (G1P[7]). Blue dots indicate the XXW2023 (G9P[7]) isolate; red dots indicate the HD2023 (G1P[7]) isolate. **B** Phylogenetic trees of the NSP1, NSP2, NSP3, NSP4, and NSP5 genes from the PoRVA isolates XXW2023 (G9P[7]) and HD2023 (G1P[7]).

**Figure 3:**
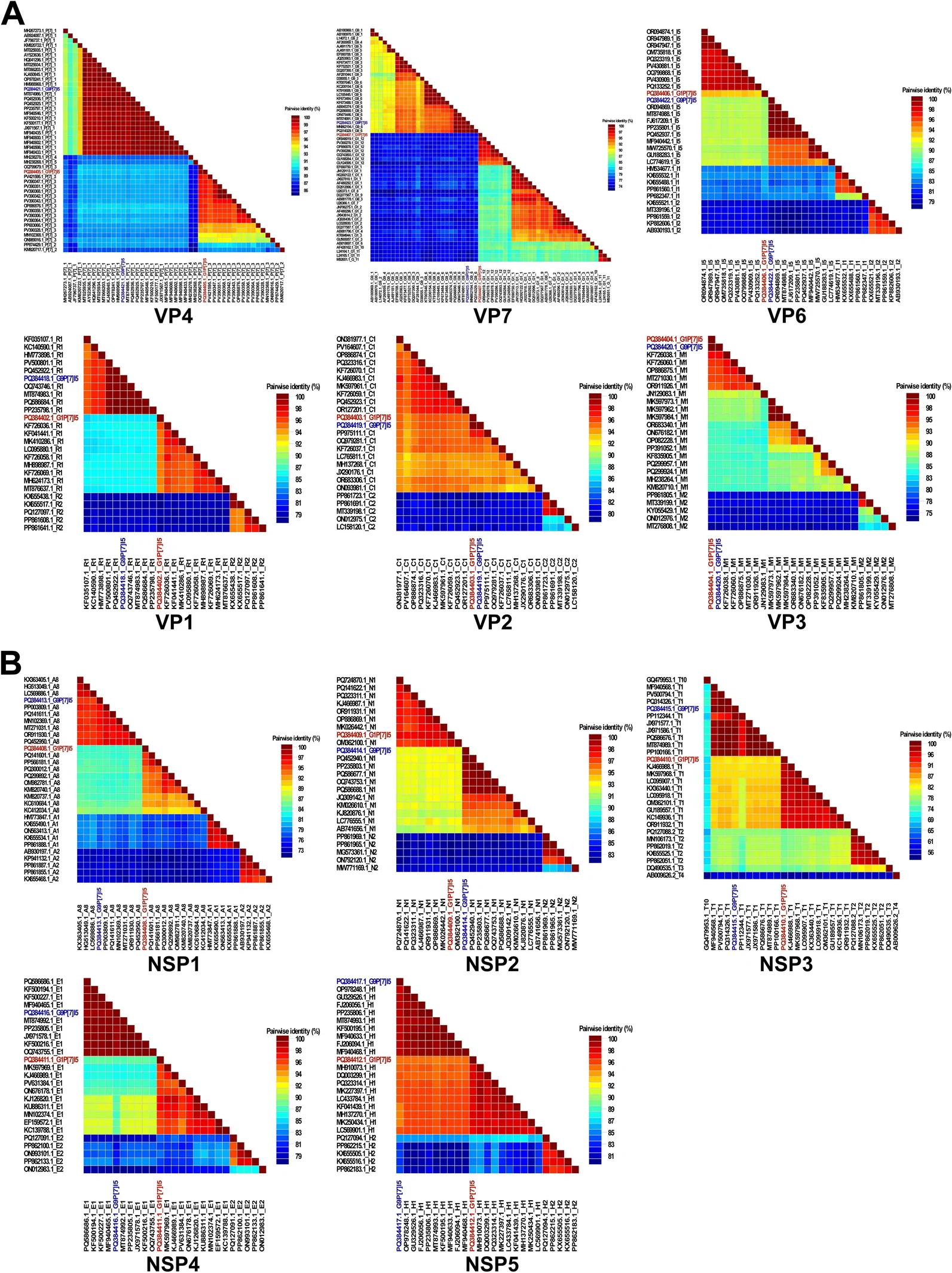
**Nucleotide identity analysis of the 22 genomic segments from representative strains, including XXW2023 (G9P[7]) and HD2023 (G1P[7]).** Pairwise nucleotide identity was calculated with SDT v1.3. **A** VP4, VP7, VP6, VP1, VP2, and VP3 from PoRVA isolates XXW2023 (G9P[7]) and HD2023 (G1P[7]) were compared with representative RVA reference strains. Heatmaps were generated with SDT v1.3 from complete coding sequences. XXW2023 (G9P[7]) is shown in blue text, and HD2023 (G1P[7]) in red text. **B** Pairwise nucleotide identity heatmaps for NSP1-NSP5 of PoRVA isolates XXW2023 (G9P[7]) and HD2023 (G1P[7]) versus representative RVA reference strains, generated with SDT v1.3 from complete coding sequences.

To elucidate the genetic relationships between isolates XXW2023 and HD2023 and other rotaviruses, we analyzed their complete genomes (comprising 11 segments) using nucleotide identity and phylogenetic methods. Sequence alignments and phylogenetic trees (Figures 2A, 3A, Additional file 1) placed XXW2023 within lineage P[7]-1, clustering with the OSU strain and with rotaviruses derived from panda and bovine sources. Within P[7]-1, XXW2023 was most closely related to reference PoRV, including Hubei2022 (PQ452936.1), with a mean nucleotide identity of > 99.5%. HD2023 clustered in lineage P[7]-3, showing the highest nucleotide identity (99.5%) to the 2022 isolate GYSX (OQ799679.1) but only 90.0% identity to the OSU strain. Although both isolates carry P[7] VP4 genes, the nucleotide identity of VP4 between XXW2023 and HD2023 was only 89.9%. Phylogenetic analysis of VP7 (Figures 2A, 3A, Additional file 2) showed that XXW2023 clustered with human strains within lineage G9-6. Its closest relative was the U.S. swine isolate Nebraska33 (MN862194.1), with which it shared 99.7% nucleotide identity. The VP7 gene of HD2023 fell outside the 11 previously defined lineages and clustered with Wa-like strains, corresponding to the newly designated lineage G1–12. HD2023 shared 99.2% nucleotide identity with the Guangdong wild-boar strain DB/DPD/2210243 (OR948019.1). Consistent with their different serotypes, the VP7 genes of XXW2023 and HD2023 shared 76.4% nucleotide identity.

VP6, the inner capsid protein of rotaviruses, is highly conserved across groups. Theuns et al. reported that most porcine isolates belong to genotype I5 [21]. Consistent with this, maximum-likelihood analysis of VP6 (Figure 2A, Additional file 3) placed XXW2023 and HD2023 in genotype I5, but in distinct clades. Phylogenetic analysis revealed that strain XXW2023 (G9P[7]) clustered with porcine, bovine, and human RVAs, while HD2023 (G1P[7]) formed clusters with porcine and dog RVA strains. The overall VP6 nucleotide identity between the two isolates was 89.1%. Furthermore, XXW2023 (G9P[7]) exhibited a high VP6 nucleotide identity (≥ 99.8%) with strain GD-1RV (OR094869.1) (Figure 3A). In contrast, HD2023 (G1P[7]) showed the highest VP6 identity (92.8%) to porcine strain HB-1RV (OR094874.1).

Comparative analysis of VP1 (RNA-dependent RNA polymerase, RdRp) against GenBank references (Figures 2A, 3A, Additional file 4) placed XXW2023 (G9P[7]) and HD2023 (G1P[7]) in distinct clades within lineages containing human and porcine RVAs. XXW2023 (G9P[7]) shared 99.9% VP1 nucleotide identity with porcine isolate GDZHF (PP235798.1), whereas HD2023 (G1P[7]) showed the highest VP1 identity (95.35%) to the human strain E931 (KF726036.1). The VP1 nucleotide identity between XXW2023 and HD2023 was 85.8%. For VP2 and VP3, sequence comparisons (Figures 2A, 3A, Additional files 5 and 6) showed that both XXW2023 and HD2023 shared the highest VP2 nucleotide identity with the porcine isolate rJXAY01 (PP975111.1, 97.1%) and the highest VP3 nucleotide identity to the human strain E931 (KF726038.1, 96.6%).

Phylogenetic analysis of NSP1 (Additional file 7) clustered both XXW2023 and HD2023 within genotype A8, with a pairwise nucleotide identity of 84.1% between them. XXW2023 exhibited the highest sequence identity to a human strain, with the closest being VNM/30378 (HG513049.1; 97.0%), while HD2023 was most closely related to 923E (PQ141601.1; 95.2%). For NSP2-NSP4, both isolates aligned with porcine RVA references and showed high nucleotide identity (Figures 2B, 3B, Additional files 8–10). In contrast, NSP5 (Additional file 11) differed, with XXW2023 grouping with bovine strain KJ11 (MF940633.1; 99.8%) and HD2023 grouping with the Bangladeshi porcine strain H14020027 (MK227397.1; 98.2%).

### XXW2023 and HD2023 recombination analysis

**Figure 4:**
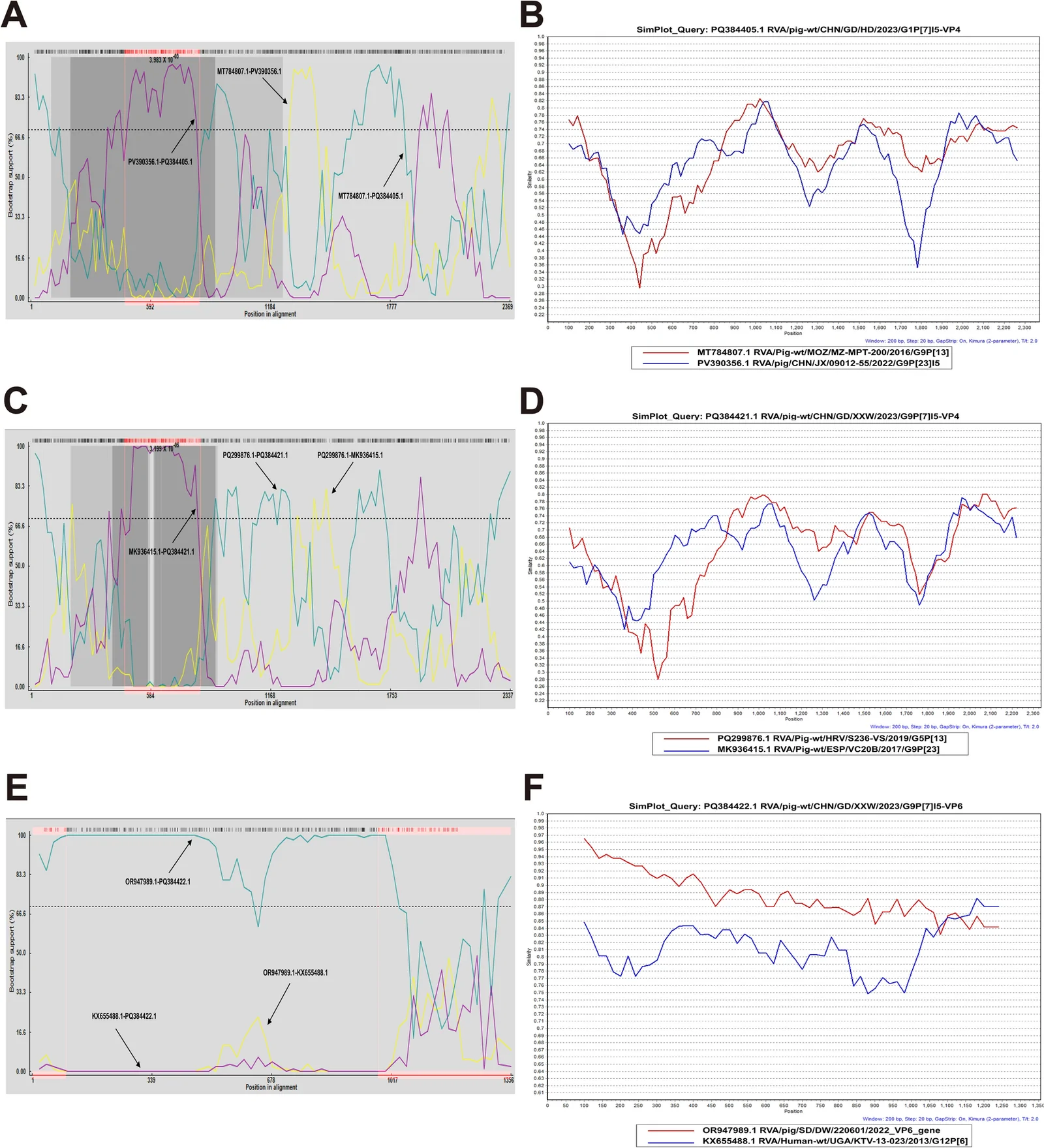
**Cross-over regions in the genomes of XXW2023 and HD2023 identified by RDP4 and SimPlot (red lines denote the major parent and blue lines the minor parent).** **A** RDP4 (Bootscan) showed recombination breakpoints in HD2023 VP4 at nt 458 and 836. **B** The SimPlot analysis was consistent with the RDP4 results. The operating parameters used were the Kimura (two-parameter) distance model, 2.0 Ts/Tv ratio, neighbor-joining tree model, and 1000 bootstrap replicates. **C** Identification of recombination breakpoints (nt 458 and 836) in XXW2023 VP4 via RDP4 Bootscan analysis. **D** Concordance between SimPlot and RDP4 analyses. **E** RDP4 Bootscan analysis was performed to identify recombination breakpoints (nt 1061–1161) in the XXW2023 VP6 gene. **F** SimPlot analysis confirms the RDP4 results.

Genome-wide recombination analysis of all genomic segments from XXW2023 (G9P[7]) and HD2023 (G1P[7]) was performed using RDP v4.101 and validated with SimPlot v3.5.1. Recombination signals were observed only in the VP4 and VP6 segments, while all other segments tested negative. In the VP4 segment, two recombination breakpoints near nt 458 and 836 were identified in both strains. For HD2023, the recombination event was statistically supported (*p* < 0.01) by RDP, Bootscan, MaxChi, Chimaera, and 3Seq, with a Mozambican porcine G9P[13] sequence identified as the major parent and a Chinese porcine G9P[23] sequence as the minor parent (Figure 4A, B). For XXW2023, the recombination was also statistically supported (*p* < 0.01) by RDP, GENECONV, Bootscan, MaxChi, Chimaera, and 3Seq. The VP4 sequence was identified as a recombinant between a Croatian porcine G5P[13] strain (major parent) and a Spanish porcine G9P[23] strain (minor parent) (Figure 4C, D). This pattern aligns with reports that intrasegmental recombination contributes to RVA evolution, particularly in the post-vaccine era [22]. For the VP6 segment, a recombination signal was detected only in XXW2023, with statistical support (*p* < 0.01) from the RDP, MaxChi, and Chimaera algorithms (Figure 4E, F); HD2023 remained negative. The breakpoint region was located at nt 1061–1161. The best-supported parents for the XXW2023 VP6 recombinant were a Ugandan human G12P[6]-I1 sequence (major parent) and a Chinese porcine I5 sequence (minor parent).

The consistent statistical support from multiple independent algorithms for each recombination event strengthens these findings and reduces the likelihood of alignment artifacts. Overall, XXW2023 carries a human–porcine recombinant VP6 segment, supporting the occurrence of interspecies recombination in this strain.

### Comparative analysis of antigenic epitopes in VP4 and VP7 of the isolates

VP8* presents 4 neutralizing epitopes (8-1 to 8-4), and VP5* contains 5 (5-1 to 5-5), encompassing 37 residues. We compared VP4 neutralizing-epitope variation in XXW2023 (G9P[7]) and HD2023 (G1P[7]) with P[7] strains from distinct lineages. As summarized in Tables 5 and 6, substitutions relative to lineage references were concentrated in VP8* epitopes 8-1 (148P → L), 8-2 (180T → A), and 8-3 (114T → A, 116N → S/D, 133T → A/V), and in VP5* epitopes 5-1 (384N → S/D, 393D → H/N/A, 394Y → H, 398T → A, 440T → M/R), 5-3 (459R → K), and 5-5 (306T → A/I). Inter-lineage differences were most pronounced at 8-3 and 5-1. XXW2023 and the rotavirus reference strain OSU fell within lineage 1, whereas HD2023 belongs to lineage 3. Across these three strains, the overall epitope architecture was conserved, and the predominant amino-acid substitutions mapped to epitopes 8-3 (133T → A), 5-1 (384N → S/D; 393D → H), and 5-5 (306T → A). Notably, XXW2023 and OSU differed at epitope 5-1 despite belonging to the same lineage.

**Table 5:** Alignment of the amino acid residues in VP8* antigenic epitopes

	Strain	8-1											8-2		8-3										**8-4**		
		100	146	148	150	188	190	192	193	194	195	196	180	183	113	114	115	116	125	131	132	**133**	**135**		**87**	**88**	**89**
P7-1	PQ384421.1 G9P[7]I5	D	T	P	G	Y	S	T	N	Y	D	T	T	N	Q	T	T	N	Q	E	N	T	Q		T	V	E
	RVA/Pig-wt/CHN/NJ2012/2012/G9P[7]	D	T	P	G	Y	S	T	N	Y	D	T	T	N	Q	T	T	N	Q	E	N	T	Q		T	V	E
	RVA/Pig-tc/USA/1975/OSU/G5P[7]	D	T	P	G	Y	S	T	N	Y	D	T	T	N	Q	T	T	N	Q	E	N	T	Q		T	V	E
	RVA/Pig-tc/BEL/RV277/1977/G1P[7]	D	T	P	G	Y	S	T	N	Y	D	T	T	N	Q	**A**	T	**S**	Q	E	N	T	Q		T	V	E
	RVA/Porcine-tc/KOR/174-1/2006/G8P[7]	D	T	P	G	Y	S	T	N	Y	D	T	T	N	Q	T	T	N	Q	E	N	T	Q		T	V	E
	RVA/Pig-wt/JPN/BU2/2014/G5P[7]	D	T	**L**	G	Y	S	T	N	Y	D	T	T	N	Q	**A**	T	N	Q	E	N	T	Q		T	V	E
P7-2	RVA/Pig-wt/RUS/MR-22/2022/G3P[7]	D	T	**L**	G	Y	S	T	N	Y	D	T	T	N	Q	T	T	**D**	Q	E	N	**A**	Q		T	V	E
	RVA/Pig-wt/BEL/12R005/2012/G4P[7]	D	T	**L**	G	Y	S	T	N	Y	D	T	**A**	N	Q	T	T	N	Q	E	N	T	Q		T	V	E
P7-3	PQ384405.1 G1P[7]I5	D	T	P	G	Y	S	T	N	Y	D	T	T	N	Q	T	T	N	Q	E	N	**A**	Q		T	V	E
	RVA/pig/CHN/HN/07396/2023/G5P[7]I5	D	T	P	G	Y	S	T	N	Y	D	T	T	N	Q	T	T	N	Q	E	N	**A**	Q		T	V	E
	RVA/pig-wt/CHN/CN1P7/2021/G1P[7]	D	T	P	G	Y	S	T	N	Y	D	T	T	N	Q	T	T	N	Q	E	N	T	Q		T	V	E
	RVA/pig/CHN/GZ/05358/2022/G5P[7]I1	D	T	P	G	Y	S	T	N	Y	D	T	T	N	Q	T	T	N	Q	E	N	**V**	Q		T	V	E
	RVA/Pig-wt/CHN/AHBZ2303/2023/G11P[7]	D	T	P	G	Y	S	T	N	Y	D	T	T	N	Q	T	T	N	Q	E	N	**A**	Q		T	V	E
	RVA/pig-wt/GHA/14/2016/G5P[7]	D	T	P	G	Y	S	T	N	Y	D	T	T	N	Q	T	T	N	Q	E	N	**A**	Q		T	V	E
P7-4	RVA/Pig-wt/ESP/F376/2017/G4P[7]	D	T	P	G	Y	S	T	N	Y	D	T	T	N	Q	T	T	N	Q	E	N	T	Q		T	V	E
P7-5	RVA/Pig-wt/ESP/F37/2017/G3P[7]	D	T	P	G	Y	S	T	N	Y	D	T	T	N	Q	T	T	N	Q	E	N	T	Q		T	V	E

Bold letters indicate mutated amino acid residues

**Table 6:** Alignment of the amino acid residues in VP5* antigenic epitopes

	Strain	5-1								5-2	5-3	5-4	5-5
		384	386	388	393	394	398	440	441	434	459	429	306
P7-1	PQ384421.1 G9P[7]I5	**D**	S	A	**H**	Y	T	T	R	E	R	R	**A**
	RVA/Pig-wt/CHN/NJ2012/2012/G9P[7]	**D**	S	A	**H**	Y	T	T	R	E	R	R	**A**
	RVA/Pig-tc/USA/1975/OSU/G5P[7]	**S**	S	A	D	Y	T	T	R	E	R	R	**A**
	RVA/Pig-tc/BEL/RV277/1977/G1P[7]	N	S	A	D	Y	T	T	R	E	R	R	T
	RVA/Porcine-tc/KOR/174-1/2006/G8P[7]	N	S	A	**H**	Y	**A**	T	R	E	R	R	**A**
	RVA/Pig-wt/JPN/BU2/2014/G5P[7]	**S**	S	A	**N**	**H**	T	T	R	E	R	R	T
P7-2	RVA/Pig-wt/RUS/MR-22/2022/G3P[7]	**D**	S	A	D	Y	T	T	R	E	**K**	R	**I**
	RVA/Pig-wt/BEL/12R005/2012/G4P[7]	**D**	S	A	**A**	Y	T	**M**	R	E	R	R	T
P7-3	PQ384405.1 G1P[7]I5	N	S	A	D	Y	T	T	R	E	R	R	T
	RVA/pig/CHN/HN/07396/2023/G5P[7]I5	N	S	A	D	Y	T	T	R	E	R	R	T
	RVA/pig-wt/CHN/CN1P7/2021/G1P[7]	N	S	A	**N**	Y	T	T	R	E	R	R	T
	RVA/pig/CHN/GZ/05358/2022/G5P[7]I1	N	S	A	D	Y	T	T	R	E	R	R	T
	RVA/Pig-wt/CHN/AHBZ2303/2023/G11P[7]	N	S	A	D	Y	T	T	R	E	R	R	T
P7-4	RVA/Pig-wt/ESP/F376/2017/G4P[7]	**S**	S	A	**A**	Y	T	T	R	E	R	R	T
P7-5	RVA/Pig-wt/ESP/F37/2017/G3P[7]	N	S	A	**A**	Y	T	**R**	R	E	R	R	T

Bold letters indicate mutated amino acid residues

In most animal RVA strains, VP4 comprises 776 amino acids, whereas human RVA VP4 lacks a residue near position 135. Accordingly, the human VP8* domain is shorter by one residue than the animal VP8* domain, with the length difference evident at VP8* position [23]. In animal RVAs, trypsin activation sites in VP4 are located at Arg231, Arg241, Arg247, Arg467, and Arg582 [24–26], and an additional putative site occurs at Lys258 [27]. These positions are highly conserved across RVA and were also conserved in isolates XXW2023 (G9P[7]) and HD2023 (G1P[7]) (data not shown).

VP7, a trimeric glycoprotein, harbors two neutralizing epitopes, 7-1 and 7-2. The 7-1 epitope comprises two subdomains, 7-1a and 7-1b, which together encompass 29 amino-acid residues [28]. We compared residues within epitopes 7-1a, 7-1b, and 7-2 in the two isolates against G1 and G9 strains (Table 7). Eight positions (99K, 104Q, 130D, 201Q, 243T, 143K, 148L, 190S) were invariant across G1 and G9 strains. In XXW2023 (G9P[7]) relative to G9 strains from distinct lineages, substitutions were concentrated in 7-1a (87T → A/I, 100D → N/G), 7-1b (212T → K/P), and 7-2 (145D → N, 212S → N/G), whereas residues were relatively conserved within lineages. For HD2023 (G1P[7]), comparison with G1 lineage panels identified 17 conserved residues across 12 lineages and 12 variable positions within epitopes 7-1 and 7-2. HD2023 (G1P[7]) also differed from human vaccine strains Rotarix and RotaTeq-WI79-9 at 7-1a (91T → N, 97D → E) and 7-2 (217M → V). Collectively, 13 distinct amino acid positions within VP7 epitopes 7-1 and 7-2 were found to be substituted in the two isolates.

**Table 7:** **Alignment of the amino acid residues in VP7 antigenic epitopes**

		Strain	7-1a													
			87	91	94	96	97	98	99	100	104	123	125	129	130	291
G9	G9-6	PQ384421.1 G9P[7]I5	T	T	G	T	E	W	K	**N**	Q	D	A	I	D	K
		RVA/Human-wt/JPN/UR14-17/2014/G9P[8]	T	T	G	T	E	W	K	**N**	Q	D	A	I	D	R
		RVA/Pig-wt/USA/Nebraska33/2010/G9P[X]	T	T	G	T	E	W	K	**N**	Q	D	A	I	D	K
	G9-1	RVA/Human/US/WI61/1983/G9P[8]	**A**	T	G	T	E	W	K	D	Q	D	A	I	D	K
		RVA/Human/JPN/F45/1983/G9P[8]	**A**	T	G	T	E	W	K	D	Q	D	A	I	D	K
	G9-2	RVA/Human/India/19863/G9	**I**	T	G	T	E	W	K	G	Q	D	A	I	D	K
	G9-3	RVA/Human-wt/TGO/MRC-DPRU5123/2010/G9P[8]	T	T	G	T	E	W	K	D	Q	D	A	I	D	K
		RVA/Human-wt/CHN/BJ-Q33/2010/G9	T	T	G	T	E	W	K	D	Q	D	A	I	D	K
	G9-4	RVA/Human-wt/CHN/97'SZ37/2000/G9	T	T	G	T	E	W	K	D	Q	D	A	I	D	K
	G9-5	RVA/Human-wt/USA/OM67/2003/G9	T	T	G	T	E	W	K	D	Q	D	A	I	D	K
		RVA/Human-wt/USA/ OM46/2003/G9	T	T	G	T	E	W	K	D	Q	D	A	I	D	K
G1	G1-12	PQ384407.1 G1P[7]I5	T	**N**	N	G	E	W	K	D	Q	S	V	V	D	K
		RVA/pig-wt/CHN/DB/DPD/2022/G1	T	**N**	N	G	E	W	K	D	Q	S	V	V	D	K
		RVA/pig/CHN/sh0902/2009/G1P[7]	T	**N**	N	G	E	W	K	D	Q	S	V	V	D	-
	G1-1	RVA/Human-wt/AUS/CK00083/2008/G1P[8]	T	T	S	G	E	W	K	D	Q	**N**	V	V	D	**R**
		RVA/Human-wt/JPN/ Chi-83/2007/G1	T	T	S	G	E	W	K	D	Q	**N**	V	V	D	**R**
	G1-2	RVA/Human/JPN/OSN9-Rx/2014/G1P[8]	T	T	N	G	E	W	K	D	Q	S	V	V	D	K
		RVA/Human-TC/USA/Rotarix/2009/G1P[8]	T	T	N	G	E	W	K	D	Q	S	V	V	D	K
	G1-3	RVA/Human-wt/USA/Wa/1974/G1P[8]	T	T	N	G	**D**	W	K	D	Q	S	V	V	D	K
		RVA/Vaccine/USA/RotaTeq-WI79-9/1992/G1P7[5]	T	T	N	G	**D**	W	K	D	Q	S	V	V	D	K
	G1-4	RVA/Human/JPN/89H452/2002/G1	T	T	S	G	E	W	K	D	Q	**N**	V	V	D	**R**
	G1-5	RVA/Human/ITA/PA10/90/2006/G1	T	T	N	G	E	W	K	D	Q	S	V	A	D	K
	G1-6	RVA/Human/JPN/AU19/1999/G1	**I**	**N**	N	G	E	W	K	D	Q	S	V	V	D	K
	G1-7	RVA/Human/USA/Ban-59/1996/G1	T	T	S	G	**D**	W	K	D	Q	**N**	V	V	D	**R**
	G1-8	RVA/Human/USA/Egypt-7/1996/G1	T	T	N	G	**D**	W	K	D	Q	S	V	V	D	**R**
	G1-9	RVA/Human/ITA/PA17c/86/2006/G1	T	T	N	G	E	W	K	D	Q	**N**	V	V	D	**R**
	G1-10	RVA/pig/UK/SW20/21/2001/G1	T	**N**	N	G	E	W	K	D	Q	S	V	V	D	K
	G1-11	RVA/Bovine/ARG/T449/G1P[X]	**V**	**N**	N	G	E	W	K	D	Q	**N**	V	V	D	E
		RVA/Pig-tc/VEN/C95/G1P[X]	**V**	**N**	N	G	E	W	K	D	Q	**N**	V	V	D	K

		Strain	7-1b						7-2								
			201	211	212	213	238	243	143	145	146	147	148	190	217	221	264
G9	G9-6	PQ384421.1 G9P[7]I5	Q	N	**K**	A	D	T	K	D	S	T	L	S	E	**N**	G
		RVA/Human-wt/JPN/UR14-17/2014/G9P[8]	Q	N	T	A	D	T	K	D	S	T	L	S	E	S	G
		RVA/Pig-wt/USA/Nebraska33/2010/G9P[X]	Q	N	**K**	A	D	T	K	D	S	T	L	S	E	**N**	G
	G9-1	RVA/Human/US/WI61/1983/G9P[8]	Q	N	T	A	D	T	K	D	S	T	L	S	E	S	G
		RVA/Human/JPN/F45/1983/G9P[8]	Q	N	T	A	D	T	K	D	S	T	L	S	E	S	G
	G9-2	RVA/Human/India/19863/G9	Q	N	T	A	D	T	K	**N**	S	T	L	S	E	**N**	G
	G9-3	RVA/Human-wt/TGO/MRC-DPRU5123/2010/G9P[8]	Q	N	T	A	D	T	K	D	S	T	L	S	E	**G**	G
		RVA/Human-wt/CHN/BJ-Q33/2010/G9	Q	N	T	A	D	T	K	D	S	T	L	S	E	**G**	G
	G9-4	RVA/Human-wt/CHN/97'SZ37/2000/G9	Q	N	**P**	A	D	T	K	D	S	T	L	S	E	S	G
	G9-5	RVA/Human-wt/USA/OM67/2003/G9	Q	N	T	A	D	T	K	D	S	T	L	S	E	S	G
		RVA/Human-wt/USA/ OM46/2003/G9	Q	N	T	A	D	T	K	D	S	T	L	S	E	S	G
G1	G1-12	PQ384407.1 G1P[7]I5	Q	N	V	D	N	T	K	D	Q	N	L	S	**V**	N	G
		RVA/pig-wt/CHN/DB/DPD/2022/G1	Q	N	V	D	N	T	K	D	Q	N	L	S	**V**	N	G
		RVA/pig/CHN/sh0902/2009/G1P[7]	Q	N	V	D	N	T	K	D	Q	N	L	S	**I**	N	-
	G1-1	RVA/Human-wt/AUS/CK00083/2008/G1P[8]	Q	N	V	D	N	T	K	D	Q	N	L	S	**T**	N	G
		RVA/Human-wt/JPN/ Chi-83/2007/G1	Q	N	V	D	N	T	K	D	Q	N	L	S	**I**	N	G
	G1-2	RVA/Human/JPN/OSN9-Rx/2014/G1P[8]	Q	N	V	D	N	T	K	D	Q	N	L	S	M	N	G
		RVA/Human-TC/USA/Rotarix/2009/G1P[8]	Q	N	V	D	N	T	K	D	Q	N	L	S	M	N	G
	G1-3	RVA/Human-wt/USA/Wa/1974/G1P[8]	Q	N	V	D	N	T	K	D	Q	S	L	S	M	N	G
		RVA/Vaccine/USA/RotaTeq-WI79-9/1992/G1P7[5]	Q	N	V	D	N	T	K	D	Q	S	L	S	M	N	G
	G1-4	RVA/Human/JPN/89H452/2002/G1	Q	N	V	D	N	T	K	D	Q	N	L	S	M	N	G
	G1-5	RVA/Human/ITA/PA10/90/2006/G1	Q	N	V	D	N	T	K	D	Q	N	L	S	M	N	G
	G1-6	RVA/Human/JPN/AU19/1999/G1	Q	N	V	D	N	T	K	D	Q	N	L	S	**I**	**D**	G
	G1-7	RVA/Human/USA/Ban-59/1996/G1	Q	N	V	D	N	T	K	D	Q	N	L	S	M	N	G
	G1-8	RVA/Human/USA/Egypt-7/1996/G1	Q	N	V	D	N	T	K	D	Q	N	L	S	**T**	N	G
	G1-9	RVA/Human/ITA/PA17c/86/2006/G1	Q	N	V	D	N	T	K	D	Q	N	L	S	**T**	N	G
	G1-10	RVA/pig/UK/SW20/21/2001/G1	Q	N	V	D	**D**	T	K	D	Q	N	L	S	**V**	N	G
	G1-11	RVA/Bovine/ARG/T449/G1P[X]	Q	**Q**	**C**	**G**	N	T	K	D	Q	N	L	S	**I**	N	G
		RVA/Pig-tc/VEN/C95/G1P[X]	Q	N	V	D	N	T	K	D	Q	N	L	S	**I**	N	G

Bold letters indicate mutated amino acid residues

### Analysis of pathogenicity in suckling mice

**Figure 5:**
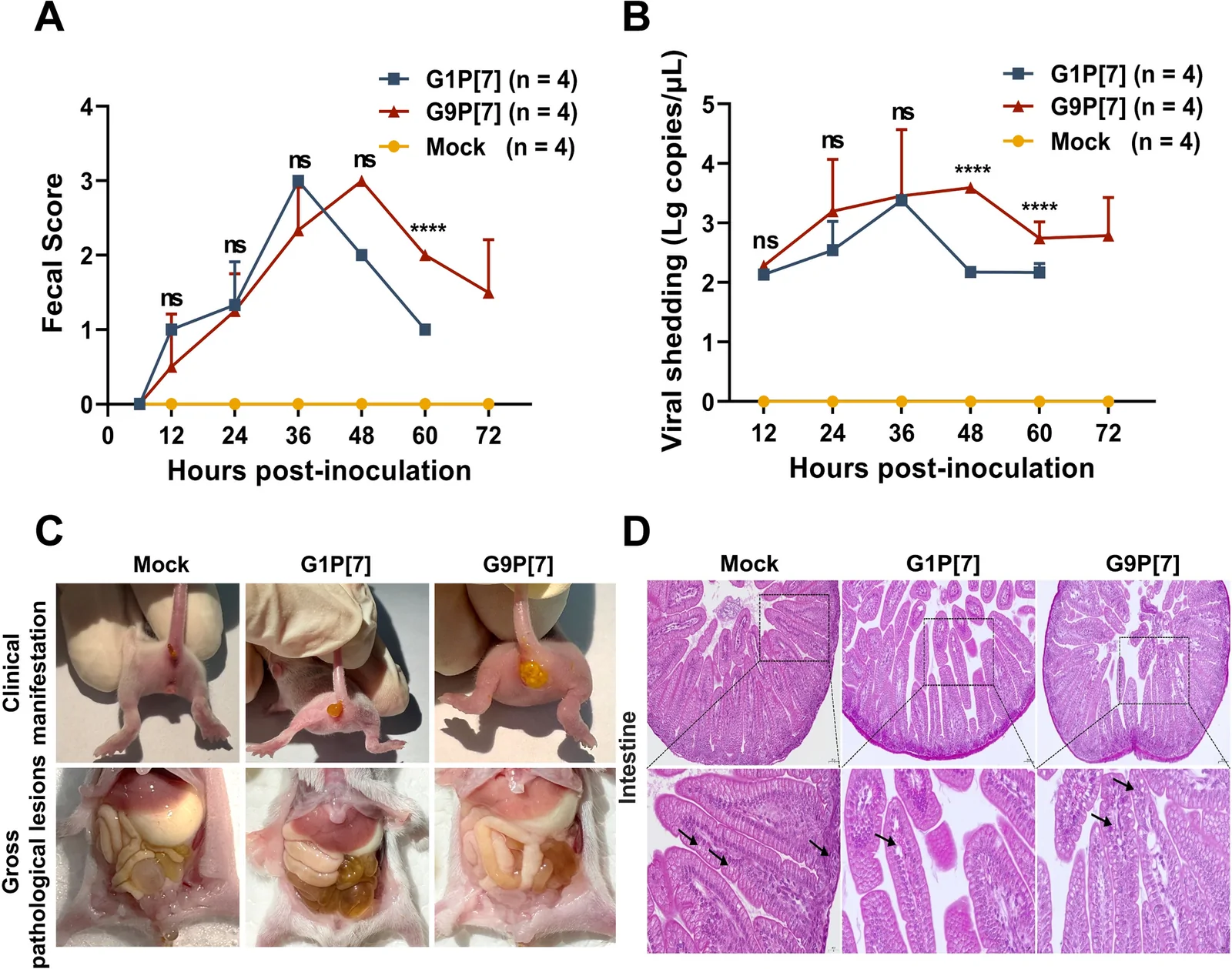
**Pathogenicity of PoRVA strains XXW2023 (G9P[7]) and HD2023 (G1P[7]) in 7-day-old suckling mice.** Pups were orally inoculated with 100 μL of XXW2023, 100 μL of HD2023, or 100 μL of DMEM (control) (*n* = 4 in each group). **A** Diarrhea index in suckling mice was recorded every 12 hpi until necropsy. Data are presented as mean ± SD. Statistical analysis was performed using two-way ANOVA with strain and time as factors, followed by Sidak’s multiple comparisons test. Differences between strains were evaluated at each individual timepoint. Statistical significance: ns, *p* > 0.05; **** *p* < 0.0001. **B** Fecal viral shedding in suckling mice was quantified every 12 hpi until necropsy. Data are presented as mean ± SD. Statistical analysis was performed using two-way ANOVA with strain and time as factors, followed by Sidak’s multiple comparisons test. Differences between strains were evaluated at each individual timepoint. Statistical significance: ns, *p* > 0.05; **** *p* < 0.0001. **C** Clinical signs and gross lesions in challenged versus unchallenged suckling mice. **D** Histopathological lesions in challenged suckling mice. Scale bars = 50 μm or 20 μm.

The 7-day-old BALB/c suckling mice were orally inoculated with either XXW2023 (G9P[7]) or HD2023 (G1P[7]) at 10^7.25^ TCID_50_/mL. Within 24 hpi, diarrhea developed, characterized by yellow watery stools, abdominal distension, intestinal bloating, and perianal erythema; no diarrhea occurred in the control group. For HD2023 (G1P[7]), clinical severity and fecal viral shedding peaked at 36 hpi, and diarrhea began to decline thereafter, with fecal viral loads approaching the assay’s negative threshold by 72 hpi. Peak diarrhea rate and fecal viral shedding for XXW2023 (G9P[7]) were observed at 48 hpi. All mice recovered from diarrhea by 72 hpi; fecal viral shedding was undetectable by 84 hpi (Figure 5A, B). Two suckling mice per group were euthanized at 36 hpi for gross and histopathologic examination. Gross examination (Figure 5C) showed yellow intestinal contents and marked distension of the intestinal wall; no gross lesions were observed in other tissues. Histopathology (Figure 5D) showed no significant lesions in the small intestine of mice infected with XXW2023 (G9P[7]) or HD2023 (G1P[7]) and no evident inflammatory response (black arrows).

### Analysis of pathogenicity of isolates XXW2023 and HD2023 in piglets

**Figure 6:**
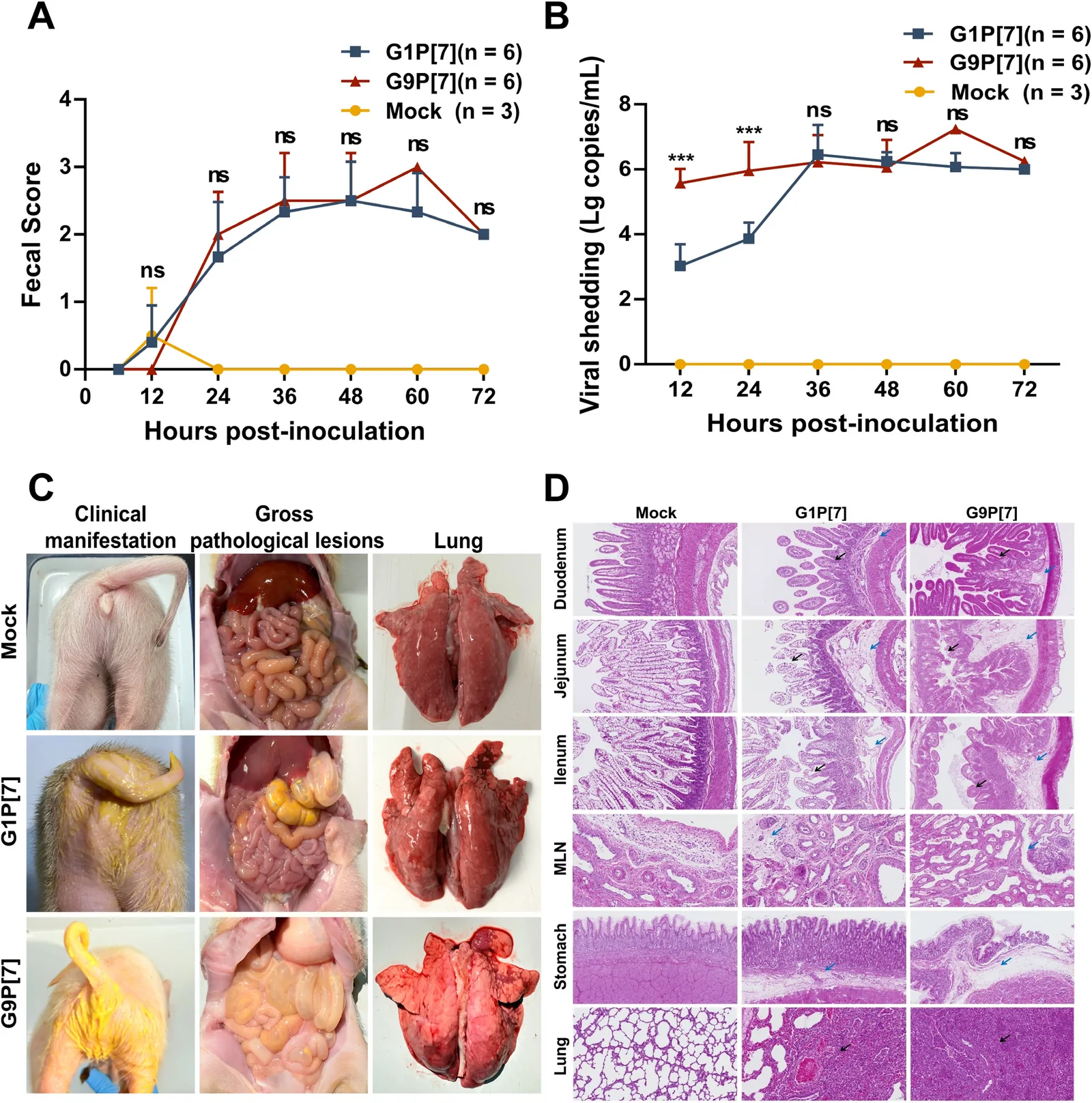
**Pathogenicity of PoRVA strains XXW2023 (G9P[7]) and HD2023 (G1P[7]) in 1-day-old colostrum-deprived piglets. **Colostrum-deprived, 1-day-old piglets were orally inoculated with 4 mL of XXW2023 (G9P[7]) or HD2023 (G1P[7]); controls were orally given 4 mL of DMEM (*n* = 6 in each challenge group; *n* = 3 in the control group). **A** Diarrhea index in piglets was recorded every 12 hpi until necropsy. Data are presented as mean ± SD. Statistical analysis was performed using two-way ANOVA with strain and time as factors, followed by Sidak’s multiple comparisons test. Differences between strains were evaluated at each individual timepoint. Statistical significance: ns, *p* > 0.05. **B** Fecal viral shedding in piglets was quantified every 12 hpi until necropsy. Data are presented as mean ± SD. Statistical analysis was performed using two-way ANOVA with strain and time as factors, followed by Sidak’s multiple comparisons test. Differences between strains were evaluated at each individual timepoint. Statistical significance: ns, *p* > 0.05; *** *p* < 0.001. **C** Clinical signs and gross lesions in challenged versus unchallenged piglets. **D** Histopathological lesions in challenged and unchallenged piglets (black arrows and blue arrows indicate lesions). Scale bars = 50 μm.

Pathogenicity was assessed in 1-day-old piglets orally inoculated with 4 mL of XXW2023 (G9P[7]) or HD2023 (G1P[7]) at 10^7.25^ TCID_50_/mL; mock controls received DMEM. Both challenge groups presented with watery, yellow diarrhea. In the XXW2023 group, diarrhea began in piglet 6 at 24 hpi and involved the remaining cohort by 36 hpi; piglet 3 died at 48 hpi, and signs largely resolved from 60 hpi onward. For the HD2023 group, the onset ranged from 12 to 36 hpi and persisted until 72 hpi; piglet 5 died at 48 hpi, followed by piglets 3 and 4 at 72 hpi. Controls had only transient loose stools without sustained diarrhea or mortality (Figure 6A). RT-qPCR analysis of fecal shedding (Figure 6B) showed that at 12 hpi, PoRVA RNA was already detectable in rectal swabs from piglets infected with XXW2023 (G9P[7]). Shedding increased from 12 to 60 hpi and subsequently declined. For HD2023 (G1P[7]), viral RNA was detectable in 2/3 rectal swabs at 12 hpi, peaked at 36 hpi, and decreased after 48 hpi. These findings indicate that viral replication was established rapidly after infection and maintained at high levels during the acute phase of disease. Statistical comparisons performed at individual timepoints revealed significant differences between strains at early stages of infection, whereas differences were not consistently observed at later timepoints. No viral RNA was detected in control animals.

Observation was limited to 72 hpi due to the rapid clinical progression of infection in this model, as most infected piglets succumbed or reached a moribund state by this timepoint. Gross examination revealed obvious lesions in both infected groups compared with controls (Figure 6C). Key findings included thinning of the intestinal wall with a translucent appearance, more pronounced in piglets infected with XXW2023 (G9P[7]), pale yellow, watery luminal contents, and pulmonary focal hemorrhages, edema, and areas of consolidation; no gross gastric lesions were observed (not shown). Histology further demonstrated lesions of varying severity in the small intestine across both infected groups. In the jejunum and ileum of piglets infected with XXW2023 (G9P[7]) (Figure 6D), the villus epithelium was largely sloughed and shortened, accompanied by marked submucosal edema, mild inflammatory infiltrates, and multifocal vascular dilation (black/blue arrows). Piglets infected with HD2023 (G1P[7]) exhibited a similar pattern, characterized by marked submucosal edema, mild inflammatory infiltrates, and prominent vascular dilation. Extending proximally, only mild submucosal edema was present in the duodenum of the XXW2023 (G9P[7]) group, whereas no significant duodenal lesions were detected in the HD2023 (G1P[7]) group. Consistently, MLN in both infected groups showed interstitial edema with loosely arranged connective tissue (indicated by the blue arrow). Gastric histology was also similar between groups, with submucosal edema, loosely arranged fibrous connective tissue, and minimal inflammatory cell infiltration; however, the lesions were more severe in XXW2023 (G9P[7])-infected piglets (indicated by the blue arrow). Respiratory changes diverged between strains. Pulmonary lesions were more pronounced in the HD2023 (G1P[7]) group, featuring focal parenchymal consolidation, alveolar narrowing to occlusion, and mild diffuse thickening of alveolar walls. These changes were accompanied by widening of the alveolar septa, mild perivascular edema, and abundant inflammatory cell infiltrates (black arrow). In contrast, lungs from XXW2023 (G9P[7])-infected piglets showed extensive parenchymal consolidation with alveolar narrowing to occlusion (black arrow).

**Figure 7:**
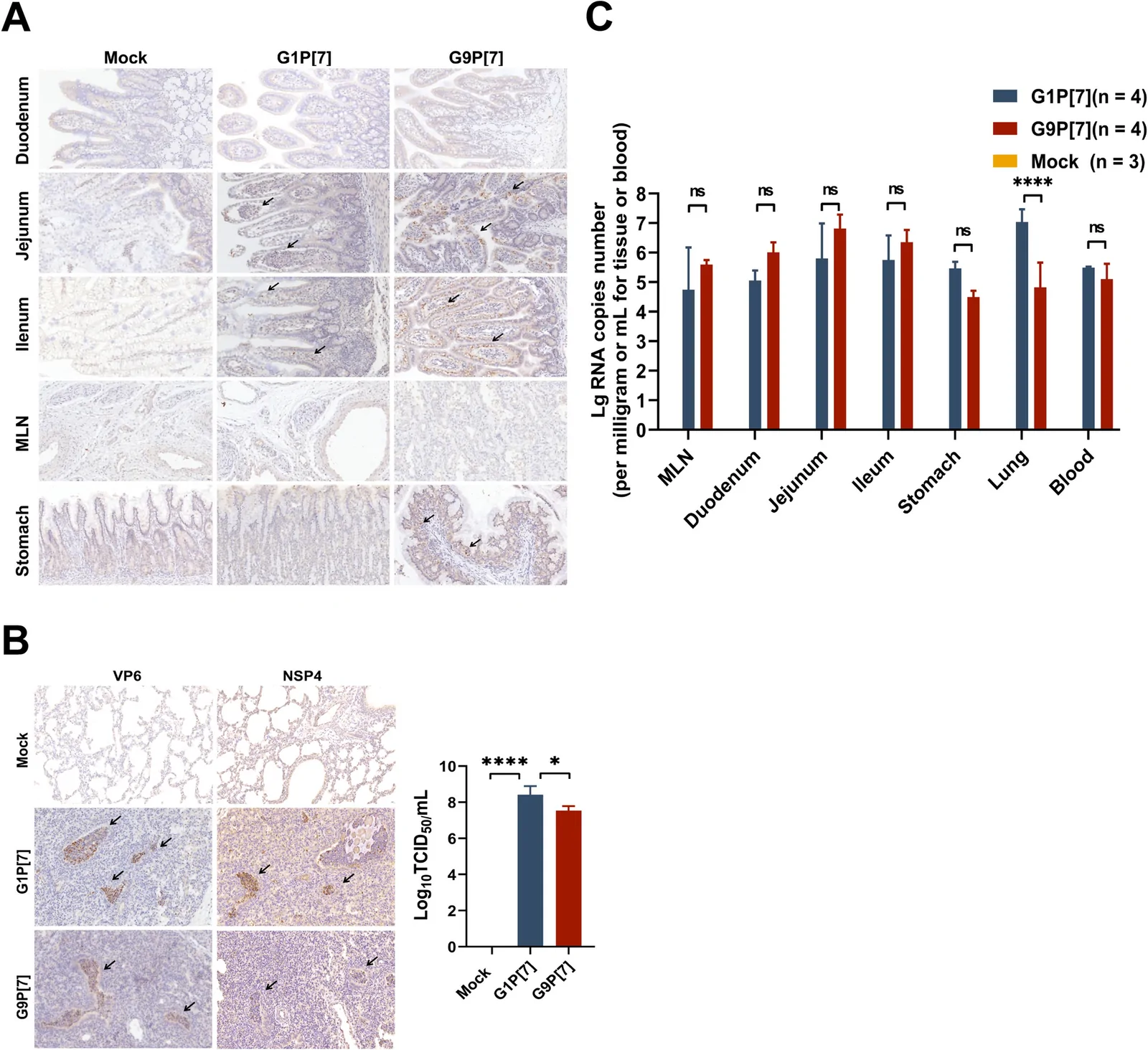
**Characterization of tissue tropism in porcine rotavirus A.** **A** Tissue distribution of PoRVA VP6 antigen in challenged and unchallenged piglets (black arrows indicate VP6 antigen). Scale bars = 20 μm. **B** Detection of active viral replication in the lung. (Left panel) Immunohistochemical staining for the viral nonstructural protein NSP4, a marker of active replication, in lung tissues from challenged piglets. Scale bars = 20 μm. (Right panel) Titers of infectious virus in lung and intestinal (ileum) homogenates, as determined by the TCID_50_ assay on MA104 cells. Data are presented as mean ± SD. Statistical analysis was performed using two-way ANOVA followed by Sidak’s multiple comparisons test. *p < 0.05; ****p < 0.0001. **C** Viral loads in the duodenum, jejunum, ileum, MLN, lung, stomach, and blood were determined at necropsy by RT-qPCR. Data are presented as mean ± SD. Statistical analysis was performed using two-way ANOVA followed by Sidak’s multiple comparisons test. ns, not significant; ****p < 0.0001.

IHC (Figure 7A) detected VP6 antigen in the jejunum and ileum of both PoRVA challenge groups, with no signals observed in the duodenum. The numbers of VP6-positive cells in the small intestine (duodenum, jejunum, ileum) were higher in the XXW2023 (G9P[7]) group compared with the HD2023 (G1P[7]) group. Outside the intestine, VP6 positivity in the XXW2023 group was confined to the stomach, while both strains resulted in VP6 positivity in the lungs (Figure 7A, B). The VP6 signal in the lungs of the HD2023 group was higher than that in the XXW2023 group, notably exceeding the levels observed in the intestines. To determine whether the pulmonary VP6 signal originated from viral replication or passive antigen transfer, two complementary assays were conducted. Initially, immunostaining for the nonstructural protein NSP4 (Figure 7B, left) revealed positive signals in lung tissue, with a pattern and intensity that mirrored strain-dependent differences, consistent with the VP6 findings. Since NSP4 is expressed solely during viral replication, its detection provided direct evidence of viral replication in lung tissue [29]. Subsequently, quantification of infectious virus titers using the TCID_50_ assay (Figure 7B, right) confirmed the presence of high titers of infectious virus in the lungs. The titer in HD2023-infected piglets was significantly higher than that in the XXW2023-infected group (*p* < 0.05), which was consistent with the differences observed in VP6 and NSP4 staining. The detection of both NSP4 protein and infectious virus further confirmed that PoRVA undergoes productive replication in the lungs.

To quantify PoRVA RNA copy numbers (Figure 7C), samples from the duodenum, jejunum, ileum, MLN, stomach, lungs, and blood were collected from piglets in the infected (*n* = 4 initially per group, with two piglets in each infected group succumbing to acute infection before the endpoint; post-mortem blood coagulation precluded collection of qualified serum samples for RNA extraction) and control (*n* = 3) groups. Consequently, for the statistical analysis of viral RNA in serum, the effective sample size was reduced to two per infected group. In XXW2023 (G9P[7])-infected piglets, the highest RNA copy number was found in the jejunum, followed by the ileum, duodenum, and MLN, with viral RNA also detected in the stomach and lungs. In contrast, in the HD2023 (G1P[7])-infected group, the RNA copy number in the lungs was higher than in the small intestine (duodenum, jejunum, ileum) and MLN, exceeding the levels found in the XXW2023-infected group, consistent with the IHC and TCID_50_ results (Figure 7A and B). The stomach also exhibited relatively high RNA levels. Notably, serum from piglets infected with either strain contained PoRVA RNA, indicating significant viremia, which could facilitate systemic viral dissemination.

## Discussion

In recent years, the detection rate of PoRVA in diarrheal cases at large-scale pig farms in China has risen steadily. An earlier epidemiological survey by our team on PoRVA showed widespread circulation in Chinese swine, with G9 and P[7] as the dominant genotypes, and documented frequent interspecies transmission [13]. Despite these recombination events, the full-length VP4 open reading frames of both strains exhibited high nucleotide identities to established P[7] references: XXW2023 showed > 99.5% identity to the porcine P[7] strain OSU (MT025935.1), and HD2023 showed 99.5% identity to the porcine P[7] strain GYSX (OQ799679.1). These values are well above the 80% threshold established by the Rotavirus Classification Working Group (RCWG) for P-genotype assignment [30]. This confirms that, although chimeric in structure, the VP4 genes of these isolates legitimately qualify as genotype P[7] under the standard RCWG criteria, illustrating that intragenic recombination can occur within the framework of a defined genotype without altering its classification. This pattern mirrors that reported for the zoonotic G4P[6] strain, whose NSP3 segment displayed a mixed T1/T7 genotype [31]. The VP6 gene of XXW2023 was a recombinant between human (Ugandan G12P[6]-I1) and porcine (I5) lineages (Figure 4C), underscoring the central role of human–porcine transmission in rotavirus evolution. Consistent with these observations, novel rotavirus strains emerge via interspecies transmission coupled with reassortment and/or intragenic recombination between human and porcine lineages. Such genetic exchange typically occurs among lineages with shared or multiple origins and is a principal driver of RVA evolution in nature. For example, piglets are susceptible to the human Wa strain and develop severe disease after infection [10]. A porcine-origin G9P[23] strain has been isolated from a Thai child with diarrhea [32], and Li et al. also reported a swine G9P[23] strain with a human RVA genetic backbone [33]. Together, these findings show that human and porcine RVA can cross species barriers and frequently reassort and/or recombine, generating novel lineages. Therefore, pigs are key reservoir hosts for zoonotic transmission of rotavirus and the emergence of new genotypes, highlighting the urgent need for targeted, effective prevention and control strategies to limit cross-species RVA transmission and reduce the risk of human spillover.

To date, diversity in RVA antigenic genotypes remains a major constraint on vaccine effectiveness. We mapped neutralizing epitopes in the VP4 and VP7 proteins of both strains and identified substantial substitutions at key sites. Such within-genotype variation may influence host neutralizing antibody titers after infection. In HD2023 (G1P[7]), VP7 harbors mutations at epitopes 7-1a and 7-2 (Table 7) that distinguish it from vaccine strains Rotarix and RotaTeq-WI79-9. These heterologous epitope patterns may reflect selection during interspecies transmission or immune-driven antigenic drift. Therefore, we hypothesize that the identified substitutions could be associated with altered antigenicity and may affect the virus’s recognition by existing neutralizing antibodies. It is crucial to emphasize, however, that this causal relationship remains speculative and must be conclusively validated through direct antigenic analysis, such as virus neutralization assays or antigenic cartography, in future studies. To define the underlying drivers, future work should apply high-resolution antigenic mapping (antigenic cartography) to RVA, a framework successfully used for influenza A viruses and enteroviruses [34, 35].

Across host species, rotavirus infection produces distinct clinical manifestations and variable intestinal and extraintestinal pathology [36, 37]. In this study, both isolates, XXW2023 (G9P[7]) and HD2023 (G1P[7]), caused 60–72 h of diarrhea in 7-day-old suckling mice, and all infected animals shed virus; however, histopathology showed no significant small-intestinal lesions, and no virus RNA was detected in intestinal tissues, suggesting attenuated pathogenicity of porcine rotaviruses in a heterologous host. Consistent with a species-dependent profile, prior studies in piglets have documented robust pathogenicity of multiple PoRV strains, with Kim et al. reporting that PRG942 (G9P[23]) and PRG9121 (G9P[7]) were highly pathogenic in piglets [14]; Wang et al. finding that AHFY2022 (G9P[23]) caused severe diarrhea and intestinal injury in 5- and 27-day-old piglets without mortality [38]; Miao et al. showing that CN127 (G12P[7]) infection induced diarrhea, histopathological changes in intestinal and extraintestinal organs, and viremia, again without mortality [39]; and Wu et al. reporting that, after CN1P7 (G1P[7]) infection, viral RNA was detected in nasal swabs, multiple tissues, and peripheral blood, whereas lung tissue was immunohistochemically negative for RVA antigen and no piglets died [15]. In contrast, our pathogenicity experiments in colostrum-deprived piglets showed marked disease, with both isolates being highly pathogenic and causing severe watery diarrhea and variable intestinal damage; one piglet died in the XXW2023 (G9P[7]) group, whereas three died in the HD2023 (G1P[7]) group at different times post-infection, yielding 50% mortality; moreover, viral RNA was detected in extraintestinal tissues (mesenteric lymph nodes, lungs, stomach) and in blood with either strain (Figure 7C), and marked lesions were present in extraintestinal organs. Strain-specific patterns were evident for XXW2023 (G9P[7]), as mesenteric lymph nodes harbored the second-highest viral load after the small intestine, aligning with prior studies indicating that G9 RVA crosses the intestinal barrier, disseminates systemically via viremia, and replicates in extraintestinal organs, with mesenteric lymph nodes potentially facilitating intestinal escape and supporting prolonged secondary replication before spread to other extraintestinal organs [40]. By contrast, HD2023 (G1P[7]) produced marked pulmonary involvement, with lung injury being pronounced. Critically, our complementary assays provide conclusive evidence for productive viral replication in the lungs, not merely passive antigen transfer. The detection of the nonstructural protein NSP4, expressed only during active replication, offered direct evidence of a complete viral replication cycle in lung tissue [29]. This was further substantiated by the quantification of high infectious virus titers in lung homogenates via TCID_50_ assay. Consistent with this, both viral RNA load and viral antigen (VP6) expression were significantly higher in the lung than in the small intestine (Figure 7A–C). This observation reveals a distinct tissue tropism for this strain, where the lung serves as a primary and productive site of replication. Across multiple readouts (antigen detection, viral RNA, and infectious titer), the pulmonary viral burden for this strain exceeded the intestinal burden at the sampled timepoints, suggesting a distinct tissue distribution and possible pulmonary tropism. To our knowledge, reports describing naturally circulating PoRVA with consistently higher pulmonary than intestinal viral burdens are limited. This pattern differs from that observed for the other strain analyzed here and from the previously reported porcine G1P[7] strain CN1P7, which primarily showed intestinal involvement. This challenges the conventional view that the intestine is always the primary target and highlights the lung as a potentially important and replication-competent extraintestinal site for RVA. In considering the pulmonary tropism, it is noteworthy that efficient pulmonary dissemination of certain rotavirus strains, such as those with the G9P[13] genotype, appears independent of their intestinal replication levels. This suggests that the phenotype may stem from strain-specific interactions with the host. Specifically, it could be due to the diverse glycan receptors present in lung tissue that confer distinct affinity for specific strains [41]. Simultaneously, the viral NSP1 protein may mediate unique innate immune modulation targeted at lung epithelial cells [42]. Variations in VP4/VP7 receptor usage or VP1/VP3 polymerase efficiency may also collectively drive this pulmonary tropism [43–45]. Whether this stems from higher intrinsic replication efficiency in lung epithelial cells requires future direct validation, such as by comparing infection of primary lung versus intestinal epithelial cells in vitro or by titrating infectious virus from tissue homogenates using assays such as cell culture immunofluorescence (CCIF). Taken together, our findings demonstrate that the pulmonary infection pattern, characterized by high-level productive replication, is strain-dependent. Further research is needed to define the precise molecular mechanisms of PoRVA lung infection and its contribution to respiratory disease.

In summary, we isolated two PoRVA strains, designated XXW2023 (G9P[7]) and HD2023 (G1P[7]). Both strains exhibited human-porcine gene reassortment patterns. Specifically, the VP6 gene of XXW2023 was identified as a human–porcine recombinant, while the VP4 genes of both strains were determined to be recombinants originating from porcine P[13] and P[23] lineages. Both strains induced diarrhea in suckling mice and elicited typical clinical symptoms in piglets. Infection with either strain resulted in pathological lesions in both intestinal and extraintestinal tissues of piglets. Notably, following infection with HD2023 (G1P[7]), productive viral replication was confirmed in the lungs, a pattern distinct from previously characterized rotavirus infections. Collectively, these findings enhance our understanding of rotavirus transmission and evolution, provide supporting data for the preliminary hypothesis of “genotype determines phenotype” in rotaviruses, and underscore the need for effective preventive and therapeutic strategies to control these potentially systemic infections.

## Supplementary Information

**Additional file 1. Porcine rotavirus strains used in the evolutionary analysis of the VP4 gene.**

**Additional file 2. Porcine rotavirus strains used in the evolutionary analysis of the VP7 gene.**

**Additional file 3. Porcine rotavirus strains used in the evolutionary analysis of the VP6 gene.**

**Additional file 4. Porcine rotavirus strains used in the evolutionary analysis of the VP1 gene.**

**Additional file 5. Porcine rotavirus strains used in the evolutionary analysis of the VP2 gene.**

**Additional file 6. Porcine rotavirus strains used in the evolutionary analysis of the VP3 gene.**

**Additional file 7. Porcine rotavirus strains used in the evolutionary analysis of the NSP1 gene.**

**Additional file 8. Porcine rotavirus strains used in the evolutionary analysis of the NSP2 gene.**

**Additional file 9. Porcine rotavirus strains used in the evolutionary analysis of the NSP3 gene.**

**Additional file 10. Porcine rotavirus strains used in the evolutionary analysis of the NSP4 gene.**

**Additional file 11. Porcine rotavirus strains used in the evolutionary analysis of the NSP5 gene.**

## Data Availability

Original data of this study are available at Mendeley Data (https://doi.org/10.17632/py8tnjk9pw.1).
