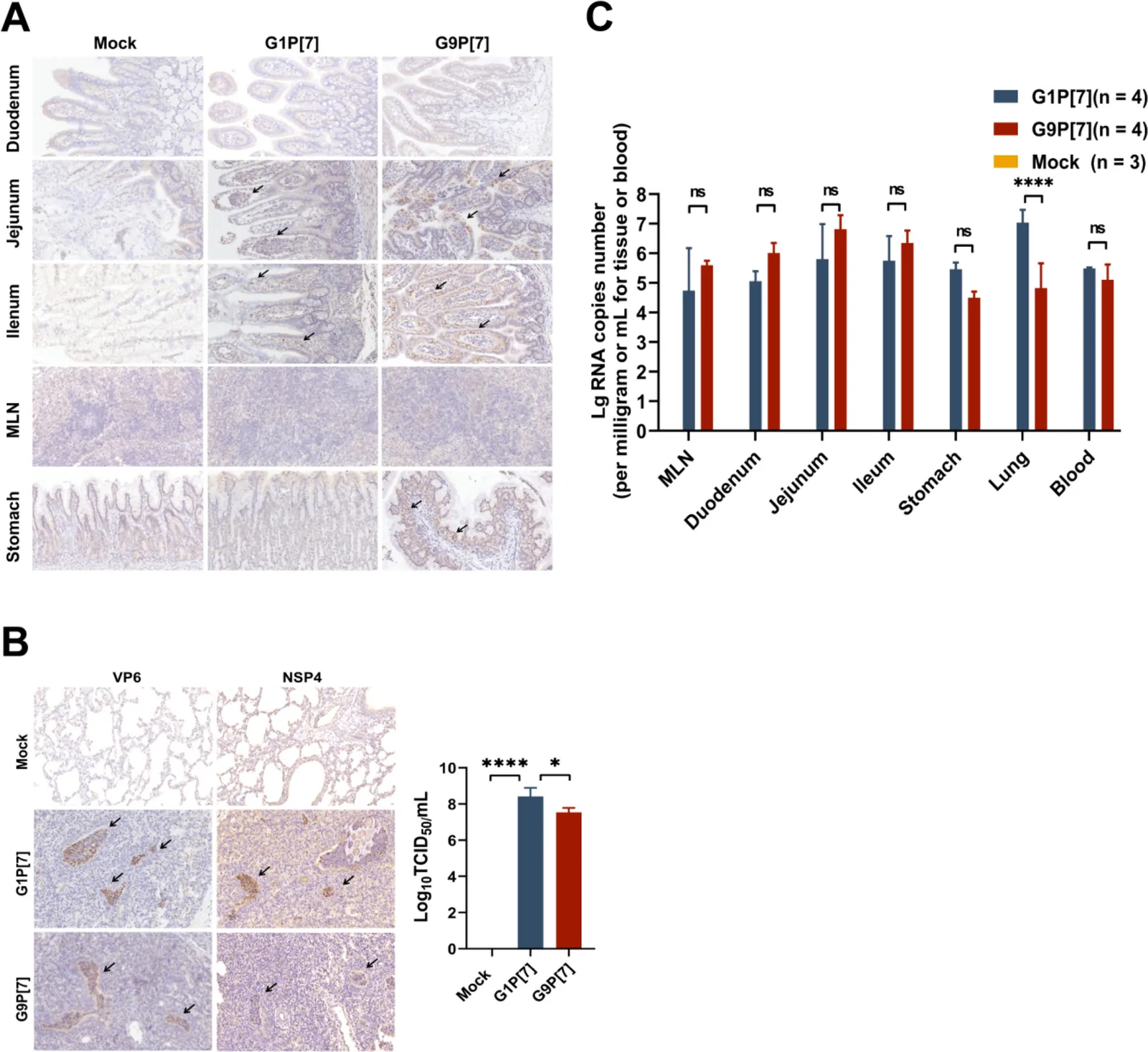# Correction: Whole-genome characterization and pathogenicity of novel human-porcine reassortant rotavirus strains G9P[7] and G1P[7] in China

**DOI:** 10.1186/s13567-026-01861-4

**Published:** 2026-10-20

**Authors:** Meizhen Li, Mengli Qiao, Keshun Bao, Yuanhang Zhang, Panchi Zhang, Jing Chen, Qi Luan, Kun Li, Li Wang, Bin Zhou

**Affiliations:** 1 MOE Joint International Research Laboratory of Animal Health and Food Safety, College of Veterinary Medicine, Nanjing Agricultural University, Nanjing, China; 2 Key Laboratory of Animal Bacteriology, Ministry of Agriculture and Rural Affairs, Nanjing Agricultural University, Nanjing, China; 3 College of Veterinary Medicine, Northeast Agricultural University, Harbin, China; 4 Northeast Science Observation Station for Animal Pathogen Biology, Ministry of Agriculture and Rural Affairs, Harbin, China

**Correction: Veterinary Research (2026) 57:135** **https://doi.org/10.1186/s13567-026-01775-1**

Following publication of the original article [1], the authors identified several inadvertent errors in the published version of the manuscript. These corrections involve textual descriptions and figure presentation. The corrected information is provided below.

1. Corrections to the text

(1) Correction of VP6-positive cell description

In the section “Analysis of pathogenicity of isolates XXW2023 and HD2023 in piglets,” the following sentence was published as:

*“The numbers of VP6-positive cells in the small intestine (duodenum, jejunum, ileum) were higher in the XXW2023 (G9P[7]) group compared with the HD2023 (G1P[7]) group.”*

As stated in the preceding sentence and shown in Fig. 7A, no VP6 antigen signals were observed in the duodenum. Therefore, the sentence should read:

*“The numbers of VP6-positive cells in the small intestine (jejunum and ileum) were higher in the XXW2023 (G9P[7]) group compared with the HD2023 (G1P[7]) group.”*

(2) Correction of amino acid substitution site

In the section describing the comparative analysis of VP7 sequences, the following sentence was published as:

*“In XXW2023 (G9P[7]) relative to G9 strains from distinct lineages, substitutions were concentrated in 7-1a (87T→A/I, 100D→N/G), 7-1b (212T→K/P), and 7-2 (145D→N, 212S→N/G), whereas residues were relatively conserved within lineages.”*

The amino acid substitution site in the 7-2 region was incorrectly described. The sentence should read:

*“In XXW2023 (G9P[7]) relative to G9 strains from distinct lineages, substitutions were concentrated in 7-1a (87T→A/I, 100D→N/G), 7-1b (212T→K/P), and 7-2 (145D→N, 221S→N/G), whereas residues were relatively conserved within lineages.”*

2. Corrections to Figs. 6 and 7

During the preparation of Figs. 6 and 7, the panels corresponding to the mesenteric lymph node (MLN) in the published figures were not the final verified versions intended for publication due to an inadvertent version-selection issue during figure assembly.

The authors have carefully rechecked the original experimental records and prepared revised versions of Figs. 6 and 7, in which the MLN-related panels have been replaced with the corresponding verified panels. The revised figures are provided for review.

Accordingly, the corresponding sentence in the Results section should be revised from:

*“Consistently, MLN in both infected groups showed interstitial edema with loosely arranged connective tissue (indicated by the blue arrow).”*

to:

*“Consistently, the MLNs in both infected groups exhibited mild lymphoid depletion accompanied by focal infiltration of a few eosinophils (indicated by the blue arrow).”*

3. Impact of the corrections

These corrections are limited to the textual descriptions and figure presentation. They do not affect the underlying experimental data, statistical analyses, interpretation, or conclusions of the article.

The incorrect Fig. 6 is:

**Figure:**
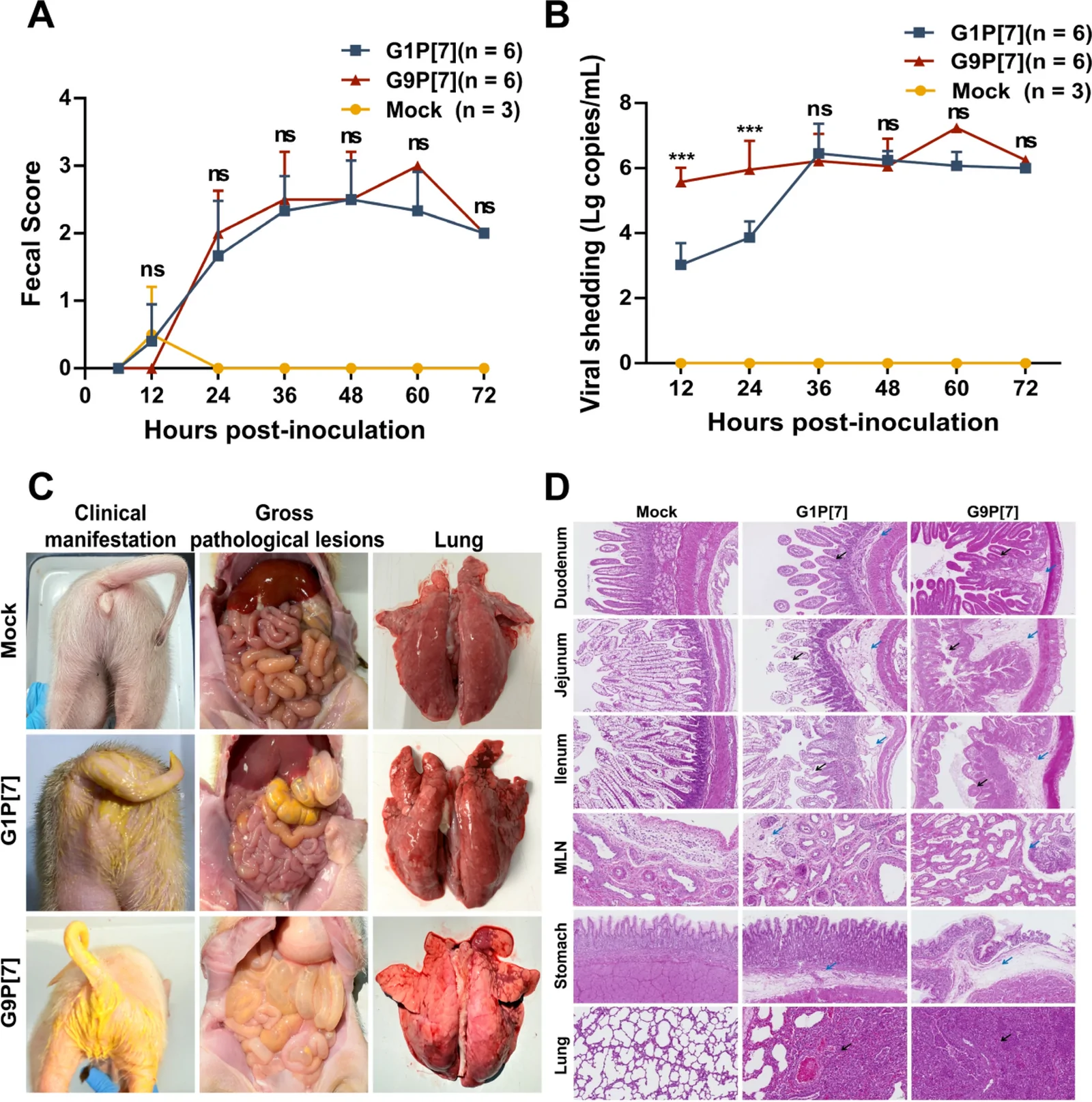

The correct Fig. 6 is:

**Figure:**
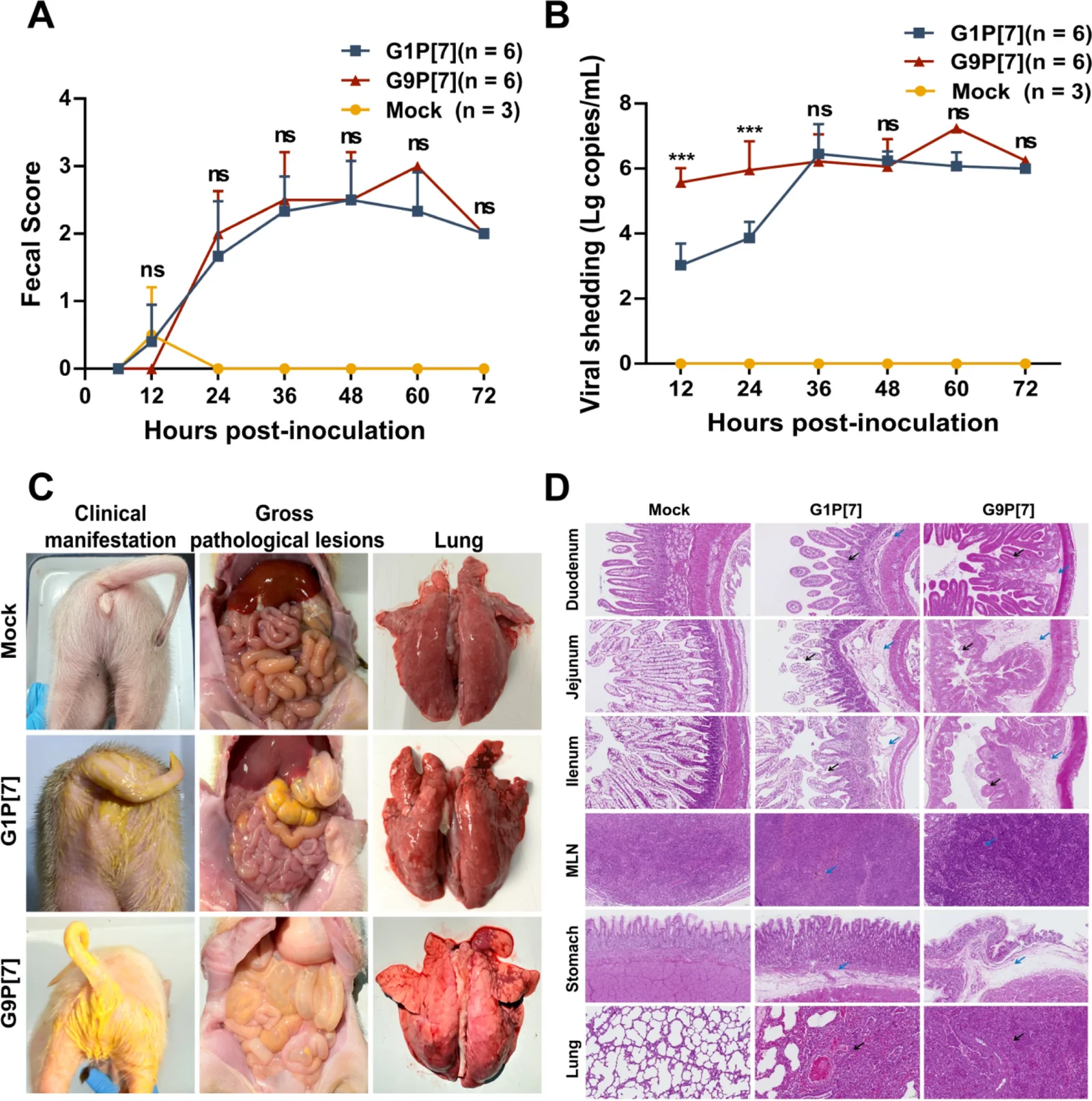

The incorrect Fig. 7 is:

**Figure:**
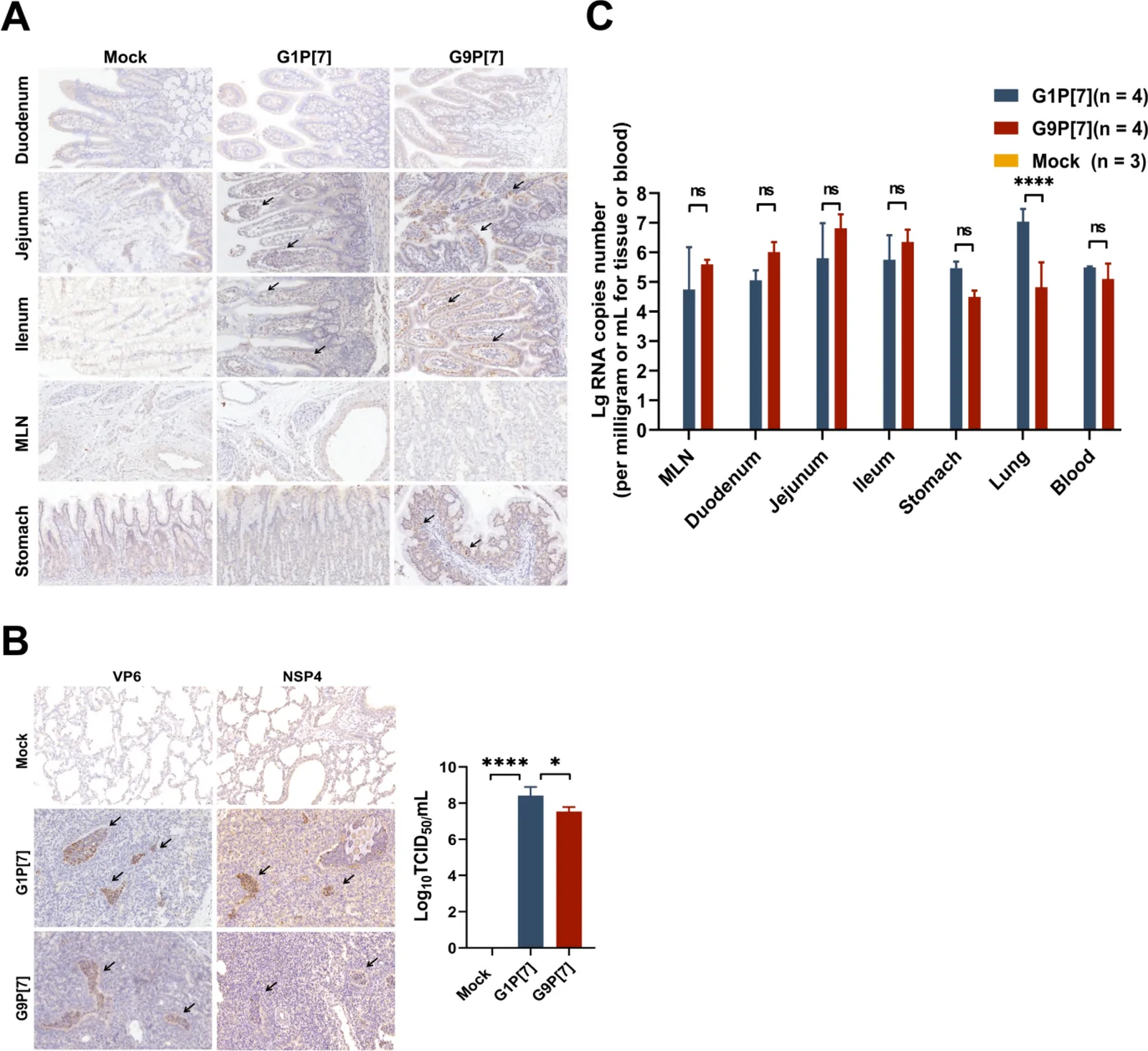

The correct Fig. 7 is:

**Figure:**