# Supplementary material for: Whole-genome characterization and pathogenicity of novel human-porcine reassortant rotavirus strains G9P[7] and G1P[7] in China
Source: Vet Res. 2026 Jul 15;57:135. doi: 10.1186/s13567-026-01775-1 (PMC13371254; doi:10.1186/s13567-026-01775-1)
Supplement: Supplementary file 1 — Additional file 1. Porcine rotavirus strains used in the evolutionary analysis of the VP4 gene. [file 13567_2026_1775_MOESM1_ESM.docx]

Supporting Information for

**Whole-genome characterization and pathogenicity of novel human-porcine reassortant rotavirus strains G9P[7] and G1P[7] in China**

Meizhen Li^1, 2†^, Mengli Qiao^1, 2†^, Keshun Bao^1, 2^, Yuanhang Zhang^1, 2^, Panchi Zhang^1, 2^, Jing Chen^1, 2^, Qi Luan^1, 2^, Kun Li^1, 2^, Li Wang^3, 4*^, Bin Zhou^1, 2, 3, 4*^

^*^Corresponding author. E-mail: [zhoubin@njau.edu.cn](mailto:zhoubin@njau.edu.cn) & wanglicau@163.com

This word file includes:

Tables S1 to S11

Table S1. Porcine rotavirus strains used in the evolutionary analysis of the VP7 gene.

| Accession | Isolate | Collection Date | Geo Location |
| --- | --- | --- | --- |
| OR948019.1 | DB/DPD/2022 | 2022 | China |
| OP886878.1 | CN1P7/2021 | 2021 | China |
| PV390286.1 | HeN/08484/2022 | 2022 | China |
| PV390278.1 | HB/06398/2022 | 2022 | China |
| OQ743893.1 | SD/LCXH03/2022 | 2022 | China |
| EF690750.1 | Agroj23/2002 | 2002 | Bangladesh |
| JN129113.1 | NCA/9J/2010 | 2010 | USA |
| HQ392122.1 | BEL/BE00017/2006 | 2006 | USA |
| JX027818.1 | AUS/CK00083/2008 | 2008 | USA |
| AF480292.1 | Mvd9815/2003 | 2003 | Uruguay |
| DQ512996.1 | Chi-83/2007 | 2007 | Japan |
| AF480296.1 | Mvd9614/2003 | 2003 | Uruguay |
| JX943614.2 | Rotarix/2009 | 2009 | USA |
| JQ926436.1 | se15901-08/2008 | 2008 | Brazil |
| LC028930.1 | OSN9-Rx/2014 | 2014 | Japan |
| KT694944.1 | Wa/1974 | 1974 | USA |
| GU565057.1 | RotaTeq-WI79-9/1992 | 1992 | USA |
| AB081796.1 | 89H452/2002 | 2002 | Japan |
| DQ377587.1 | PA10/90/2006 | 2006 | Italy |
| AB018697.1 | AU19/1999 | 1999 | Japan |
| U26366.1 | Ban-59/1996 | 1996 | USA |
| U26373.1 | Egypt-7/1996 | 1996 | USA |
| DQ377567.1 | PA17c/86/2006 | 2006 | Italy |
| AF426162.1 | SW20/21/2001 | 2001 | UK |
| GU188284.1 | CN1P7/2021 | 2021 | China |
| GU124595.1 | sh0902/2009 | 2009 | China |
| MN862194.1 | Nebraska33/2010 | 2010 | USA |
| PQ314329.1 | YNXD/2024 | 2024 | China |
| OR475446.1 | GD2101/2023 | 2023 | China |
| PQ586690.1 | YNDL/2023 | 2023 | China |
| MT874991.1 | NJ2012/2012 | 2012 | China |
| AB180969.1 | WI61/1983 | 1983 | USA |
| AB180970.1 | F45/1983 | 1983 | Japan |
| EF990708.1 | B3458/2003 | 2003 | Belgium |
| JQ253563.1 | CAU08-463/2008 | 2008 | Korea |
| KF673477.1 | BJ-Q33/2010 | 2010 | China |
| KP752521.1 | TGO/MRC-DPRU5123/2010 | 2010 | Korea |
| D38055.1 | Mc345/2000 | 2000 | Japan |
| AF281044.1 | IECIT-254/2002 | 2002 | Japan |
| DQ207390.1 | 17025-03/2002 | 2002 | Ireland |
| AF260959.1 | 97'SZ37/2000 | 2000 | China |
| AJ491179.1 | OM67/2003 | 2003 | USA |
| AJ491181.1 | USA/ OM46/2003 | 2003 | USA |
| KT007646.1 | CU-B1670/KK/2012 | 2012 | Thailand |
| LC105445.1 | UR14-17/2014 | 2014 | Japan |
| KF673484.1 | BJ-Q1141/2013 | 2013 | China |
| KC200154.1 | SPH0144/2013 | 2013 | China |
| KF673488.1 | BJ-CR7818/2012 | 2012 | China |
| KT919508.1 | VU12-13-101/2013 | 2013 | USA |
| AB045374.1 | K-1/2000 | 2000 | Japan |

Table S2. Porcine rotavirus strains used in the evolutionary analysis of the VP4 gene.

| Accession | Isolate | Collection Date | Geo Location |
| --- | --- | --- | --- |
| OQ799679.1 | CY/GYSX/2022 | 2022 | China |
| MF940600.1 | KJ11/2006 | 2006 | Korea |
| MF940435.1 | K71/2006 | 2006 | Korea |
| MF940602.1 | KJ11/2006 | 2006 | Korea |
| MF940433.1 | K71/2006 | 2006 | Korea |
| MF940598.1 | KJ11/2006 | 2006 | Korea |
| JX971567.1 | K71/2012 | 2012 | Korea |
| KF500210.1 | 174-1/2013 | 2013 | Korea |
| MF940546.1 | 174-1/2006 | 2006 | Korea |
| KF500177.1 | KJ56-1/2013 | 2013 | Korea |
| PP235797.1 | GDZHF/2023 | 2023 | China |
| PQ452936.1 | HUBEI/2022 | 2022 | China |
| PQ452925.1 | SHANXI/2022 | 2022 | China |
| MT874986.1 | NJ2012/2012 | 2012 | China |
| AY523636.1 | JL94/2004 | 2004 | China |
| MT025935.1 | OSU/1975 | 1975 | USA |
| HQ641296.1 | CH-1/2010 | 2010 | China |
| MT025934.1 | 1975/OSU | 1975 | USA |
| KJ450845.1 | OSU-C5111/2010 | 2010 | Spain |
| MT066203.1 | CH/2020/OSU | 2020 | Switzerland |
| OP978241.1 | OSU/1975 | 1975 | USA |
| HM988968.1 | KJ25-1/2008 | 2008 | Korea |
| KM820722.1 | RV277/1977 | 1977 | Italy |
| AB924087.1 | BU2/2014 | 2014 | Japan |
| MH267273.1 | MN9.65a/2008 | 2008 | USA |
| JF796737.1 | PRG9121/2012 | 2012 | Korea |
| MH238278.1 | F376/2017 | 2017 | Spain |
| MH238268.1 | F37/2017 | 2017 | Spain |
| PP874429.1 | MR-22/2022 | 2022 | Russia |
| KM820717.1 | 12R005/2012 | 2012 | Belgium |
| ON989016.1 | CN127/2021 | 2021 | China |
| MN102368.1 | GHA/14/2016 | 2016 | Ghana |
| PV390328.1 | GZ/05358/2022 | 2022 | China |
| PP683066.1 | AHBZ2303/2023 | 2023 | China |
| PV390364.1 | SD/09412/2022 | 2022 | China |
| PV390306.1 | ZJ/02215-1/2022 | 2022 | China |
| PV390358.1 | K71/2006 | 2006 | Korea |
| OP886876.1 | CN1P7/2021 | 2021 | China |
| PV421595.1 | HN/07396/2023 | 2023 | China |
| PV390343. | YN/06318/2022 | 2022 | China |
| PV390347.1 | HB/06398/2022 | 2022 | China |
| PV390351.1 | SX/08194/2022 | 2022 | China |
| PV390342.1 | GD/06308/2022 | 2022 | China |
| PV390368.1 | JS/10277/2022 | 2022 | China |

Table S3. Porcine rotavirus strains used in the evolutionary analysis of the VP3 gene.

| Accession | Isolate | Collection Date | Geo Location |
| --- | --- | --- | --- |
| KF726038.1 | E931/2008 | 2008 | China |
| KF726060.1 | R946/2006 | 2006 | China |
| OP886875.1 | CN1P7/2021 | 2021 | China |
| MT271030.1 | UFS-NGS-MRC-DPRU4723/2014 | 2014 | Zambia |
| OR911926.1 | GD/2022 | 2022 | China |
| JN129083.1 | OL/2010 | 2010 | Nicaragua |
| MK597973.1 | SCLS-3/2018 | 2018 | China |
| MK597962.1 | SCLS-X1/2018 | 2018 | China |
| MK597984.1 | SCLS-R3/2018 | 2018 | China |
| PP391052.1 | BH/2023 | 2023 | China |
| KP752858.1 | MRC-DPRU1562/2008 | 2008 | South Africa |
| KF835905.1 | BP271/2000 | 2000 | Hungary |
| MH238264.1 | F471/2017 | 2017 | Spain |
| OR683351.1 | HBP453/2021 | 2021 | China |
| OR683340.1 | HBP451/2021 | 2021 | China |
| ON676182.1 | SD-1/2021 | 2021 | China |
| PQ299924.1 | L352-K/2019 | 2019 | Croatia |
| OP082228.1 | Iringa-IP057/2019 | 2019 | Tanzania |
| KJ752487.1 | MRC-DPRU1567/2008 | 2008 | South Africa |
| PQ299957.1 | DS76-K/2018 | 2018 | Croatia |
| KM820710.1 | 12R005/2012 | 2012 | Belgium |
| KC139783.1 | LL3354/2000 | 2000 | China |
| HQ641295.1 | CH-1/2008 | 2008 | China |
| MG407647.1 | rj24598/2015 | 2015 | Brazil |
| LC777994.1 | M-HDK9/2021 | 2021 | Japan |
| PQ133250.1 | NG523/2022 | 2022 | China |
| KF726071.1 | R1954/2013 | 2013 | China |
| PQ581887.1 | CH/10.2-20/2022 | 2022 | China |
| MT784806.1 | MZ-MPT-115/2016 | 2016 | Mozambique |
| KF835909.1 | BP1490/1994 | 1994 | Hungary |
| PQ299902.1 | S280-SD/2019 | 2019 | Croatia |
| KF835911.1 | BP1792/2004 | 2004 | Hungary |
| LC095926.1 | NT0205/2007 | 2007 | Viet Nam |
| PQ299968.1 | DS84-Z/2018 | 2018 | Croatia |
| AB924108.1 | BU9/2014 | 2014 | Japan |
| LC765812.1 | RVN17.0271/2017 | 2017 | Viet Nam |
| KT694941.1 | Wa/1974 | 1974 | USA |
| PP861805.1 | Fuzhou23-93/2023 | 2023 | China |
| ON012976.1 | SCMY2/2021 | 2021 | China |
| MT339199.1 | Ph158/1998 | 1998 | USA |
| MT276808.1 | CC425/1998 | 1998 | USA |
| KY055429.1 | BUW-14-085/2014 | 2014 | Uganda |
| KX655519.1 | MUL-13-427/2013 | 2013 | Uganda |
| KX655453.1 | MUL-13-204/2013 | 2013 | Uganda |
| KP883181.1 | Mali-135/2008 | 2008 | Mali |
| KP882675.1 | Ghan-148/2007 | 2007 | Ghana |
| KP882279.1 | Bang-143/2008 | 2008 | Bangladesh |
| AB849002.1 | S120088/2012 | 2012 | Japan |
| KJ721717.1 | MS11142/2005 | 2005 | Brazil |
| OR194482.1 | CHN/22160302/2022 | 2022 | China |
| MH291352.1 | KEN/4009/2017 | 2017 | Kenya |
| MN067446.1 | S19/2012 | 2012 | Morocco |
| KU550277.1 | SS61921417/2015 | 2015 | Spain |

Table S4. Porcine rotavirus strains used in the evolutionary analysis of the VP6 gene.

| Accession | Isolate | Collection Date | Geo Location |
| --- | --- | --- | --- |
| OR094874.1 | HB-1RV/2023 | 2023 | China |
| OR947989.1 | SD/DW/2206011/2022 | 2022 | China |
| OR947947.1 | AH/SS/2210244/2022 | 2022 | China |
| OQ799868.1 | SD/NX5/2022 | 2022 | China |
| PV430909.1 | SX/10352/2023 | 2023 | China |
| OQ799863.1 | LN/TT7/2022 | 2022 | China |
| PV430881.1 | HL/09568/2023 | 2023 | China |
| OM735818.1 | HB-HS77/China/2021 | 2021 | China |
| OQ799845.1 | HeN/TQ2/2022 | 2022 | China |
| PP053570.1 | DB/LC/2310133/2023 | 2023 | China |
| OQ799846.1 | LN/YTW1/2022 | 2022 | China |
| PQ323319.1 | GZ/2023 | 2023 | China |
| PV430921.1 | SC/11028/2023 | 2023 | China |
| PQ133252.1 | NG523/2022 | 2022 | China |
| OQ799816.1 | Shanxi/XT6/2022 | 2022 | China |
| PV390411.1 | GD/06308/2022 | 2022 | China |
| PV390437.1 | JS/10277/2022 | 2022 | China |
| KT820768.1 | SD/JN-1/2014 | 2014 | China |
| PV390420.1 | SX/08194/2022 | 2022 | China |
| OQ799848.1 | LN/YTW2/2022 | 2022 | China |
| PV390419.1 | SD/08083/2022 | 2022 | China |
| PV430886.1 | HE/09659/2023 | 2023 | China |
| MK026439.1 | SCMY-A3/2017 | 2017 | China |
| OQ799856.1 | SD/CY3/2022 | 2022 | China |
| PV390386.1 | GS/05024/2022 | 2022 | China |
| PP683067.1 | AHBZ2303/2023 | 2023 | China |
| PV430833.1 | TJ/07181-4/2023 | 2023 | China |
| MH910067.1 | SCCD-A/2017 | 2017 | China |
| PV430855.1 | SD/08447-57/2023 | 2023 | China |
| OQ799820.1 | CY/LH5/2022 | 2022 | China |
| PV430856.1 | SD/08447-59/2023 | 2023 | China |
| OQ799814.1 | SD/CYFMD2/2022 | 2022 | China |
| KJ752489.1 | ZAF/MRC-DPRU1567/2008 | 2008 | USA |
| OQ799829.1 | CY/LH8/2022 | 2022 | China |
| PQ452926.1 | SHANXI/2022/3.14/E | 2022 | China |
| PV430834.1 | TJ/07181-5/2023 | 2023 | China |
| OQ799831.1 | ZJ/CH1/2022 | 2022 | China |
| ON676184.1 | SD-1/2021 | 2021 | China |
| OQ799830.1 | ZJ/CH2/2022 | 2022 | China |
| OQ799833.1 | ZJ/CH3/2022 | 2022 | China |
| PQ299877.1 | S236-VS/2019 | 2019 | Croatia |
| PQ452948.1 | HUBEI/2022 | 2022 | China |
| JN104614.1 | Mc323/2011 | 2011 | Thailand |
| LC095879.1 | VNM/NT0001/2007 | 2007 | Japan |
| PV430929.1 | HN/11208-39/2023 | 2023 | China |
| OR094869.1 | GD-1RV/2023 | 2023 | China |
| MT874988.1 | NJ2012/2012 | 2012 | China |
| FJ617209.1 | GD/2009 | 2009 | China |
| PP235801.1 | GDZHF/2023 | 2023 | China |
| PQ586691.1 | YNDL/2023 | 2023 | China |
| PQ452937.1 | HUBEI/2022/5.11/u | 2022 | China |
| JF796738.1 | PRG9121/2012 | 2012 | Korea |
| KR052760.1 | LS00006_OSU/1975 | 1975 | USA |
| KJ450847.1 | ESP/OSU-C5111/2010 | 2010 | Spain |
| MF940442.1 | K71/2006 | 2006 | Korea |
| MF940440.1 | K71/2006 | 2006 | Korea |
| EU873010.1 | KV0407/2008 | 2008 | Korea |
| AY538664.1 | JL94/2004 | 2004 | China |
| JX971573.1 | K5/2012 | 2012 | Korea |
| KF500189.1 | KOR/42-1/2006 | 2006 | Korea |
| MF940439.1 | KOR/K71/2006 | 2006 | Korea |
| MF940717.1 | KJ44/2006 | 2006 | Korea |
| MF940552.1 | KOR/174-1/2006 | 2006 | Korea |
| GU188283.1 | CH-1/2009 | 2009 | China |
| MW725570.1 | KNU-GJ2/2020 | 2020 | Korea |
| LC774619.1 | SO1199/2020 | 2020 | Japan |
| MH423866.1 | HJ-2016/2018 | 2018 | China |
| MF940603.1 | KJ11/2006 | 2006 | Korea |
| MH308723.1 | OK.5.68/2008 | 2008 | USA |
| MH267277.1 | MN9.65b/2008 | 2008 | USA |
| KR052739.1 | LS00009_RV0084/2011 | 2011 | USA |
| PV430896.1 | AH/10095/2023 | 2023 | China |
| PV430893.1 | JS/10077-50/2023 | 2023 | China |
| PV430898.1 | HeN/10097-39/2023 | 2023 | China |
| PQ299932.1 | L465-VP/2020 | 2020 | Croatia |
| PV430912.1 | SC/10382/2023 | 2023 | China |
| PV390402.1 | GS/05523/2022 | 2022 | China |
| HM534677.1 | USA/2009727093/2009 | 2009 | USA |
| KX655532.1 | BUW-14-A035/2014 | 2014 | Uganda |
| KX655488.1 | UGA/KTV-13-023/2013 | 2013 | Uganda |
| PP861560.1 | Fuzhou23-140/2023 | 2023 | China |
| PP682347.1 | AHBZ2312/2023 | 2023 | China |
| ON563404.1 | BJ-Q1087/2012 | 2012 | China |
| ON992639.1 | SZ18442196/2018 | 2018 | China |
| ON992632.1 | GD18442035/2018 | 2018 | China |
| ON992619.1 | SC18511086/2018 | 2018 | China |
| ON992594.1 | JL18221381/2018 | 2018 | China |
| ON992565.1 | SZ18442011/2018 | 2018 | China |
| OM037874.1 | HEB16231045/2016 | 2016 | China |
| ON992594.1 | JL18221381/2018 | 2018 | China |
| ON992565.1 | SZ18442011/2018 | 2018 | China |
| OM037874.1 | HEB16231045/2016 | 2016 | China |
| MN106125.1 | E5365/2017 | 2017 | China |
| MG066585.1 | SCLS-2-3/2017 | 2017 | China |
| KU887648.1 | P70/2015 | 2015 | Czech Republic |
| PP585955.1 | HGJM0413/2015 | 2015 | Mozambique |
| MT796883.1 | Wa/1974 | 1974 | USA |
| KT694943.1 | Wa/1974 | 1974 | USA |
| KX655521.1 | MUL-13-427/2013 | 2013 | Uganda |
| PP861559.1 | Fuzhou23-93/2023 | 2023 | China |
| MT339196.1 | Ph158/2020 | 2020 | USA |
| AB930193.1 | AS140023/2014/ | 2014/ | Japan |
| KP882606.1 | Ghan-108/2009 | 2009 | Ghana |
| KP882573.1 | Ghan-105/2009 | 2009 | Ghana |
| KP882276.1 | Bang-143/2008 | 2008 | Bangladesh |
| KJ721704.1 | RJ12225/2006 | 2006 | Brazil |
| MG670599.1 | DOM/3000503705/2014 | 2014 | USA |
| MN067459.1 | S18/2012 | 2012 | Morocco |
| MN067448.1 | S19/2012 | 2012 | Morocco |
| PP848777.1 | HCN1604/2017 | 2017 | Mozambique |
| AB796454.1 | OH3625/2012 | 2012 | Japan |

Table S5. Porcine rotavirus strains used in the evolutionary analysis of the VP2 gene.

| Accession | Isolate | Collection Date | Geo Location |
| --- | --- | --- | --- |
| PP975111.1 | rJXAY01 | 2024 | China |
| KJ466983.1 | YN/2012 | 2012 | China |
| MK597961.1 | SCLS-X1/2018 | 2018 | China |
| PQ323316.1 | GZ/2023 | 2023 | China |
| KF726070.1 | R1954/2013 | 2013 | China |
| OQ979281.1 | AHFY2022/2022 | 2022 | China |
| ON381977.1 | JC3-VP2/2021 | 2021 | China |
| PQ452923.1 | SHANXI/2022/3.14/E | 2022 | China |
| KF726037.1 | E931/2008 | 2008 | China |
| KF726059.1 | R946/2006 | 2006 | China |
| OR127201.1 | KY-2022/2022 | 2022 | China |
| OP886874.1 | CN1P7/2021 | 2021 | China |
| OR683306.1 | HBP442/2021 | 2021 | China |
| LC765811.1 | RVN17.0271/2017 | 2017 | Viet Nam |
| MH137268.1 | SCLSHL-2-3/2017 | 2017 | China |
| PV164607.1 | HB2022/2019 | 2019 | China |
| ON093981.1 | FJSH01/2021 | 2021 | China |
| JX290176.1 | TM-a/2009 | 2009 | China |
| MF462322.1 | LNCY/2016 | 2016 | China |
| MH697614.1 | TM-a-P1/2018 | 2018 | China |
| KU886314.1 | HLJ/15/1/2015 | 2015 | China |
| MH697647.1 | TM-a-P60/2018 | 2018 | China |
| KC579565.1 | DC1476/1974 | 1974 | USA |
| MW575218.1 | HeNNY-01/2019 | 2019 | China |
| KC579949.1 | DC1230/1980 | 1980 | USA |
| JF781159.1 | NMTL/2008 | 2008 | China |
| JX416205.1 | M37/1982 | 1982 | Venezuela |
| KR052758.1 | LS00006_OSU/1975 | 1975 | USA |
| KC580110.1 | DC581/1979 | 1979 | USA |
| MT066201.1 | 2020/OSU | 2020 | China |
| KC579631.1 | DC570/1979 | 1979 | USA |
| PQ452934.1 | HUBEI/2022/5.11/u | 2022 | China |
| KF500175.1 | KJ56-1/2004 | 2004 | Korea |
| KJ450843.1 | OSU-C5111/2010 | 2010 | Spain |
| KJ659486.1 | LS00003 | 2015 | USA |
| KC580492.1 | DC1260/1980 | 1980 | USA |
| PV500800.1 | YNKM/2023 | 2023 | China |
| GU199515.1 | OSU/1975 | 1975 | USA |
| KJ659476.1 | LS00004 | 2015 | USA |
| KC580527.1 | DC273/1979 | 1979 | USA |
| JX971581.1 | K71/2006 | 2006 | Korea |
| PQ586694.1 | YNDL/2023 | 2023 | China |
| JX971570.1 | K5/2004 | 2004 | Korea |
| KC580121.1 | DC1210/1980 | 1980 | USA |
| MT874984.1 | NJ2012/2012 | 2012 | China |
| KC579554.1 | DC2314/1976 | 1976 | USA |
| KC580424.1 | DC527/1979 | 1979 | USA |
| KC580336.1 | DC576/1979 | 1979 | USA |
| KC580283.1 | DC1127/1977 | 1977 | USA |
| MF940591.1 | KJ11/2006 | 2006 | Korea |
| MF940589.1 | KJ11/2006 | 2006 | Korea |
| JQ309139.1 | H-1/1975 | 1975 | UK |
| HM773954.1 | DC827/1978 | 1978 | USA |
| MF940590.1 | KJ11/2006 | 2006 | Korea |
| MF940424.1 | K71/2006 | 2006 | Korea |
| MF940425.1 | K71/2006 | 2006 | Korea |
| KC579736.1 | DC1212/1980 | 1980 | USA |
| MF940588.1 | KJ11/2006 | 2006 | Korea |
| LC438922.1 | RRV_NB1215_31/1987 | 1987 | Venezuela |
| LC438921.1 | RRV_NB1215_30/1987 | 1987 | Venezuela |
| KT694940.1 | Wa/1974 | 1974 | USA |
| PP861723.1 | Fuzhou23-93/2023 | 2023 | China |
| PP861691.1 | Pingtan21-4/2021 | 2021 | China |
| ON012975.1 | SCMY2/2021 | 2021 | China |
| MT339198.1 | Ph158/1998 | 1998 | USA |
| LC158120.1 | LUS12-14/2012 | 2012 | Zambia |
| KY055428.1 | BUW-14-085/2014 | 2014 | Uganda |
| KX655507.1 | MSK-13-048/2013 | 2013 | Uganda |
| KX655474.1 | MUL-12-117/2012 | 2012 | Uganda |
| KJ940064.1 | SC19868/2011 | 2011 | Brazil |
| OR194469.1 | CHN/22160307/2022 | 2022 | China |
| MH291368.1 | KEN/3920/2017 | 2017 | Kenya |
| KY497552.1 | PAK93/2010 | 2010 | Pakistan |
| MF940643.1 | KJ19-2/2006 | 2006 | Korea |

Table S6. Porcine rotavirus strains used in the evolutionary analysis of the VP1 gene.

| Accession | Isolate | Collection Date | Geo Location |
| --- | --- | --- | --- |
| KF726036.1 | E931/2008 | 2008 | China |
| KF041441.1 | GX54/2013 | 2013 | China |
| KF726058.1 | R946/2006 | 2006 | China |
| MK410286.1 | SWU-1C/2018 | 2018 | China |
| LC095880.1 | NT0001/2007 | 2007 | Viet Nam |
| MH898987.1 | SCJY-5/2017 | 2017 | Bangladesh |
| KF726069.1 | R1954/2013 | 2013 | China |
| MH624173.1 | SC11/2017 | 2017 | China |
| MT876637.1 | Moscow-1P/2015 | 2015 | Russia |
| PQ141626.1 | 923X/2021 | 2021 | China |
| PQ141606.1 | 923E/2021 | 2021 | China |
| LC569891.1 | PK2015-1-0001 | 2015 | Thailand |
| MH137269.1 | SCLSHL-2-3/2017 | 2017 | China |
| MH697624.1 | TM-a-P20/2018 | 2018 | China |
| OP082201.1 | Morogoro-RP074/2019 | 2019 | Tanzania |
| PP384139.1 | QT/2023 | 2023 | China |
| GU189551.1 | R479/2009 | 2009 | China |
| OP082196.1 | Iringa-IP058/2019 | 2019 | Tanzania |
| LC095902.1 | NT0073/2007 | 2007 | Viet Nam |
| OR756386.1 | VE5852/2020 | 2020 | Viet Nam |
| LC095935.1 | NT0599/2008 | 2008 | Viet Nam |
| PQ586684.1 | YNXD/2023 | 2023 | China |
| PP235798.1 | GDZHF/2023 | 2023 | China |
| OQ743746.1 | YN-A/2021 | 2021 | China |
| MT874983.1 | NJ2012/2012 | 2012 | China |
| PQ452922.1 | SHANXI/2022/3.14/E | 2022 | China |
| PV500801.1 | YNKM/2023 | 2023 | China |
| KC140590.1 | CAU12-2/2012 | 2012 | Korea |
| HM773898.1 | DC4608/1980 | 1980 | USA |
| KF035107.1 | BRB/2012821133/2012 | 2012 | Barbados |
| JN129055.1 | OL/2010 | 2010 | Nicaragua |
| OP886873.1 | CN1P7/2021 | 2021 | China |
| ON736960.1 | SLV/3000645819/2016 | 2016 | El Salvador |
| KR052725.1 | LS00010_RV00146/2012 | 2012 | USA |
| OR683305.1 | HBP442/2021 | 2021 | China |
| KC579564.1 | DC1476/1974 | 1974 | USA |
| KC580005.1 | DC102/1974 | 1974 | USA |
| OR683327.1 | HBP478/2021 | 2021 | China |
| KC579553.1 | DC2314/1976 | 1976 | USA |
| LC776551.1 | N-Fu1/2022 | 2022 | Japan |
| OR683316.1 | HBP445/2021 | 2021 | China |
| PQ300009.1 | DS404-VS/2020 | 2020 | Croatia |
| GU199514.1 | OSU/1975 | 1975 | USA |
| KT694939.1 | Wa/1974 | 1974 | USA |
| KX655438.1 | MUL-13-171/2013 | 2013 | Uganda |
| PQ127097.1 | IRN/502312/2021 | 2021 | Iran |
| PP861641.1 | Fuzhou23-93/2023 | 2023 | China |
| PP861608.1 | Pingtan21-2/2021 | 2021 | China |
| KX655517.1 | MUL-13-427/2013 | 2013 | Uganda |
| AB930194.1 | S140023/2014 | 2014 | Japan |
| KJ721726.1 | MS11142/2005 | 2005 | Brazil |
| DQ205221.1 | ITA/30-96/1996 | 1996 | Italy |
| MH291387.1 | KEN/3994/2017 | 2017 | Kenya |
| MN067444.1 | S19/2012 | 2012 | Morocco |
| MH291383.1 | KEN/3946/2017 | 2017 | Kenya |
| MF940638.1 | KJ19-2/2006 | 2006 | Korea |
| KU356640.1 | M292/2013 | 2013 | Bangladesh |
| KJ639023.1 | S13-45/2013 | 2013 | Japan |
| KC257091.1 | MRC-DPRU447/2002 | 2002 | Sudan |

Table S7. Porcine rotavirus strains used in the evolutionary analysis of the NSP1 gene.

| Accession | Isolate | Collection Date | Geo Location |
| --- | --- | --- | --- |
| PQ141601.1 | 923E/2021 | 2021 | China |
| PP566181.1 | GDFZ/2023 | 2023 | China |
| PQ300012.1 | DS404-VS/2020 | 2020 | Croatia |
| PQ299892.1 | S244-VS/2019 | 2019 | Croatia |
| OM982781.1 | S20-0073/2020 | 2020 | Switzerland |
| KM820740.1 | 12R022/2012 | 2012 | Belgium |
| KM820737.1 | 12R002/2012 | 2012 | Belgium |
| KC412034.1 | Arg4671/2006 | 2006 | Argentina |
| KJ412559.1 | 1809SR/2009 | 2009 | Paraguay |
| KF835938.1 | BP1125/2004 | 2004 | Hungary |
| OM982780.1 | S20-0073/2020 | 2020 | Switzerland |
| PQ299980.1 | DS229-Z/2020 | 2020 | Croatia |
| PQ299829.1 | S55-VS/2018 | 2018 | Croatia |
| PQ299947.1 | C48-VS/2020 | 2020 | Croatia |
| KR052741.1 | LS00007_Gottfried/1975 | 1975 | USA |
| KM820739.1 | 12R006/2012 | 2012 | Belgium |
| KF835937.1 | BP271/2000 | 2000 | Hungary |
| OR192581.1 | Rota_1a/2017 | 2017 | Chile |
| KJ659437.1 | LS00008/1975 | 1975 | USA |
| JQ993324.1 | BE2001/2009 | 2009 | Belgium |
| OM982707.1 | SS3/2020 | 2020 | Switzerland |
| AB741655.1 | Ryukyu-1120/2011 | 2011 | Japan |
| PQ300001.1 | DS327-Z/2020 | 2020 | Croatia |
| PQ299903.1 | S280-SD/2019 | 2019 | Croatia |
| KM820742.1 | 12R046/2012 | 2012 | Belgium |
| PP874432.1 | MR-22/2022 | 2022 | Russia |
| LC777995.1 | M-HDK9/2021 | 2021 | Japan |
| LC776496.1 | K-Br29/2021 | 2021 | Japan |
| KX988270.1 | KYE-14-A047/2014 | 2014 | Uganda |
| MH308721.1 | OK.5.68a/2008 | 2008 | USA |
| KR052720.1 | LS00010_RV00146/2012 | 2012 | USA |
| FJ154080.1 | mcs/10-07/2007 | 2007 | India |
| OM982730.1 | S19-1115/2019 | 2019 | Switzerland |
| KM820738.1 | 12R005/2012 | 2012 | Belgium |
| MH238095.1 | F456/2017 | 2017 | Spain |
| PP003809.1 | GL/2022 | 2022 | China |
| MN102369.1 | 14/2016 | 2016 | Ghana |
| PQ141611.1 | 923H/2021 | 2021 | China |
| MT271031.1 | UFS-NGS-MRC-DPRU4723 | 2014 | Zambia |
| OR911930.1 | GD/2022 | 2022 | China |
| PQ452950.1 | HUBEI/2022 | 2022 | China |
| KX363405.1 | VNM/14226_39 | 2012 | Viet Nam |
| HG513049.1 | VNM/30378/2009 | 2009 | Viet Nam |
| LC569886.1 | DU2014-259/2014 | 2014 | Thailand |
| LC569897.1 | PK2015-1-0001 | 2015 | Thailand |
| LC776543.1 | A-Ta1/2022 | 2022 | Japan |
| KX363336.1 | VNM/14150_53 | 2012 | Viet Nam |
| OR756389.1 | VE5852/2020 | 2020 | Viet Nam |
| KY937198.1 | CC9192/2014 | 2014 | Cambodia |
| JN104623.1 | Mc345/2011 | 2011 | Japan |
| KF726039.1 | E931/2008 | 2008 | China |
| MT784833.1 | MZ-MPT-198/2016 | 2016 | Mozambique |
| PV026147.1 | RHeN2/2021 | 2021 | China |
| LC433780.1 | TK1797/2007 | 2007 | Nepal |
| MK227393.1 | H14020027 | 2014 | Bangladesh |
| MG781058.1 | CMP-011-09/2009 | 2009 | Thailand |
| LC208014.1 | 07N1760/2007 | 2007 | Nepal |
| JQ309141.1 | H-1/1975 | 1975 | UK |
| KF726072.1 | R1954/2013 | 2013 | China |
| KP753056.1 | MRC-DPRU3878/2008 | 2008 | South Africa |
| MH137274.1 | SCLSHL-2-3/2017 | 2017 | China |
| LC776554.1 | N-Fu1/2022 | 2022 | Japan |
| KY053149.1 | KNA/08979/2015 | 2015 | SKN |
| HM773847.1 | 2007719907/2007 | 2007 | USA |
| KX655534.1 | BUW-14-A035/2014 | 2014 | Uganda |
| KX655490.1 | KTV-13-023/2013 | 2013 | Uganda |
| PP861888.1 | Fuzhou23-140/2023 | 2023 | China |
| ON563413.1 | BJ-Q1087/2012 | 2012 | China |
| ON992526.1 | SZ18442205/2018 | 2018 | China |
| ON992518.1 | GD18442038/2018 | 2018 | China |
| ON992487.1 | SD18370064/2018 | 2018 | China |
| OM037851.1 | GX16451181/2016 | 2016 | China |
| MN106155.1 | E6356/2019 | 2019 | China |
| KT694945.1 | Wa/1974 | 1974 | USA |
| KP883050.1 | Mali-050/2008 | 2008 | MLI |
| OQ440165.1 | D230-ZG/2019 | 2019 | Croatia |
| KX632249.1 | NSA-13-043/2013 | 2013 | Uganda |
| MF940724.1 | KJ44/2006 | 2006 | South Korea |
| DQ146655.1 | Dhaka25/2002 | 2002 | Bangladesh |
| AB930197.1 | S140023/2014 | 2014 | Japan |
| KX655468.1 | MUL-13-496/2013 | 2013 | Uganda |
| PP861855.1 | Pingtan21-4/2021 | 2021 | China |
| PP861887.1 | Fuzhou23-93/2023 | 2023 | China |
| KP941132.1 | Keny-061/2008 | 2008 | Kenya |
| KP882687.1 | Ghan-149/2008 | 2008 | Ghana |
| KP882192.1 | Bang-114/2008 | 2008 | Bangladesh |
| OR194488.1 | 22160303/2022 | 2022 | China |

Table S8. Porcine rotavirus strains used in the evolutionary analysis of the NSP2 gene.

| Accession | Isolate | Collection Date | Geo Location |
| --- | --- | --- | --- |
| OM362100.1 | FX17/2021 | 2021 | China |
| MH910070.1 | SCCD-A/2017 | 2017 | China |
| OR911931.1 | GD/2022 | 2022 | China |
| OP886869.1 | CN1P7/2021 | 2021 | China |
| KJ466987.1 | YN/2012 | 2012 | China |
| MK026442.1 | SCMY-A3/2017 | 2017 | China |
| PQ724870.1 | ZT2130/2023 | 2023 | China |
| PQ323311.1 | GZ/2023 | 2023 | China |
| PQ141622.1 | 923X/2021 | 2021 | China |
| PP025952.1 | 05E/2023 | 2023 | China |
| PV631382.1 | SQ-23/2023 | 2023 | China |
| MT271032.1 | UFS-NGS-MRC-DPRU4723/2014 | 2014 | Zambia |
| PP566192.1 | S3CF/2023 | 2023 | China |
| PP003810.1 | GL/2022 | 2022 | China |
| PQ581889.1 | 10.2-20/2022 | 2022 | China |
| OM982753.1 | S18-1463/2018 | 2018 | Switzerland |
| KC020033.1 | Nov10-N806/2010 | 2010 | Russia |
| PQ299992.1 | DS306-OB/2020 | 2020 | Croatia |
| KC610685.1 | 2CR/2009 | 2009 | Italy |
| PQ299981.1 | DS229-Z/2020 | 2020 | Croatia |
| PQ299915.1 | L54-SM/2018 | 2018 | Croatia |
| KP752760.1 | MRC-DPRU1576/2007 | 2007 | South Africa |
| PP235803.1 | GDZHF/2023 | 2023 | China |
| MT874990.1 | NJ2012/2012 | 2012 | China |
| LC776485.1 | I-TP2/2021 | 2021 | Japan |
| MK272884.1 | COD055/1991 | 1991 | Brazil |
| KC155681.1 | Nov10-N459/2010 | 2010 | Russia |
| OQ743753.1 | YN-A/2021 | 2021 | China |
| PQ586677.1 | YNXD/2023 | 2023 | China |
| PQ586688.1 | YNDL/2023 | 2023 | China |
| PQ452940.1 | HUBEI/2022/5.11/u | 2022 | China |
| JQ309142.1 | H-1/1975 | 1975 | UK |
| LC776555.1 | N-Fu1/2022 | 2022 | Japan |
| KM026610.1 | ba36627-88/1988 | 1988 | Brazil |
| KJ820876.1 | R70/1997 | 1997 | Brazil |
| AB741656.1 | Ryukyu-1120/2011 | 2011 | Japan |
| KJ482279.1 | ROTA06/2013 | 2013 | Brazil |
| KJ752478.1 | MRC-DPRU1567/2008 | 2008 | South Africa |
| PP874433.1 | MR-22/2022 | 2022 | Russia |
| KC155672.1 | Nov05-394/2005 | 2005 | Russia |
| PP669372.1 | UFS-BOC001/2018 | 2018 | South Africa |
| MT292016.1 | COD00155/1991 | 1991 | Brazil |
| GU189556.1 | R479/2004 | 2004 | China |
| MH238121.1 | F473/2017 | 2017 | Spain |
| KC020027.1 | O202/2007 | 2007 | Russia |
| OR192609.1 | Rota_2b/2017 | 2017 | Chile |
| KT694946.1 | Wa/1974 | 1974 | USA |

Table S9. Porcine rotavirus strains used in the evolutionary analysis of the NSP3 gene.

| Accession | Isolate | Collection Date | Geo Location |
| --- | --- | --- | --- |
| KJ466988.1 | YN/2012 | 2012 | China |
| MK597968.1 | SCLS-X1/2018 | 2018 | China |
| KX363440.1 | VNM/14250_9/2012 | 2012 | Viet Nam |
| LC095907.1 | NT0073/2007 | 2007 | Viet Nam |
| OM362101.1 | FX17/2021 | 2021 | China |
| LC095918.1 | NT0077/2007 | 2007 | Viet Nam |
| GU189557.1 | R479/2004 | 2004 | Japan |
| KC149936.1 | LL36755/2003 | 2003 | China |
| OR911932.1 | GD/2022 | 2022 | China |
| MK410291.1 | SWU-1C/2018 | 2018 | China |
| PP025951.1 | CHHeN/05E/2023 | 2023 | China |
| JX290174.1 | TM-a/2009 | 2009 | China |
| PQ452941.1 | HUBEI/2022/5.11/u | 2022 | China |
| LC776498.1 | K-Br29/2021 | 2021 | Japan |
| MH910071.1 | SCCD-A/2017 | 2017 | China |
| KJ482317.1 | ROTA21/2013 | 2013 | Brazil |
| OM982785.1 | CHE/S20-0073/2020 | 2020 | Switzerland |
| KF723312.1 | 5BS/2009 | 2009 | Italy |
| PQ299993.1 | DS306-OB/2020 | 2020 | Croatia |
| OM982709.1 | SS3/2020 | 2020 | Switzerland |
| KR632628.1 | ME848/12/2012 | 2012 | Italy |
| OM982732.1 | S19-1115/2019 | 2019 | Switzerland |
| PQ299982.1 | DS229-Z/2020 | 2020 | Croatia |
| PQ299916.1 | L54-SM/2018 | 2018 | Croatia |
| OQ440167.1 | D230-ZG/2019 | 2019 | Croatia |
| AB779644.1 | CMP45/08/2008 | 2008 | Thailand |
| OQ440193.1 | D572-ZG/2021 | 2021 | Croatia |
| PQ300014.1 | DS404-VS/2020 | 2020 | Croatia |
| JN129013.1 | NCA/OL/2010 | 2010 | Nicaragua |
| MH238143.1 | F456/2017 | 2017 | Spain |
| PV500794.1 | YNKM/2023 | 2023 | China |
| JX971586.1 | K71/2006 | 2006 | Korea |
| MF940568.1 | KOR/174-1/2006 | 2006 | Korea |
| PP112344.1 | OSU-NSP3-2A-3xFL-Una | 2023 | USA |
| PQ314326.1 | YNXD/2024 | 2024 | China |
| JX971577.1 | K5/2004 | 2004 | Korea |
| PQ586676.1 | YNXD/2023 | 2023 | China |
| MT874989.1 | NJ2012/2012 | 2012 | China |
| PP100166.1 | JSNJ2019/2019 | 2019 | China |
| KF500182.1 | KJ56-1/2004 | 2004 | Korea |
| GU329525.1 | CH-1/2009 | 2009 | China |
| MF940458.1 | K71/2006 | 2006 | Korea |
| KR052754.1 | LS00006_OSU/1975 | 1975 | USA |
| MF940681.1 | KJ19-2/2006 | 2006 | Korea |
| KF500193.1 | KF500193.1 42-1/2006 | 2006 | Korea |
| OQ743754.1 | YN-A/2021 | 2021 | China |
| MF940682.1 | KJ19-2/2006 | 2006 | Korea |
| MF940623.1 | KJ11/2006 | 2006 | Korea |
| JQ309143.1 | GBR/H-1/1975 | 1975 | UK |
| KT694947.1 | Wa/1974 | 1974 | USA |
| PQ127088.2 | IRN/502312/2021 | 2021 | Iran |
| PP862051.1 | Fuzhou23-93/2023 | 2023 | China |
| PP862019.1 | Pingtan21-4/2021 | 2021 | China |
| MN106173.1 | Z2761/2019 | 2019 | China |
| KX655525.1 | MUL-13-427/2013 | 2013 | Uganda |
| AB930199.1 | S140023/2014 | 2014 | Japan |
| KP941134.1 | Keny-061/2008 | 2008 | Kenya |
| KP883206.1 | Mali-137/2008 | 2008 | Mali |
| KP882678.1 | Ghan-148/2007 | 2007 | Ghana |
| AB848012.1 | HC12016/2012 | 2012 | Japan |
| OR756445.1 | VE17426/2020 | 2020 | Viet Nam |
| DQ490535.1 | AU-1/1982 | 1982 | Japan |
| AB009626.2 | PO-3/1983 | 1983 | Japan |
| GQ479953.1 | ETD-882/2007 | 2007 | USA |

Table S10. Porcine rotavirus strains used in the evolutionary analysis of the NSP4 gene.

| Accession | Isolate | Collection Date | Geo Location |
| --- | --- | --- | --- |
| KJ126820.1 | LLP48/2008 | 2008 | China |
| KU886311.1 | HLJ | 2015 | China |
| MN102374.1 | GHA/14/2016 | 2016 | Ghana |
| ON676178.1 | SD-1/2021 | 2021 | China |
| MK597969.1 | SCLS-X1/2018 | 2018 | China |
| EF159572.1 | LL3354/2000 | 2000 | China |
| KJ466989.1 | YN/2012 | 2012 | China |
| KC139788.1 | LL3354/2000 | 2000 | China |
| PV631384.1 | SQ-23/2023 | 2023 | China |
| GQ240627.1 | mani-362/07/2008 | 2008 | India |
| EF159574.1 | LL36755/2003 | 2003 | China |
| ON093978.1 | FJSH01/2021 | 2021 | China |
| PQ724865.1 | ZT5159/2023 | 2023 | China |
| OR911933.1 | GD/2022 | 2022 | China |
| MT784855.1 | MZ-MPT-200/2016 | 2016 | Mozambique |
| LC095930.1 | NT0205/2007 | 2007 | Viet Nam |
| PQ581891.1 | CH/10.2-20/2022 | 2022 | China |
| MK283698.1 | P828/2015 | 2015 | Czech Republic |
| PQ724872.1 | ZT2130/2023 | 2023 | China |
| MK283699.1 | P830/2015 | 2015 | CZE |
| MG407654.1 | rj24598/2015 | 2015 | Brazil |
| KC580432.1 | DC1292/1980 | 1980 | USA |
| LC095941.1 | NT0599/2008 | 2008 | Viet Nam |
| KC579689.1 | DC104/1974 | 1974 | USA |
| LC095919.1 | NT0077/2007 | 2007 | Viet Nam |
| EF672603.1 | USA/P/1974 | 1974 | USA |
| LC095952.1 | NT0621/2008 | 2008 | Viet Nam |
| PQ133257.1 | NG523/2022 | 2022 | China |
| JQ069177.1 | RT178-07/2008 | 2008 | Canada |
| JN258345.1 | 2007719685/2007 | 2007 | Barbados |
| KY497549.1 | PAK/42/2010 | 2010 | Pakistan |
| KC580107.1 | DC581/1979 | 1979 | USA |
| EF672575.1 | USA/D/1974 | 1974 | USA |
| ON855318.1 | F01482/2009 | 2009 | Belgium |
| MT874992.1 | NJ2012/2012 | 2012 | China |
| KF500194.1 | 42-1/2006 | 2006 | Korea |
| MF940465.1 | K71/2006 | 2006 | Korea |
| JX971578.1 | K5/2004 | 2004 | Korea |
| KF500216.1 | 174-1/2006 | 2006 | Korea |
| PP235805.1 | GDZHF/2023 | 2023 | China |
| PQ586686.1 | YNDL/2023 | 2023 | China |
| OQ743755.1 | YN-A/2021 | 2021 | China |
| KF500227.1 | C-1/2006 | 2006 | Korea |
| PV500793.1 | YNKM/2023 | 2023 | China |
| MF940576.1 | 174-1/2006 | 2006 | Korea |
| MF940629.1 | KJ11/2006 | 2006 | Korea |
| MF940630.1 | KJ11/2006 | 2006 | Korea |
| OP978247.1 | OSU/1975 | 1975 | USA |
| AF541921.1 | RMC321/2002 | 2002 | India |
| LC776523.1 | C-Sh/2022 | 2022 | Japan |
| LC433783.1 | TK1797/2007 | 2007 | Nepal |
| KT694948.1 | Wa/1974 | 1974 | USA |
| PQ127091.1 | IRN/502312/2021 | 2021 | Iran |
| PP862133.1 | Fuzhou23-93/2023 | 2023 | China |
| PP862100.1 | Pingtan21-2/2021 | 2021 | China |
| ON012983.1 | SCMY2/2021 | 2021 | China |
| ON993101.1 | SZ18442205/2018 | 2018 | China |
| ON993091.1 | GD18442033/2018 | 2018 | China |
| ON993077.1 | SC18511025/2018 | 2018 | China |
| ON993050.1 | JL18221297/2018 | 2018 | China |
| ON993026.1 | SZ18442055/2018 | 2018 | China |
| MN529657.1 | JZ1812/2018 | 2018 | China |
| LC158125.1 | LUS12-14/2012 | 2012 | Zambia |
| KY055436.1 | BUW-14-085/2014 | 2014 | Uganda |
| KX655526.1 | MUL-13-427/2013 | 2013 | Uganda |
| AB930200.1 | S140023/2014 | 2014 | Japan |

Table S11. Porcine rotavirus strains used in the evolutionary analysis of the NSP5 gene.

| Accession | Isolate | Collection Date | Geo Location |
| --- | --- | --- | --- |
| PQ323314.1 | GZ/2023 | 2023 | China |
| MH910073.1 | SCCD-A/2017 | 2017 | China |
| MK227397.1 | H14020027 | 2014 | Bangladesh |
| DQ003299.1 | HP140/2005 | 2005 | India |
| MK250434.1 | HY-1/2018 | 2018 | China |
| LC433784.1 | TK1797/2007 | 2007 | Nepal |
| MH137270.1 | SCLSHL-2-3/2017 | 2017 | China |
| LC569901.1 | PK2015-1-0001 | 2015 | Thailand |
| KF041439.1 | GX54/2010 | 2010 | China |
| PV631385.1 | SQ-23/2023 | 2023 | China |
| AY033396.1 | RMC321/2001 | 2001 | India |
| KX363314.1 | VNM/12129_48 | 2012 | Viet Nam |
| KF726043.1 | E931/2008 | 2008 | China |
| PQ141605.1 | 923E/2021 | 2021 | China |
| MH898997.1 | SCJY-5/2017 | 2017 | China |
| MF462320.1 | LNCY/2016 | 2016 | China |
| LC765817.1 | RVN17.0271/2017 | 2017 | Viet Nam |
| KF835970.1 | BP1125/2004 | 2004 | Hungary |
| MN224001.1 | MNov05-394/2005 | 2005 | Russia |
| LC389884.1 | R1207/2009 | 2009 | Sri Lanka |
| PQ133258.1 | NG523/2022 | 2022 | China |
| MH910084.1 | SCJY-11/2017 | 2017 | China |
| PQ299951.1 | C48-VS/2020 | 2020 | Croatia |
| MN224029.1 | Nov12-N5289/2012 | 2012 | Russia |
| KF835971.1 | BP1227/2002 | 2002 | Hungary |
| OP886872.1 | CN1P7/2021 | 2021 | China |
| KF835974.1 | BP1547/2005 | 2005 | Hungary |
| FJ206056.1 | KJ20/2008 | 2008 | Korea |
| PP235806.1 | GDZHF/2023 | 2023 | China |
| MF940633.1 | KJ11/2006 | 2006 | Korea |
| FJ206094.1 | KJ212/2008 | 2008 | Korea |
| KF500195.1 | KOR/42-1 | 2006 | Korea |
| MT874993.1 | NJ2012/2012 | 2012 | China |
| MF940468.1 | K71/2006 | 2006 | Korea |
| OP978248.1 | OSU/1975 | 1975 | USA |
| GU329526.1 | CH-1/2009 | 2009 | China |
| MF940472.1 | K71/2006 | 2006 | Korea |
| PP100170.1 | JSNJ2019/2019 | 2019 | China |
| LC190496.1 | KKL-117/2014 | 2014 | Thailand |
| KU363143.1 | CMHS-070-13/2013 | 2013 | Thailand |
| MN066881.1 | CMC_00052/2010 | 2010 | India |
| MG781041.1 | CMH-N016-10/2010 | 2010 | Thailand |
| GU199491.1 | Gottfried/1975 | 1975 | USA |
| MG781051.1 | CMH-N014-11/2011 | 2011 | Thailand |
| MN066810.1 | CMC_00038/2011 | 2011 | India |
| OR772035.1 | NN485-23/2023 | 2023 | Russia |
| KT694949.1 | Wa/1974 | 1974 | USA |
| PQ127094.1 | IRN/502312/2021 | 2021 | India |
| PP862215.1 | Fuzhou23-93/2023 | 2023 | China |
| PP862183.1 | Pingtan21-4/2021 | 2021 | China |
| KX655505.1 | MUL-13-160/2013 | 2013 | Uganda |
| KX655516.1 | MSK-13-048/2013 | 2013 | Uganda |
| AB930201.1 | S140023/2014 | 2014 | Japan |
| KP883186.1 | Mali-135/2008 | 2008 | Mali |
| KP882603.1 | Ghan-107/2009 | 2009 | Ghana |
| KP882097.1 | Bang-090/2008 | 2008 | Bangladesh |
| KT006917.1 | SanSebastian98244047_DS-1/2015 | 2015 | Spain |
| MG701225.1 | DOM/3000503731/2016 | 2016 | DR |
| ON792046.1 | BTY25L/2018 | 2018 | Malawi |
| MT767407.1 | Moscow-714/2014 | 2014 | Russia |
| KU550327.1 | SS98244047/2015 | 2015 | Spain |
