# Supplementary material for: Whole-genome characterization and pathogenicity of novel human-porcine reassortant rotavirus strains G9P[7] and G1P[7] in China
Source: Vet Res. 2026 Jul 15;57:135. doi: 10.1186/s13567-026-01775-1 (PMC13371254; doi:10.1186/s13567-026-01775-1)
Supplement: Supplementary file 2 — Additional file 2. Porcine rotavirus strains used in the evolutionary analysis of the VP7 gene. [file 13567_2026_1775_MOESM2_ESM.docx]

**Additional file 2 Porcine rotavirus strains used in the evolutionary analysis of the VP7 gene.**

| Accession | Isolate | Collection Date | Geo Location |
| --- | --- | --- | --- |
| OR948019.1 | DB/DPD/2022 | 2022 | China |
| OP886878.1 | CN1P7/2021 | 2021 | China |
| PV390286.1 | HeN/08484/2022 | 2022 | China |
| PV390278.1 | HB/06398/2022 | 2022 | China |
| OQ743893.1 | SD/LCXH03/2022 | 2022 | China |
| EF690750.1 | Agroj23/2002 | 2002 | Bangladesh |
| JN129113.1 | NCA/9J/2010 | 2010 | USA |
| HQ392122.1 | BEL/BE00017/2006 | 2006 | USA |
| JX027818.1 | AUS/CK00083/2008 | 2008 | USA |
| AF480292.1 | Mvd9815/2003 | 2003 | Uruguay |
| DQ512996.1 | Chi-83/2007 | 2007 | Japan |
| AF480296.1 | Mvd9614/2003 | 2003 | Uruguay |
| JX943614.2 | Rotarix/2009 | 2009 | USA |
| JQ926436.1 | se15901-08/2008 | 2008 | Brazil |
| LC028930.1 | OSN9-Rx/2014 | 2014 | Japan |
| KT694944.1 | Wa/1974 | 1974 | USA |
| GU565057.1 | RotaTeq-WI79-9/1992 | 1992 | USA |
| AB081796.1 | 89H452/2002 | 2002 | Japan |
| DQ377587.1 | PA10/90/2006 | 2006 | Italy |
| AB018697.1 | AU19/1999 | 1999 | Japan |
| U26366.1 | Ban-59/1996 | 1996 | USA |
| U26373.1 | Egypt-7/1996 | 1996 | USA |
| DQ377567.1 | PA17c/86/2006 | 2006 | Italy |
| AF426162.1 | SW20/21/2001 | 2001 | UK |
| L24164.1 | ARG/C95 | 1994 | ARG |
| M92651.1 | ARG/T449 | 1992 | ARG |
| L24165.1 | VEN/C95 | 1994 | ARG |
| GU188284.1 | CN1P7/2021 | 2021 | China |
| GU124595.1 | sh0902/2009 | 2009 | China |
| MN862194.1 | Nebraska33/2010 | 2010 | USA |
| PQ314329.1 | YNXD/2024 | 2024 | China |
| OR475446.1 | GD2101/2023 | 2023 | China |
| PQ586690.1 | YNDL/2023 | 2023 | China |
| MT874991.1 | NJ2012/2012 | 2012 | China |
| AB180969.1 | WI61/1983 | 1983 | USA |
| AB180970.1 | F45/1983 | 1983 | Japan |
| L14072.1 | 116E/1990 | 1990 | India |
| EF990708.1 | B3458/2003 | 2003 | Belgium |
| JQ253563.1 | CAU08-463/2008 | 2008 | Korea |
| KF673477.1 | BJ-Q33/2010 | 2010 | China |
| KP752521.1 | TGO/MRC-DPRU5123/2010 | 2010 | Korea |
| D38055.1 | Mc345/2000 | 2000 | Japan |
| AF281044.1 | IECIT-254/2002 | 2002 | Japan |
| DQ207390.1 | 17025-03/2002 | 2002 | Ireland |
| AF260959.1 | 97'SZ37/2000 | 2000 | China |
| AJ491179.1 | OM67/2003 | 2003 | USA |
| AJ491181.1 | USA/ OM46/2003 | 2003 | USA |
| KT007646.1 | CU-B1670/KK/2012 | 2012 | Thailand |
| LC105445.1 | UR14-17/2014 | 2014 | Japan |
| KF673484.1 | BJ-Q1141/2013 | 2013 | China |
| KC200154.1 | SPH0144/2013 | 2013 | China |
| KF673488.1 | BJ-CR7818/2012 | 2012 | China |
| KT919508.1 | VU12-13-101/2013 | 2013 | USA |
| AB045374.1 | K-1/2000 | 2000 | Japan |
