# Supplementary material for: Whole-genome characterization and pathogenicity of novel human-porcine reassortant rotavirus strains G9P[7] and G1P[7] in China
Source: Vet Res. 2026 Jul 15;57:135. doi: 10.1186/s13567-026-01775-1 (PMC13371254; doi:10.1186/s13567-026-01775-1)
Supplement: Supplementary file 3 — Additional file 3. Porcine rotavirus strains used in the evolutionary analysis of the VP6 gene. [file 13567_2026_1775_MOESM3_ESM.docx]

**Additional file 3 Porcine rotavirus strains used in the evolutionary analysis of the VP6 gene.**

| Accession | Isolate | Collection Date | Geo Location |
| --- | --- | --- | --- |
| OR094874.1 | HB-1RV/2023 | 2023 | China |
| OR947947.1 | AH/SS/2210244/2022 | 2022 | China |
| OQ799845.1 | HeN/TQ2/2022 | 2022 | China |
| PP053570.1 | DB/LC/2310133/2023 | 2023 | China |
| OQ799846.1 | LN/YTW1/2022 | 2022 | China |
| PV390411.1 | GD/06308/2022 | 2022 | China |
| PV390437.1 | JS/10277/2022 | 2022 | China |
| OQ799848.1 | LN/YTW2/2022 | 2022 | China |
| MK026439.1 | SCMY-A3/2017 | 2017 | China |
| OQ799856.1 | SD/CY3/2022 | 2022 | China |
| PV430833.1 | TJ/07181-4/2023 | 2023 | China |
| MH910067.1 | SCCD-A/2017 | 2017 | China |
| OQ799820.1 | CY/LH5/2022 | 2022 | China |
| OQ799814.1 | SD/CYFMD2/2022 | 2022 | China |
| OQ799829.1 | CY/LH8/2022 | 2022 | China |
| PV430834.1 | TJ/07181-5/2023 | 2023 | China |
| OQ799833.1 | ZJ/CH3/2022 | 2022 | China |
| PQ452948.1 | HUBEI/2022 | 2022 | China |
| JN104614.1 | Mc323/2011 | 2011 | Thailand |
| LC095879.1 | VNM/NT0001/2007 | 2007 | Japan |
| OR094869.1 | GD-1RV/2023 | 2023 | China |
| MT874988.1 | NJ2012/2012 | 2012 | China |
| FJ617209.1 | GD/2009 | 2009 | China |
| PP235801.1 | GDZHF/2023 | 2023 | China |
| PQ586691.1 | YNDL/2023 | 2023 | China |
| PQ452937.1 | HUBEI/2022/5.11/u | 2022 | China |
| JF796738.1 | PRG9121/2012 | 2012 | Korea |
| KR052760.1 | LS00006_OSU/1975 | 1975 | USA |
| AY538664.1 | JL94/2004 | 2004 | China |
| JX971573.1 | K5/2012 | 2012 | Korea |
| MF940439.1 | KOR/K71/2006 | 2006 | Korea |
| MF940717.1 | KJ44/2006 | 2006 | Korea |
| MF940552.1 | KOR/174-1/2006 | 2006 | Korea |
| GU188283.1 | CH-1/2009 | 2009 | China |
| LC774619.1 | SO1199/2020 | 2020 | Japan |
| MF940603.1 | KJ11/2006 | 2006 | Korea |
| MH308723.1 | OK.5.68/2008 | 2008 | USA |
| MH267277.1 | MN9.65b/2008 | 2008 | USA |
| PV430898.1 | HeN/10097-39/2023 | 2023 | China |
| PV430912.1 | SC/10382/2023 | 2023 | China |
| HM534677.1 | USA/2009727093/2009 | 2009 | USA |
| KX655532.1 | BUW-14-A035/2014 | 2014 | Uganda |
| KX655488.1 | UGA/KTV-13-023/2013 | 2013 | Uganda |
| PP861560.1 | Fuzhou23-140/2023 | 2023 | China |
| PP682347.1 | AHBZ2312/2023 | 2023 | China |
| ON563404.1 | BJ-Q1087/2012 | 2012 | China |
| ON992639.1 | SZ18442196/2018 | 2018 | China |
| ON992632.1 | GD18442035/2018 | 2018 | China |
| ON992619.1 | SC18511086/2018 | 2018 | China |
| ON992594.1 | JL18221381/2018 | 2018 | China |
| ON992565.1 | SZ18442011/2018 | 2018 | China |
| OM037874.1 | HEB16231045/2016 | 2016 | China |
| MN106125.1 | E5365/2017 | 2017 | China |
| MG066585.1 | SCLS-2-3/2017 | 2017 | China |
| KU887648.1 | P70/2015 | 2015 | Czech Republic |
| PP585955.1 | HGJM0413/2015 | 2015 | Mozambique |
| MT796883.1 | Wa/1974 | 1974 | USA |
| KX655521.1 | MUL-13-427/2013 | 2013 | Uganda |
| PP861559.1 | Fuzhou23-93/2023 | 2023 | China |
| MT339196.1 | Ph158/2020 | 2020 | USA |
| AB930193.1 | AS140023/2014/ | 2014/ | Japan |
| KP882606.1 | Ghan-108/2009 | 2009 | Ghana |
| KP882573.1 | Ghan-105/2009 | 2009 | Ghana |
| KP882276.1 | Bang-143/2008 | 2008 | Bangladesh |
| KJ721704.1 | RJ12225/2006 | 2006 | Brazil |
| MG670599.1 | DOM/3000503705/2014 | 2014 | USA |
| MN067459.1 | S18/2012 | 2012 | Morocco |
| MN067448.1 | S19/2012 | 2012 | Morocco |
| PP848777.1 | HCN1604/2017 | 2017 | Mozambique |
| AB796454.1 | OH3625/2012 | 2012 | Japan |
