# Supplementary material for: Whole-genome characterization and pathogenicity of novel human-porcine reassortant rotavirus strains G9P[7] and G1P[7] in China
Source: Vet Res. 2026 Jul 15;57:135. doi: 10.1186/s13567-026-01775-1 (PMC13371254; doi:10.1186/s13567-026-01775-1)
Supplement: Supplementary file 4 — Additional file 4. Porcine rotavirus strains used in the evolutionary analysis of the VP1 gene. [file 13567_2026_1775_MOESM4_ESM.docx]

**Additional file 4 Porcine rotavirus strains used in the evolutionary analysis of the VP1 gene.**

| Accession | Isolate | Collection Date | Geo Location |
| --- | --- | --- | --- |
| KF726036.1 | E931/2008 | 2008 | China |
| KF041441.1 | GX54/2013 | 2013 | China |
| MK410286.1 | SWU-1C/2018 | 2018 | China |
| LC095880.1 | NT0001/2007 | 2007 | Viet Nam |
| KF726069.1 | R1954/2013 | 2013 | China |
| MH624173.1 | SC11/2017 | 2017 | China |
| MT876637.1 | Moscow-1P/2015 | 2015 | Russia |
| PQ141626.1 | 923X/2021 | 2021 | China |
| PQ141606.1 | 923E/2021 | 2021 | China |
| MH137269.1 | SCLSHL-2-3/2017 | 2017 | China |
| OP082201.1 | Morogoro-RP074/2019 | 2019 | Tanzania |
| GU189551.1 | R479/2009 | 2009 | China |
| OP082196.1 | Iringa-IP058/2019 | 2019 | Tanzania |
| LC095902.1 | NT0073/2007 | 2007 | Viet Nam |
| LC095935.1 | NT0599/2008 | 2008 | Viet Nam |
| PQ586684.1 | YNXD/2023 | 2023 | China |
| PP235798.1 | GDZHF/2023 | 2023 | China |
| OQ743746.1 | YN-A/2021 | 2021 | China |
| MT874983.1 | NJ2012/2012 | 2012 | China |
| PQ452922.1 | SHANXI/2022/3.14/E | 2022 | China |
| PV500801.1 | YNKM/2023 | 2023 | China |
| KC140590.1 | CAU12-2/2012 | 2012 | Korea |
| HM773898.1 | DC4608/1980 | 1980 | USA |
| JN129055.1 | OL/2010 | 2010 | Nicaragua |
| KC579564.1 | DC1476/1974 | 1974 | USA |
| OR683327.1 | HBP478/2021 | 2021 | China |
| LC776551.1 | N-Fu1/2022 | 2022 | Japan |
| OR683316.1 | HBP445/2021 | 2021 | China |
| PQ300009.1 | DS404-VS/2020 | 2020 | Croatia |
| KX655438.1 | MUL-13-171/2013 | 2013 | Uganda |
| PQ127097.1 | IRN/502312/2021 | 2021 | Iran |
| PP861641.1 | Fuzhou23-93/2023 | 2023 | China |
| PP861608.1 | Pingtan21-2/2021 | 2021 | China |
| KX655517.1 | MUL-13-427/2013 | 2013 | Uganda |
| AB930194.1 | S140023/2014 | 2014 | Japan |
| KJ721726.1 | MS11142/2005 | 2005 | Brazil |
| DQ205221.1 | ITA/30-96/1996 | 1996 | Italy |
| MH291387.1 | KEN/3994/2017 | 2017 | Kenya |
| MN067444.1 | S19/2012 | 2012 | Morocco |
| MH291383.1 | KEN/3946/2017 | 2017 | Kenya |
| MF940638.1 | KJ19-2/2006 | 2006 | Korea |
| KU356640.1 | M292/2013 | 2013 | Bangladesh |
| KJ639023.1 | S13-45/2013 | 2013 | Japan |
| KC257091.1 | MRC-DPRU447/2002 | 2002 | Sudan |
