# Supplementary material for: Whole-genome characterization and pathogenicity of novel human-porcine reassortant rotavirus strains G9P[7] and G1P[7] in China
Source: Vet Res. 2026 Jul 15;57:135. doi: 10.1186/s13567-026-01775-1 (PMC13371254; doi:10.1186/s13567-026-01775-1)
Supplement: Supplementary file 5 — Additional file 5. Porcine rotavirus strains used in the evolutionary analysis of the VP2 gene. [file 13567_2026_1775_MOESM5_ESM.docx]

**Additional file 5 Porcine rotavirus strains used in the evolutionary analysis of the VP2 gene.**

| Accession | Isolate | Collection Date | Geo Location |
| --- | --- | --- | --- |
| PP975111.1 | rJXAY01 | 2024 | China |
| KJ466983.1 | YN/2012 | 2012 | China |
| MK597961.1 | SCLS-X1/2018 | 2018 | China |
| PQ323316.1 | GZ/2023 | 2023 | China |
| OQ979281.1 | AHFY2022/2022 | 2022 | China |
| PQ452923.1 | SHANXI/2022/3.14/E | 2022 | China |
| KF726037.1 | E931/2008 | 2008 | China |
| KF726059.1 | R946/2006 | 2006 | China |
| OP886874.1 | CN1P7/2021 | 2021 | China |
| OR683306.1 | HBP442/2021 | 2021 | China |
| LC765811.1 | RVN17.0271/2017 | 2017 | Viet Nam |
| MH137268.1 | SCLSHL-2-3/2017 | 2017 | China |
| PV164607.1 | HB2022/2019 | 2019 | China |
| JX290176.1 | TM-a/2009 | 2009 | China |
| MH697614.1 | TM-a-P1/2018 | 2018 | China |
| KU886314.1 | HLJ/15/1/2015 | 2015 | China |
| MH697647.1 | TM-a-P60/2018 | 2018 | China |
| KC579565.1 | DC1476/1974 | 1974 | USA |
| JF781159.1 | NMTL/2008 | 2008 | China |
| JX416205.1 | M37/1982 | 1982 | Venezuela |
| KR052758.1 | LS00006_OSU/1975 | 1975 | USA |
| PQ452934.1 | HUBEI/2022/5.11/u | 2022 | China |
| KF500175.1 | KJ56-1/2004 | 2004 | Korea |
| GU199515.1 | OSU/1975 | 1975 | USA |
| JX971581.1 | K71/2006 | 2006 | Korea |
| KC580121.1 | DC1210/1980 | 1980 | USA |
| MT874984.1 | NJ2012/2012 | 2012 | China |
| KC580336.1 | DC576/1979 | 1979 | USA |
| MF940590.1 | KJ11/2006 | 2006 | Korea |
| MF940425.1 | K71/2006 | 2006 | Korea |
| KC579736.1 | DC1212/1980 | 1980 | USA |
| LC438922.1 | RRV_NB1215_31/1987 | 1987 | Venezuela |
| PP861723.1 | Fuzhou23-93/2023 | 2023 | China |
| PP861691.1 | Pingtan21-4/2021 | 2021 | China |
| ON012975.1 | SCMY2/2021 | 2021 | China |
| MT339198.1 | Ph158/1998 | 1998 | USA |
| LC158120.1 | LUS12-14/2012 | 2012 | Zambia |
| KY055428.1 | BUW-14-085/2014 | 2014 | Uganda |
| KX655507.1 | MSK-13-048/2013 | 2013 | Uganda |
| KX655474.1 | MUL-12-117/2012 | 2012 | Uganda |
| KJ940064.1 | SC19868/2011 | 2011 | Brazil |
| OR194469.1 | CHN/22160307/2022 | 2022 | China |
| MH291368.1 | KEN/3920/2017 | 2017 | Kenya |
| KY497552.1 | PAK93/2010 | 2010 | Pakistan |
| MF940643.1 | KJ19-2/2006 | 2006 | Korea |
