# Supplementary material for: Whole-genome characterization and pathogenicity of novel human-porcine reassortant rotavirus strains G9P[7] and G1P[7] in China
Source: Vet Res. 2026 Jul 15;57:135. doi: 10.1186/s13567-026-01775-1 (PMC13371254; doi:10.1186/s13567-026-01775-1)
Supplement: Supplementary file 6 — Additional file 6. Porcine rotavirus strains used in the evolutionary analysis of the VP3 gene. [file 13567_2026_1775_MOESM6_ESM.docx]

**Additional file 6 Porcine rotavirus strains used in the evolutionary analysis of the VP3 gene.**

| Accession | Isolate | Collection Date | Geo Location |
| --- | --- | --- | --- |
| KF726038.1 | E931/2008 | 2008 | China |
| KF726060.1 | R946/2006 | 2006 | China |
| OP886875.1 | CN1P7/2021 | 2021 | China |
| MT271030.1 | UFS-NGS-MRC-DPRU4723/2014 | 2014 | Zambia |
| OR911926.1 | GD/2022 | 2022 | China |
| JN129083.1 | OL/2010 | 2010 | Nicaragua |
| MK597973.1 | SCLS-3/2018 | 2018 | China |
| MK597984.1 | SCLS-R3/2018 | 2018 | China |
| PP391052.1 | BH/2023 | 2023 | China |
| MH238264.1 | F471/2017 | 2017 | Spain |
| OR683351.1 | HBP453/2021 | 2021 | China |
| HQ641295.1 | CH-1/2008 | 2008 | China |
| ON676182.1 | SD-1/2021 | 2021 | China |
| PQ299924.1 | L352-K/2019 | 2019 | Croatia |
| OP082228.1 | Iringa-IP057/2019 | 2019 | Tanzania |
| PQ299957.1 | DS76-K/2018 | 2018 | Croatia |
| KM820710.1 | 12R005/2012 | 2012 | Belgium |
| KC139783.1 | LL3354/2000 | 2000 | China |
| MG407647.1 | rj24598/2015 | 2015 | Brazil |
| LC777994.1 | M-HDK9/2021 | 2021 | Japan |
| KF726071.1 | R1954/2013 | 2013 | China |
| MT784806.1 | MZ-MPT-115/2016 | 2016 | Mozambique |
| PQ299902.1 | S280-SD/2019 | 2019 | Croatia |
| KF835911.1 | BP1792/2004 | 2004 | Hungary |
| LC095926.1 | NT0205/2007 | 2007 | Viet Nam |
| PQ299968.1 | DS84-Z/2018 | 2018 | Croatia |
| LC765812.1 | RVN17.0271/2017 | 2017 | Viet Nam |
| KT694941.1 | Wa/1974 | 1974 | USA |
| PP861805.1 | Fuzhou23-93/2023 | 2023 | China |
| ON012976.1 | SCMY2/2021 | 2021 | China |
| MT339199.1 | Ph158/1998 | 1998 | USA |
| MT276808.1 | CC425/1998 | 1998 | USA |
| KY055429.1 | BUW-14-085/2014 | 2014 | Uganda |
| KX655519.1 | MUL-13-427/2013 | 2013 | Uganda |
| KX655453.1 | MUL-13-204/2013 | 2013 | Uganda |
| KP883181.1 | Mali-135/2008 | 2008 | Mali |
| KP882675.1 | Ghan-148/2007 | 2007 | Ghana |
| KP882279.1 | Bang-143/2008 | 2008 | Bangladesh |
| AB849002.1 | S120088/2012 | 2012 | Japan |
| KJ721717.1 | MS11142/2005 | 2005 | Brazil |
| OR194482.1 | CHN/22160302/2022 | 2022 | China |
| MH291352.1 | KEN/4009/2017 | 2017 | Kenya |
| MN067446.1 | S19/2012 | 2012 | Morocco |
| KU550277.1 | SS61921417/2015 | 2015 | Spain |
