# Supplementary material for: Whole-genome characterization and pathogenicity of novel human-porcine reassortant rotavirus strains G9P[7] and G1P[7] in China
Source: Vet Res. 2026 Jul 15;57:135. doi: 10.1186/s13567-026-01775-1 (PMC13371254; doi:10.1186/s13567-026-01775-1)
Supplement: Supplementary file 7 — Additional file 7. Porcine rotavirus strains used in the evolutionary analysis of the NSP1 gene. [file 13567_2026_1775_MOESM7_ESM.docx]

**Additional file 7 Porcine rotavirus strains used in the evolutionary analysis of the NSP1 gene.**

| Accession | Isolate | Collection Date | Geo Location |
| --- | --- | --- | --- |
| PQ141601.1 | 923E/2021 | 2021 | China |
| PP566181.1 | GDFZ/2023 | 2023 | China |
| PQ300012.1 | DS404-VS/2020 | 2020 | Croatia |
| OM982781.1 | S20-0073/2020 | 2020 | Switzerland |
| KM820740.1 | 12R022/2012 | 2012 | Belgium |
| KM820737.1 | 12R002/2012 | 2012 | Belgium |
| KJ412559.1 | 1809SR/2009 | 2009 | Paraguay |
| PQ299980.1 | DS229-Z/2020 | 2020 | Croatia |
| PQ299947.1 | C48-VS/2020 | 2020 | Croatia |
| KF835937.1 | BP271/2000 | 2000 | Hungary |
| OR192581.1 | Rota_1a/2017 | 2017 | Chile |
| KJ659437.1 | LS00008/1975 | 1975 | USA |
| JQ993324.1 | BE2001/2009 | 2009 | Belgium |
| AB741655.1 | Ryukyu-1120/2011 | 2011 | Japan |
| PP874432.1 | MR-22/2022 | 2022 | Russia |
| KX988270.1 | KYE-14-A047/2014 | 2014 | Uganda |
| KM820738.1 | 12R005/2012 | 2012 | Belgium |
| MH238095.1 | F456/2017 | 2017 | Spain |
| PP003809.1 | GL/2022 | 2022 | China |
| MN102369.1 | 14/2016 | 2016 | Ghana |
| PQ141611.1 | 923H/2021 | 2021 | China |
| OR911930.1 | GD/2022 | 2022 | China |
| PQ452950.1 | HUBEI/2022 | 2022 | China |
| KX363405.1 | VNM/14226_39 | 2012 | Viet Nam |
| HG513049.1 | VNM/30378/2009 | 2009 | Viet Nam |
| LC569886.1 | DU2014-259/2014 | 2014 | Thailand |
| LC569897.1 | PK2015-1-0001 | 2015 | Thailand |
| LC776543.1 | A-Ta1/2022 | 2022 | Japan |
| OR756389.1 | VE5852/2020 | 2020 | Viet Nam |
| KY937198.1 | CC9192/2014 | 2014 | Cambodia |
| JN104623.1 | Mc345/2011 | 2011 | Japan |
| KF726039.1 | E931/2008 | 2008 | China |
| PV026147.1 | RHeN2/2021 | 2021 | China |
| JQ309141.1 | H-1/1975 | 1975 | UK |
| KF726072.1 | R1954/2013 | 2013 | China |
| MH137274.1 | SCLSHL-2-3/2017 | 2017 | China |
| LC776554.1 | N-Fu1/2022 | 2022 | Japan |
| HM773847.1 | 2007719907/2007 | 2007 | USA |
| KX655534.1 | BUW-14-A035/2014 | 2014 | Uganda |
| KX655490.1 | KTV-13-023/2013 | 2013 | Uganda |
| PP861888.1 | Fuzhou23-140/2023 | 2023 | China |
| ON563413.1 | BJ-Q1087/2012 | 2012 | China |
| ON992526.1 | SZ18442205/2018 | 2018 | China |
| ON992518.1 | GD18442038/2018 | 2018 | China |
| ON992487.1 | SD18370064/2018 | 2018 | China |
| OM037851.1 | GX16451181/2016 | 2016 | China |
| MN106155.1 | E6356/2019 | 2019 | China |
| KT694945.1 | Wa/1974 | 1974 | USA |
| KP883050.1 | Mali-050/2008 | 2008 | MLI |
| OQ440165.1 | D230-ZG/2019 | 2019 | Croatia |
| KX632249.1 | NSA-13-043/2013 | 2013 | Uganda |
| MF940724.1 | KJ44/2006 | 2006 | South Korea |
| DQ146655.1 | Dhaka25/2002 | 2002 | Bangladesh |
| AB930197.1 | S140023/2014 | 2014 | Japan |
| KX655468.1 | MUL-13-496/2013 | 2013 | Uganda |
| PP861855.1 | Pingtan21-4/2021 | 2021 | China |
| PP861887.1 | Fuzhou23-93/2023 | 2023 | China |
| KP941132.1 | Keny-061/2008 | 2008 | Kenya |
| KP882687.1 | Ghan-149/2008 | 2008 | Ghana |
| KP882192.1 | Bang-114/2008 | 2008 | Bangladesh |
| OR194488.1 | 22160303/2022 | 2022 | China |
| LC095894.1 | VNM/NT0042 | 2007 | Viet Nam |
