# Supplementary material for: Whole-genome characterization and pathogenicity of novel human-porcine reassortant rotavirus strains G9P[7] and G1P[7] in China
Source: Vet Res. 2026 Jul 15;57:135. doi: 10.1186/s13567-026-01775-1 (PMC13371254; doi:10.1186/s13567-026-01775-1)
Supplement: Supplementary file 8 — Additional file 8. Porcine rotavirus strains used in the evolutionary analysis of the NSP2 gene. [file 13567_2026_1775_MOESM8_ESM.docx]

**Additional file 8 Porcine rotavirus strains used in the evolutionary analysis of the NSP2 gene.**

| Accession | Isolate | Collection Date | Geo Location |
| --- | --- | --- | --- |
| PQ299992.1 | DS306-OB/2020 | 2020 | Croatia |
| OR192609.1 | Rota_2b/2017 | 2017 | Chile |
| KC155681.1 | Nov10-N459/2010 | 2010 | Russia |
| OR911931.1 | GD/2022 | 2022 | China |
| OP886869.1 | CN1P7/2021 | 2021 | China |
| PP025952.1 | 05E/2023 | 2023 | China |
| MH910070.1 | SCCD-A/2017 | 2017 | China |
| OM362100.1 | FX17/2021 | 2021 | China |
| PQ724870.1 | ZT2130/2023 | 2023 | China |
| PQ141622.1 | 923X/2021 | 2021 | China |
| PQ323311.1 | GZ/2023 | 2023 | China |
| KJ466987.1 | YN/2012 | 2012 | China |
| PQ581889.1 | 10.2-20/2022 | 2022 | China |
| PP566192.1 | S3CF/2023 | 2023 | China |
| PV631382.1 | SQ-23/2023 | 2023 | China |
| OM982753.1 | S18-1463/2018 | 2018 | Switzerland |
| KC020033.1 | Nov10-N806/2010 | 2010 | Russia |
| PQ299915.1 | L54-SM/2018 | 2018 | Croatia |
| PQ299981.1 | DS229-Z/2020 | 2020 | Croatia |
| PP669372.1 | UFS-BOC001/2018 | 2018 | South Africa |
| LC776555.1 | N-Fu1/2022 | 2022 | Japan |
| AB741656.1 | Ryukyu-1120/2011 | 2011 | Japan |
| OQ743753.1 | YN-A/2021 | 2021 | China |
| PQ586688.1 | YNDL/2023 | 2023 | China |
| PQ452940.1 | HUBEI/2022/5.11/u | 2022 | China |
| PP235803.1 | GDZHF/2023 | 2023 | China |
| PQ586677.1 | YNXD/2023 | 2023 | China |
| MT874990.1 | NJ2012/2012 | 2012 | China |
| JQ309142.1 | H-1/1975 | 1975 | UK |
| MT292016.1 | COD00155/1991 | 1991 | Brazil |
| KJ820876.1 | R70/1997 | 1997 | Brazil |
| KJ482279.1 | ROTA06/2013 | 2013 | Brazil |
| LC776485.1 | I-TP2/2021 | 2021 | Japan |
| KC020027.1 | O202/2007 | 2007 | Russia |
| MG926743.1 | 0440 | 2013 | MOZ |
| KF716402.1 | VU10-11-22 | 2011 | USA |
| ON792120.1 | BTY29D | 2018 | Malawi |
| MG573361.1 | IAL-R3165 | 2013 | Brazil |
| KU550306.1 | SS75915299 | 2015 | Spain |
| PP861969.1 | Fuzhou23-93 | 2023 | China |
| PP861965.1 | Fuzhou23-55 | 2023 | China |
| JF712584.1 | E403 | 2006 | ARG |
| JX567765.1 | V585 | 2011 | AUS |
| MW771169.1 | MPT-307 | 2016 | MOZ |
| HQ661141.1 | RV198-95 | 1995 | Italy |
| FJ347129.1 | Rio_Negro | 1998 | ARG |
| MF940674.1 | KJ19-2 | 2006 | Korea |
