# Supplementary material for: Whole-genome characterization and pathogenicity of novel human-porcine reassortant rotavirus strains G9P[7] and G1P[7] in China
Source: Vet Res. 2026 Jul 15;57:135. doi: 10.1186/s13567-026-01775-1 (PMC13371254; doi:10.1186/s13567-026-01775-1)
Supplement: Supplementary file 9 — Additional file 9. Porcine rotavirus strains used in the evolutionary analysis of the NSP3 gene. [file 13567_2026_1775_MOESM9_ESM.docx]

**Additional file 9 Porcine rotavirus strains used in the evolutionary analysis of the NSP3 gene.**

| Accession | Isolate | Collection Date | Geo Location |
| --- | --- | --- | --- |
| KJ466988.1 | YN/2012 | 2012 | China |
| MK597968.1 | SCLS-X1/2018 | 2018 | China |
| KX363440.1 | VNM/14250_9/2012 | 2012 | Viet Nam |
| LC095907.1 | NT0073/2007 | 2007 | Viet Nam |
| OM362101.1 | FX17/2021 | 2021 | China |
| LC095918.1 | NT0077/2007 | 2007 | Viet Nam |
| KC149936.1 | LL36755/2003 | 2003 | China |
| OR911932.1 | GD/2022 | 2022 | China |
| PP025951.1 | CHHeN/05E/2023 | 2023 | China |
| JX290174.1 | TM-a/2009 | 2009 | China |
| PQ452941.1 | HUBEI/2022/5.11/u | 2022 | China |
| LC776498.1 | K-Br29/2021 | 2021 | Japan |
| MH910071.1 | SCCD-A/2017 | 2017 | China |
| KJ482317.1 | ROTA21/2013 | 2013 | Brazil |
| OM982709.1 | SS3/2020 | 2020 | Switzerland |
| PQ299982.1 | DS229-Z/2020 | 2020 | Croatia |
| PQ299916.1 | L54-SM/2018 | 2018 | Croatia |
| OQ440167.1 | D230-ZG/2019 | 2019 | Croatia |
| AB779644.1 | CMP45/08/2008 | 2008 | Thailand |
| PQ300014.1 | DS404-VS/2020 | 2020 | Croatia |
| JN129013.1 | NCA/OL/2010 | 2010 | Nicaragua |
| MH238143.1 | F456/2017 | 2017 | Spain |
| JX971586.1 | K71/2006 | 2006 | Korea |
| MF940568.1 | KOR/174-1/2006 | 2006 | Korea |
| PP112344.1 | OSU-NSP3-2A-3xFL-Una | 2023 | USA |
| JX971577.1 | K5/2004 | 2004 | Korea |
| PQ586676.1 | YNXD/2023 | 2023 | China |
| MT874989.1 | NJ2012/2012 | 2012 | China |
| KF500182.1 | KJ56-1/2004 | 2004 | Korea |
| GU329525.1 | CH-1/2009 | 2009 | China |
| MF940458.1 | K71/2006 | 2006 | Korea |
| KR052754.1 | LS00006_OSU/1975 | 1975 | USA |
| OQ743754.1 | YN-A/2021 | 2021 | China |
| MF940682.1 | KJ19-2/2006 | 2006 | Korea |
| JQ309143.1 | GBR/H-1/1975 | 1975 | UK |
| KT694947.1 | Wa/1974 | 1974 | USA |
| PQ127088.2 | IRN/502312/2021 | 2021 | Iran |
| PP862051.1 | Fuzhou23-93/2023 | 2023 | China |
| PP862019.1 | Pingtan21-4/2021 | 2021 | China |
| MN106173.1 | Z2761/2019 | 2019 | China |
| KX655525.1 | MUL-13-427/2013 | 2013 | Uganda |
| AB930199.1 | S140023/2014 | 2014 | Japan |
| KP941134.1 | Keny-061/2008 | 2008 | Kenya |
| KP883206.1 | Mali-137/2008 | 2008 | Mali |
| KP882678.1 | Ghan-148/2007 | 2007 | Ghana |
| AB848012.1 | HC12016/2012 | 2012 | Japan |
| OR756445.1 | VE17426/2020 | 2020 | Viet Nam |
| DQ490535.1 | AU-1/1982 | 1982 | Japan |
| AB009626.2 | PO-3/1983 | 1983 | Japan |
| GQ479953.1 | ETD-882/2007 | 2007 | USA |
