# Supplementary material for: Whole-genome characterization and pathogenicity of novel human-porcine reassortant rotavirus strains G9P[7] and G1P[7] in China
Source: Vet Res. 2026 Jul 15;57:135. doi: 10.1186/s13567-026-01775-1 (PMC13371254; doi:10.1186/s13567-026-01775-1)
Supplement: Supplementary file 10 — Additional file 10. Porcine rotavirus strains used in the evolutionary analysis of the NSP4 gene. [file 13567_2026_1775_MOESM10_ESM.docx]

**Additional file 10 Porcine rotavirus strains used in the evolutionary analysis of the NSP4 gene.**

| Accession | Isolate | Collection Date | Geo Location |
| --- | --- | --- | --- |
| KJ126820.1 | LLP48/2008 | 2008 | China |
| KU886311.1 | HLJ | 2015 | China |
| MN102374.1 | GHA/14/2016 | 2016 | Ghana |
| ON676178.1 | SD-1/2021 | 2021 | China |
| MK597969.1 | SCLS-X1/2018 | 2018 | China |
| KC139788.1 | LL3354/2000 | 2000 | China |
| PV631384.1 | SQ-23/2023 | 2023 | China |
| GQ240627.1 | mani-362/07/2008 | 2008 | India |
| PQ724865.1 | ZT5159/2023 | 2023 | China |
| OR911933.1 | GD/2022 | 2022 | China |
| MT784855.1 | MZ-MPT-200/2016 | 2016 | Mozambique |
| PQ581891.1 | CH/10.2-20/2022 | 2022 | China |
| PQ724872.1 | ZT2130/2023 | 2023 | China |
| MK283699.1 | P830/2015 | 2015 | CZE |
| MG407654.1 | rj24598/2015 | 2015 | Brazil |
| KC580432.1 | DC1292/1980 | 1980 | USA |
| KC579689.1 | DC104/1974 | 1974 | USA |
| LC095919.1 | NT0077/2007 | 2007 | Viet Nam |
| EF672603.1 | USA/P/1974 | 1974 | USA |
| LC095952.1 | NT0621/2008 | 2008 | Viet Nam |
| JQ069177.1 | RT178-07/2008 | 2008 | Canada |
| JN258345.1 | 2007719685/2007 | 2007 | Barbados |
| KY497549.1 | PAK/42/2010 | 2010 | Pakistan |
| KC580107.1 | DC581/1979 | 1979 | USA |
| EF672575.1 | USA/D/1974 | 1974 | USA |
| ON855318.1 | F01482/2009 | 2009 | Belgium |
| MT874992.1 | NJ2012/2012 | 2012 | China |
| MF940465.1 | K71/2006 | 2006 | Korea |
| JX971578.1 | K5/2004 | 2004 | Korea |
| KF500216.1 | 174-1/2006 | 2006 | Korea |
| PP235805.1 | GDZHF/2023 | 2023 | China |
| PQ586686.1 | YNDL/2023 | 2023 | China |
| OQ743755.1 | YN-A/2021 | 2021 | China |
| KF500227.1 | C-1/2006 | 2006 | Korea |
| PV500793.1 | YNKM/2023 | 2023 | China |
| MF940576.1 | 174-1/2006 | 2006 | Korea |
| MF940630.1 | KJ11/2006 | 2006 | Korea |
| OP978247.1 | OSU/1975 | 1975 | USA |
| LC776523.1 | C-Sh/2022 | 2022 | Japan |
| LC433783.1 | TK1797/2007 | 2007 | Nepal |
| PQ127091.1 | IRN/502312/2021 | 2021 | Iran |
| PP862133.1 | Fuzhou23-93/2023 | 2023 | China |
| PP862100.1 | Pingtan21-2/2021 | 2021 | China |
| ON012983.1 | SCMY2/2021 | 2021 | China |
| ON993101.1 | SZ18442205/2018 | 2018 | China |
| ON993091.1 | GD18442033/2018 | 2018 | China |
| ON993077.1 | SC18511025/2018 | 2018 | China |
| ON993050.1 | JL18221297/2018 | 2018 | China |
| ON993026.1 | SZ18442055/2018 | 2018 | China |
| MN529657.1 | JZ1812/2018 | 2018 | China |
| LC158125.1 | LUS12-14/2012 | 2012 | Zambia |
| KY055436.1 | BUW-14-085/2014 | 2014 | Uganda |
| KX655526.1 | MUL-13-427/2013 | 2013 | Uganda |
| AB930200.1 | S140023/2014 | 2014 | Japan |
