# Supplementary material for: Whole-genome characterization and pathogenicity of novel human-porcine reassortant rotavirus strains G9P[7] and G1P[7] in China
Source: Vet Res. 2026 Jul 15;57:135. doi: 10.1186/s13567-026-01775-1 (PMC13371254; doi:10.1186/s13567-026-01775-1)
Supplement: Supplementary file 11 — Additional file 11. Porcine rotavirus strains used in the evolutionary analysis of the NSP5 gene. [file 13567_2026_1775_MOESM11_ESM.docx]

**Additional file 11 Porcine rotavirus strains used in the evolutionary analysis of the NSP5 gene.**

| Accession | Isolate | Collection Date | Geo Location |
| --- | --- | --- | --- |
| PQ323314.1 | GZ/2023 | 2023 | China |
| MK227397.1 | H14020027 | 2014 | Bangladesh |
| DQ003299.1 | HP140/2005 | 2005 | India |
| MK250434.1 | HY-1/2018 | 2018 | China |
| LC433784.1 | TK1797/2007 | 2007 | Nepal |
| MH137270.1 | SCLSHL-2-3/2017 | 2017 | China |
| LC569901.1 | PK2015-1-0001 | 2015 | Thailand |
| AY033396.1 | RMC321/2001 | 2001 | India |
| KX363314.1 | VNM/12129_48 | 2012 | Viet Nam |
| KF726043.1 | E931/2008 | 2008 | China |
| PQ141605.1 | 923E/2021 | 2021 | China |
| MH898997.1 | SCJY-5/2017 | 2017 | China |
| MF462320.1 | LNCY/2016 | 2016 | China |
| LC765817.1 | RVN17.0271/2017 | 2017 | Viet Nam |
| KF835970.1 | BP1125/2004 | 2004 | Hungary |
| LC389884.1 | R1207/2009 | 2009 | Sri Lanka |
| PQ133258.1 | NG523/2022 | 2022 | China |
| PQ299951.1 | C48-VS/2020 | 2020 | Croatia |
| MN224029.1 | Nov12-N5289/2012 | 2012 | Russia |
| KF835971.1 | BP1227/2002 | 2002 | Hungary |
| OP886872.1 | CN1P7/2021 | 2021 | China |
| KF835974.1 | BP1547/2005 | 2005 | Hungary |
| PP235806.1 | GDZHF/2023 | 2023 | China |
| MF940633.1 | KJ11/2006 | 2006 | Korea |
| FJ206094.1 | KJ212/2008 | 2008 | Korea |
| KF500195.1 | KOR/42-1 | 2006 | Korea |
| MT874993.1 | NJ2012/2012 | 2012 | China |
| MF940468.1 | K71/2006 | 2006 | Korea |
| OP978248.1 | OSU/1975 | 1975 | USA |
| GU329526.1 | CH-1/2009 | 2009 | China |
| PP100170.1 | JSNJ2019/2019 | 2019 | China |
| LC190496.1 | KKL-117/2014 | 2014 | Thailand |
| MN066881.1 | CMC_00052/2010 | 2010 | India |
| MG781041.1 | CMH-N016-10/2010 | 2010 | Thailand |
| GU199491.1 | Gottfried/1975 | 1975 | USA |
| MN066810.1 | CMC_00038/2011 | 2011 | India |
| OR772035.1 | NN485-23/2023 | 2023 | Russia |
| PQ127094.1 | IRN/502312/2021 | 2021 | India |
| PP862215.1 | Fuzhou23-93/2023 | 2023 | China |
| PP862183.1 | Pingtan21-4/2021 | 2021 | China |
| KX655505.1 | MUL-13-160/2013 | 2013 | Uganda |
| KX655516.1 | MSK-13-048/2013 | 2013 | Uganda |
| AB930201.1 | S140023/2014 | 2014 | Japan |
| KP883186.1 | Mali-135/2008 | 2008 | Mali |
| KP882603.1 | Ghan-107/2009 | 2009 | Ghana |
| KP882097.1 | Bang-090/2008 | 2008 | Bangladesh |
| MG701225.1 | DOM/3000503731/2016 | 2016 | DR |
| ON792046.1 | BTY25L/2018 | 2018 | Malawi |
| MT767407.1 | Moscow-714/2014 | 2014 | Russia |
| KU550327.1 | SS98244047/2015 | 2015 | Spain |
